# Impact of *Fkbp5* × early life adversity × sex in humanised mice on multidimensional stress responses and circadian rhythmicity

**DOI:** 10.1038/s41380-022-01549-z

**Published:** 2022-04-22

**Authors:** Verena Nold, Michelle Portenhauser, Dolores Del Prete, Andrea Blasius, Isabella Harris, Eliza Koros, Tatiana Peleh, Bastian Hengerer, Iris-Tatjana Kolassa, Michal Slezak, Kelly Ann Allers

**Affiliations:** 1 Boehringer Ingelheim Pharma GmbH & Co KG, Biberach an der Riß, Germany; 2 Ulm University, Clinical & Biological Psychology, Ulm, Germany; 3 BioMedX Institute, Heidelberg, Germany; 4 University of Manchester, Manchester, England; 5 Lukasiewicz Research Network - Polish Center for Technology Development, Wrocław, Poland

**Keywords:** Neuroscience, Genetics, Psychology

## Abstract

The cumulative load of genetic predisposition, early life adversity (ELA) and lifestyle shapes the prevalence of psychiatric disorders. Single nucleotide polymorphisms (SNPs) in the human *FKBP5* gene were shown to modulate disease risk. To enable investigation of disease-related SNPs in behaviourally relevant context, we generated humanised mouse lines carrying either the risk (AT) or the resiliency (CG) allele of the rs1360780 locus and exposed litters of these mice to maternal separation. Behavioural and physiological aspects of their adult stress responsiveness displayed interactions of genotype, early life condition, and sex. In humanised females carrying the CG- but not the AT-allele, ELA led to altered HPA axis functioning, exploratory behaviour, and sociability. These changes correlated with differential expression of genes in the hypothalamus, where synaptic transmission, metabolism, and circadian entrainment pathways were deregulated. Our data suggest an integrative role of *FKBP5* in shaping the sex-specific outcome of ELA in adulthood.

## Introduction

Stress responses are essential to adjust physiology and behaviour to recurrently changing environmental demands [1], but corrupted stress responses are a hallmark feature of psychiatric conditions [2]. The susceptibility or resilience to develop psychiatric disorders can be attributed to interactions of genetic predispositions and environmental factors [3]. Among environmental factors, early life adversity (ELA) is found to be especially detrimental given that aberrations during development will influence the affected individuals throughout life [4]. Childhood maltreatment is common in the history of many psychiatric patients and comprises experiences of physical, sexual, and emotional abuse, as well as physical and emotional neglect [5]. Such experiences during development shape disease prevalence in later life through alterations in HPA axis programming, stress coping strategies, and brain connectivity [6]. With respect to genetic predispositions, the regulation of glucocorticoid signalling is a prominent research target since glucocorticoids are a key messenger for the spread and initiation of stress-responsive signalling. This regulation is finetuned in a timing- and dose-dependent manner and depends on the individual cellular set-up such as the relative expression of glucocorticoid receptors and its regulators [7]. Expression levels of *FKBP5*, a potent negative regulator of glucocorticoid signalling, is part of this cellular identity and is itself a target of glucocorticoid-mediated gene transcription [8]. Single nucleotide polymorphisms (SNPs) inside the human *FKBP5* gene are associated with differential induction of the FKBP51 protein upon glucocorticoid stimulation [9] and add to the variability of stress perception and response in the population [10]. Carriers of the high induction allele rs1360780-A/T of *FKBP5* who suffered from ELA are more prone to develop psychiatric symptoms in later life than individuals without such preconditioning [11]. Importantly, sex-dependent differences in the interaction of *FKBP5* and life adversities have been associated to a higher prevalence of depression in females [12]. Despite the strong negative impact of psychiatric disorders on quality of life and productivity, the underlying processes linking *FKBP5* genotypes, stress regulation and pathological transitions are not fully understood. Animal models offer a possibility to investigate gene × environment interactions in a timely resolved manner. In depth analyses of laboratory mouse sequences in-house indicated numerous *Fkbp5* SNPs that vary by strain. However, no SNPs at the same location or with the same functional impact as found in humans occur naturally in rodents. This lack of an animal model suited to exploring human *FKBP5* SNPs hinders elucidation of causal relationships and mechanisms underlying disease development and progression. Therefore, we previously generated *Fkbp5*-humanised mice carrying either the risk-associated high induction AT-allele of rs1360780 or the resiliency-associated CG-allele. Initial characterisation of primary CNS-cell types derived from these mice revealed that the presence of the AT-allele results in the increased expression of *Fkbp5* upon stimulation of the glucocorticoid receptor compared to the CG-allele [7]. This initial characterisation prompted us to exploit this new model to examine the *Fkbp5* × ELA interactions on the stress response system in adulthood. We exposed AT- and CG-allele carrying mice to prolonged maternal separation stress, since this paradigm is broadly used to mimic ELA in rodents [13]. When mice reached adulthood, the performance of the HPA axis and behavioural response of *Fkbp5*-humanised mice to mild stressors were measured. Furthermore, we investigated the transcriptomic profiles in several brain regions engaged in stress processing. Lastly, astrocytes and neurons derived from human induced pluripotent stem cells (hiPSCs) were analysed for SNP-based differences in their expression profiles.

The goals of the study were to validate the *Fkbp5* × ELA model by (1) determining whether ELA would cause alterations in the offspring’s adult behaviour and physiology compared to controls, (2) determining whether risk AT-allele carriers would respond differently to ELA than CG-allele carriers, (3) assessing which pathways are involved in the adaptation to ELA in context of risk and resilience associated SNPs. A more far-reaching aim was to demonstrate that the humanised *Fkpb5* × ELA mouse model can be used to further investigate the influence of the human *FKBP5* gene variants on the risk and resilience to stress and to further elucidate their contribution to psychiatric disorders.

## Results

**Fig. 1:**
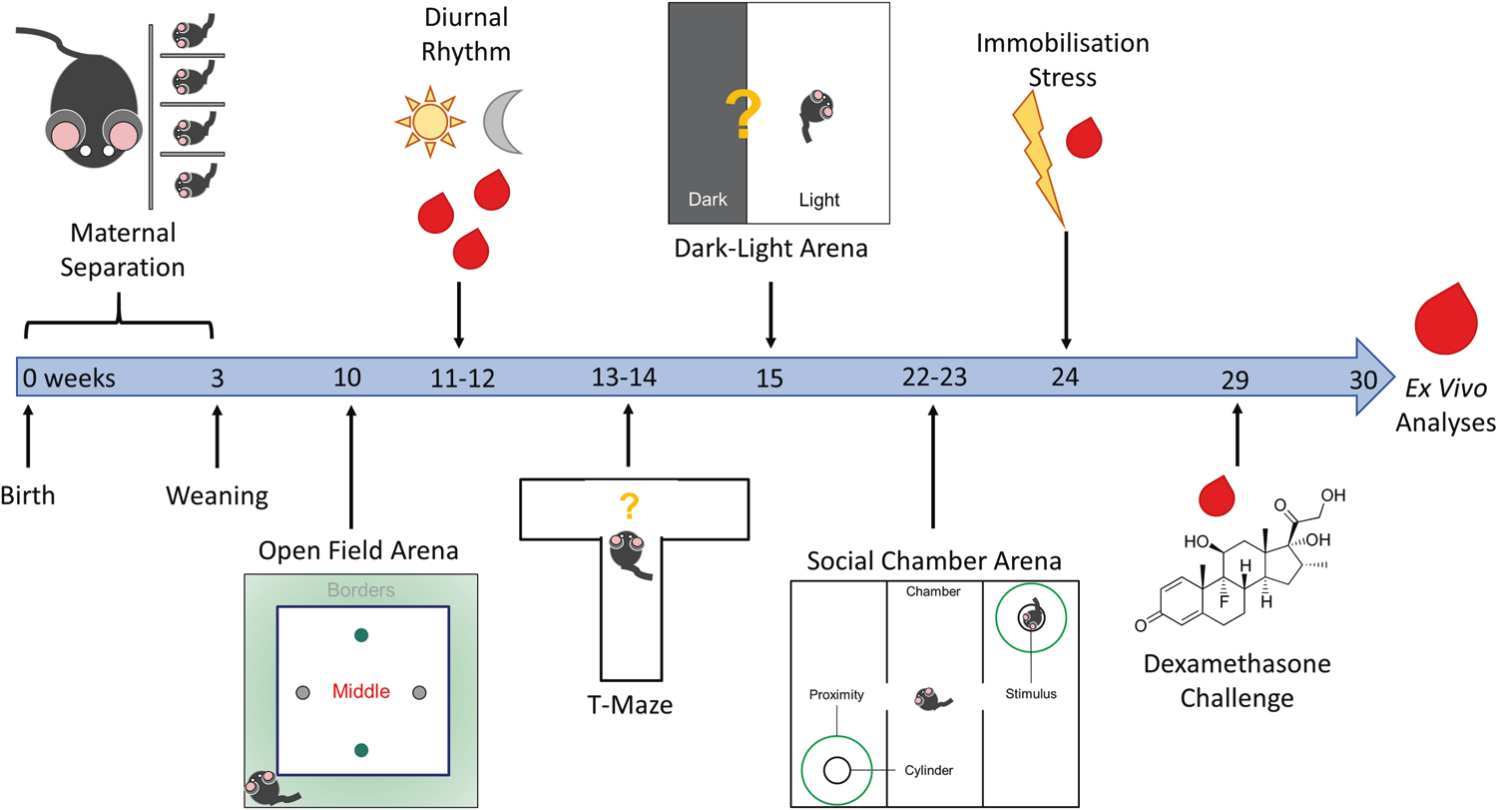
Timeline of experiments. Study overview of in vivo and ex vivo experiments during the lifetime of *Fkbp5-*humanised mice with ELA. The same timeline, except for the maternal separation, was applied to control mice in parallel.

Prolonged separation from mothers and peers was performed for the first three weeks of postnatal life to model ELA. In parallel, control mice were housed with littermates and received undisturbed maternal care until weaning. An overview of the group sizes of the cohort is provided in Table 1. On postnatal day 21, pups were weaned and grown to adulthood with physiological and behavioural examination starting at 10 weeks of age (Fig. 1). Exploration of novel environments offers an easily accessible measure of mild stress in rodents [14]. Therefore, we challenged control and ELA-exposed mice with novel situations to probe for their stress coping strategies. The same procedures were simultaneously carried out in wild type mice of both sexes to control effectiveness of the manipulations. Since the focus of this study is on the differences between the human SNPs and how these interact with ELA, the data on wild type HPA axis functioning and behaviour are visualised in Supplementary Figs. 1–5. Statistical analyses were performed jointly for males and females of all three lines to address differences between sex, ELA exposure, *Fkbp5*-genotypes and the interactions thereof. Details of the descriptive analyses, model summaries and analysis of variance (ANOVA) results are provided in Supplementary Table 1–40. Only the significant findings are indicated in the following paragraphs. A significant effect of sex × genotype × treatment interaction and significant two-way interactions in the vast majority of measured parameters were detected and are detailed in the following paragraphs.

**Table 1:** Overview of the Study Cohort.

Genotype	Early Life	Sex	*N*	Litters
CG	Control	Male	7	2
CG	Control	Female	7	3
CG	ELA	Male	11	3
CG	ELA	Female	11	3
AT	Control	Male	7	4
AT	Control	Female	5	4
AT	ELA	Male	5	2
AT	ELA	Female	10	3
WT	Control	Male	15	6
WT	Control	Female	20	5
WT	ELA	Male	17	5
WT	ELA	Female	17	6

### Early life adversity and *Fkbp5*-genotype shift and attenuate diurnal HPA axis rhythmicity

To measure the impact of *Fkbp5* SNPs in combination with ELA on the diurnal performance of the HPA axis, the plasma corticosterone concentration of samples collected at three time points was assessed. As confirmed in the wild type mice (Supplementary Fig. 1, Supplementary Tables 1–3), these timepoints were reflecting the diurnal nadir (morning), peak (evening) and one intermediate state (noon).

**Fig. 2:**
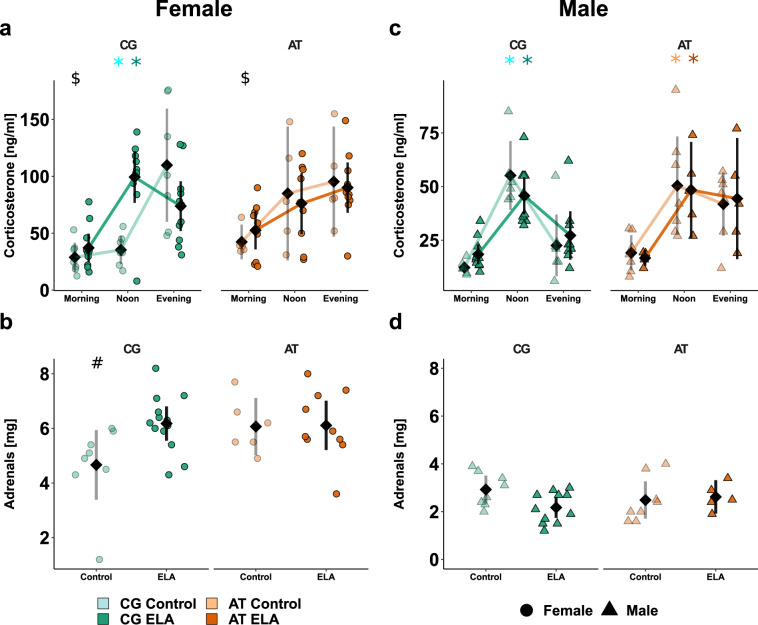
*Fkbp5*-genotype × ELA influence the unstimulated HPA axis activity in a sex-dependent manner. Individual animal data is shown alongside with the mean (black diamond) ± 95% confidence intervals to indicate statistical differences among the subgroups. Selected results of the ANOVA at group level are indicated. All descriptive statistics, model summaries, and ANOVA results are provided in Supplementary Table 1–6. Diurnal rhythmicity of corticosterone plasma levels in female (**a**) and male (**c**) *Fkbp5*-humanised controls or ELA-exposed mice. A different scale for males than females was used to make the pattern better visible. Significant diurnal rhythm was seen in CG-allele carrying female and male controls () and ELA-exposed mice (), as well as in AT-allele carrying control () and ELA-exposed males (). Morning corticosterone was higher in AT- vs. CG-allele carrying females (*p* = 0.03, $). Comparison of adrenal weights in females (**b**) and males (**d**). Female CG-allele carrying controls differ from most other subgroups (SNP × ELA × sex *p* = 0.04, #).

In control females carrying the CG-allele, the expected increase of plasma corticosterone over the course of the day was observed, with a clear peak towards the evening (Fig. 2a). Following ELA exposure, the highest concentration was instead measured at noon. The increase of plasma corticosterone levels in AT-allele carrying control females was not statistically significant, regardless of ELA exposure. In addition, the morning corticosterone levels in AT- vs. CG-allele carrying females were higher, suggesting that the levels did not fully decrease to low levels for the murine resting phase. In *Fkbp5*-humanised males, the diurnal plasma corticosterone concentration peaked towards noon with CG- vs. AT-allele carriers showing a decrease towards the evening, regardless of ELA exposure (Fig. 2c). Given that the shift in corticosterone peak was present in males of both humanised lines, this effect is likely a feature of the human gene and not of the transgenic modification. The detected diurnal amplitude of corticosterone was smaller in males than females. The adrenal weight in female AT-allele carriers and CG-allele carrying females after ELA compared to CG-allele carrying controls was increased (Fig. 2b).

No significant differences in the adrenal weights were observed among males (Fig. 2d), but male vs. female adrenal weights were significantly lower.

Taken together, female AT- vs. CG-allele carriers are genetically predisposed to less pronounced diurnal HPA axis rhythmicity and elevated corticosterone levels at time points when mice usually would rest. Lower diurnal corticosterone amplitudes and adrenal weights in males vs. females suggest a different corticosterone secretion capacity between sexes.

### Early life adversity increases responsiveness to novel environments dependent on *Fkbp5* genotype and sex

**Fig. 3:**
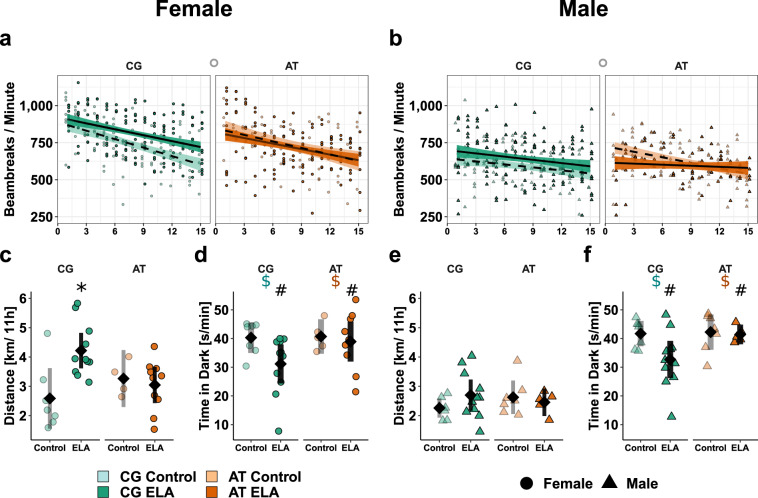
Sex × *Fkbp5*-genotype × ELA interactions alter activity in humanised mice. Individual data is shown alongside with the mean ± 95% confidence intervals to indicate statistical differences among subgroups. Selected results of the ANOVA at group level are indicated. Descriptive statistics, model summary, and ANOVA results are provided in the Supplementary Tables 11-19. Exploration activity (light beams crossing / minute) during the first 15 minutes in a novel environment in females (**a**) and males (**b**). The decrease in activity was lower in ELA-exposed than control mice (*p* < 0.05) and less in males than females (*p* = 1^−5^) given lower initial activity. AT- vs. CG-allele carrying controls tended to remain more active (°, *p* = 0.07). Total distance [km] females (**c**) and males (**e**) moved during the night. The CG-allele and ELA showed significant interaction (*p* = 0.01) that was most visible in females (∗), since females were more active than males (*p* < 1^−5^). Average time [s/min] females (**d**) and males (**f**) spent in the dark compartment. ELA-exposed mice were less in the dark than controls (#, *p* < 1^−5^), with the CG- vs. AT-allele tending to decrease the time in the dark ($, *p* = 0.08).

Exposure to novel environments as mild stress was applied to determine natural behaviour and coping strategies. First, behaviour in open field test arenas was assessed to obtain a measure of locomotor activity before (17:00–18:30), throughout (18:30–05:30) and after (05:30–06:00) the murine active phase. Overall activity within the first 15 minutes, including running and rearing, was assessed by measuring the frequency of crossing light beams (Fig. 3a). During this period, the activity decreased over time with early life condition and sex showing an interaction with time. As in wild type females (Supplementary Fig. 3, Supplementary Tables 11–16), CG control females displayed habituation in the shape of a strong decrease in activity, while the exposure to ELA led to flattening of the 15 minutes activity profile and thus slower habituation (Fig. 3a). AT-allele carrying females tended to decrease their activity less than CG-controls, regardless of early life condition. Analyses of the total nocturnal distance revealed main effects of sex and early life condition, as well as an interaction effect of ELA × genotype (Fig. 3c). While ELA-exposed female CG-allele carriers were more active than controls, AT-allele carrying females with ELA experience were indistinguishable from controls. In males, the activity measured in the open field arena (Fig. 3b, e) were similar among groups.

In the spontaneous alternations T-maze, ELA did not affect the fraction of alternations between left or right side of the maze, irrespective of genotype or sex (Supplementary Fig. 4, Supplementary Tables 20–25), suggesting no impact on working memory performance. However, ELA-exposed mice performed the task significantly faster than the respective control group. Females were quicker than males.

In the dark-light test, ELA decreased the mean time spent in the dark compartment (Fig. 3d, f). This was rather the case in CG- than AT-allele carriers but not strong enough to be detected as ELA × genotype interaction. Instead, a trend for *Fkbp5*-genotype related effects was detected, with CG- vs. AT-allele carriers spending less time in the dark.

**Fig. 4:**
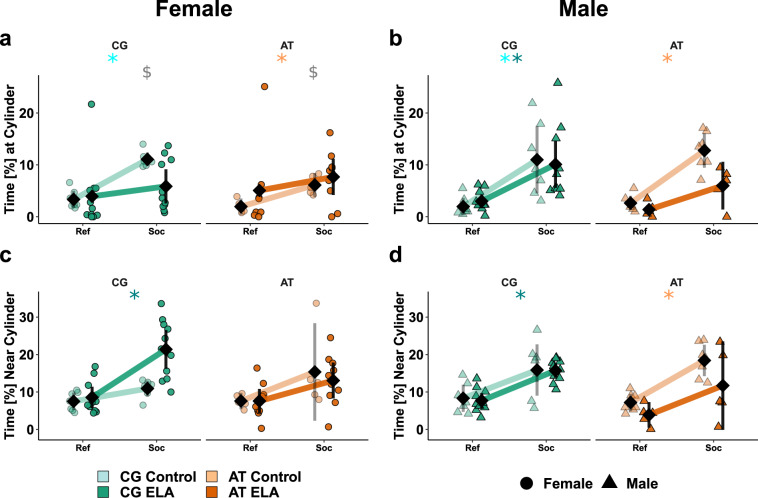
Sex × *Fkbp5*-genotype × ELA interactions alter social behaviour. Individual data is shown alongside with the mean ± 95% confidence intervals to indicate statistical differences among subgroups. Selected results at group level are indicated. Descriptive statistics, model summary, and ANOVA results are provided in the Supplementary Tables 26–31. Time [%] females (**a**) and males (**b**) spent at the cylinder with (Soc) or without (Ref) an unfamiliar mouse. Significant social preference is indicated in CG-allele carrying controls (), ELA-exposed males (), and in AT-allele carrying controls (). The preference for the social compartment (*p* < 1^−5^) was more pronounced in males (*p* = 0.01) since only AT-allele carrying males with ELA lost the preference, while both AT- and CG-allele carrying females with ELA discriminated less between the social and reference side (ELA × SNP × sex *p* = 0.02). AT- vs. CG-allele carrying female controls spent less time in social interaction (, *t*(7) = 5*, p* = 0.001). Time [%] females (**c**) and males (**d**) spent in the area surrounding the cylinder with or without a stimulus mouse. An overall preference for the social side was present (*p* < 1^−5^) that was seen in CG-allele carriers with ELA () and AT-allele carrying male controls (). AT- vs. CG-allele carriers (*p* = 0.01) and males vs. females (*p* = 0.04) with ELA spent less time on the social side. Both effects are attributable to CG-allele carrying females with ELA spending more time on the social side.

Finally, we measured social preference in the social chamber test in *Fkbp5*-humanised (Fig. 4) and wild type mice (Supplementary Fig. 5, Supplementary Tables 26–31). Pairwise comparisons of compartment effect separated by early life conditions, genotype, and sex revealed significant differences. CG-allele control females showed social preference, measured by the time the mouse spent in the nearest vicinity of the cylinder with the social stimulus (Fig. 4a). The exposure to ELA led to a decrease of this parameter, while simultaneously we observed a significant increase in the time spent in 5 cm distance from the social stimulus (Fig. 4c). Matching with the time spent, CG-allele carrying females that experienced ELA moved more distance in the area surrounding the unfamiliar mouse than controls (Supplementary Fig. 6, Supplementary Tables 32–37). Moreover, they moved faster than controls on the social, but not on the reference side. AT vs. CG-allele carrying control females spent less time interacting with the unfamiliar mouse. With ELA, AT-allele carrying females did not show social preference. The time in ‘social distance’ was similar in the AT-allele carrying controls and ELA-exposed females and indicated no preference for the social side. In contrast to females, the social preference was not affected by ELA in male CG-allele carriers. In AT-allele carrying males with ELA vs. controls, less time was spent with or close by the social stimulus (Fig. 4b, d).

Overall, the data on behavioural responses to mild stress suggest that the effects of ELA on these read outs depend on the genetic variants of *Fkbp5* × sex.

### HPA axis responses are stronger in females than males

**Fig. 5:**
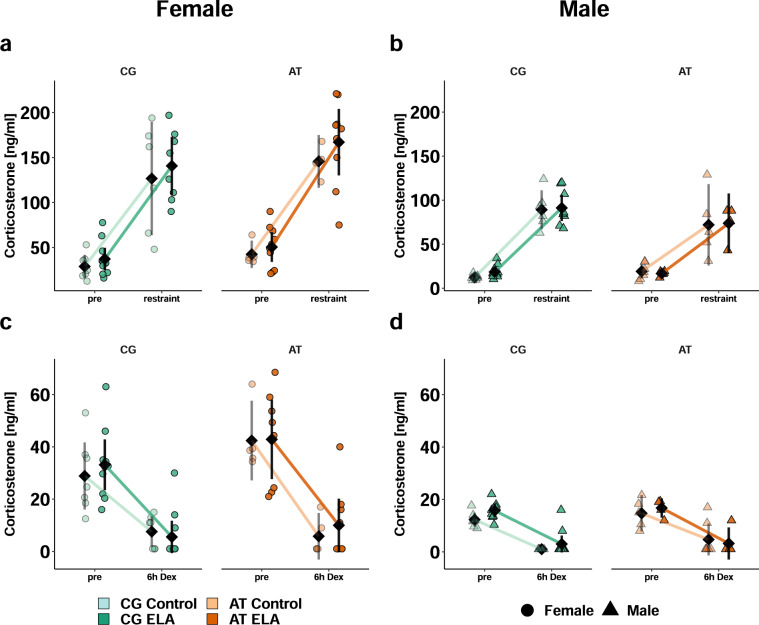
Stimulated HPA axis reactivity in *Fkbp5*-humanised females is greater than in males. Individual animal data are shown alongside with the mean ± 95% confidence intervals (black) to indicate statistical differences among subgroups. Selected results at group level are indicated. Descriptive statistics, model summaries, and ANOVA results are provided in the Supplementary Tables 1 and 7-10. Acute responsiveness of the HPA axis assessed by comparison of plasma corticosterone levels before and five minutes after restraint stress in females (**a**) and males (**b**). Stress induced an increase in corticosterone (*p* < 1^−5^) with females responding more than males (*p* < 1^−5^). Overall, females vs. males (*p* < 1^−5^) and mice with ELA vs. controls had higher corticosterone levels (*p* = 0.04). Suppression of endogenous corticosterone production six hours after dexamethasone injection (*p* < 1^−5^) was more pronounced in females (**c**) than males (**d**, *p* < 1^−5^).

To probe the HPA axis reactivity to acute induction and negative feedback, we measured plasma corticosterone after five minutes of restraint stress and six hours after a single intraperitoneal injection of the synthetic glucocorticoid dexamethasone, respectively. In all mice, corticosterone increased in response to restraint stress without a differential effect of genotype. The slope was steeper in females (Fig. 5a) compared to males (Fig. 5b). Overall corticosterone levels were higher in ELA-exposed mice than controls and higher in females than males. Similarly, all mice responded to dexamethasone with reduced corticosterone levels, suggesting a suppression of the endogenous corticosterone secretion. *Post hoc* analyses revealed that the slope of decrease was overall steeper in females than males (Fig. 5c, d).

In summary, the responsiveness of the HPA axis is preserved in *Fkbp5*-humanised mice.

### Transcription in stress-responsive brain regions is affected by *Fkbp5* × ELA

To identify transcriptional differences that could be related to differences in behaviour and HPA axis physiology of *Fkbp5*-humanised mice × ELA, mRNA sequencing and analyses of differential gene expression were carried out. Given the behavioural and physiological findings that female AT- vs. CG-allele carriers differ, while little to no effects were seen in males, next generation sequencing was limited to *Fkbp5*-humanised females to identify potential transcriptomic correlates of the differences in vivo. The analyses focused on hypothalamus, ventral and dorsal hippocampus as brain regions engaged in stress regulation [15]. In the SNP-comparison among controls, more differentially expressed genes (DEGs) were found in the hypothalamus (579), followed by ventral (41) and dorsal (2) hippocampus (Table 2). Among ELA-exposed individuals, more DEGs between the SNPs were detected than in controls, underscoring the interaction of ELA × *Fkbp5*-genotype. Looking at the effect of ELA, fewer differences were detected in AT-allele carriers (114) than in CG-allele (903) carriers. This matches to the behaviour and HPA axis data, where few additional impact of ELA to the differences introduced by the AT-allele were seen.

**Table 2:** Counts of differentially expressed genes in subgroups of *Fkbp5*-humanised female mice.

Comparison	Tissue	Direction	Control	ELA
*Fkbp5*-genotype	Hypothalamus	AT > CG	349	561
	Hypothalamus	CG > AT	230	855
	Ventral Hippocampus	AT > CG	18	468
	Ventral Hippocampus	CG > AT	23	457
	Dorsal Hippocampus	AT > CG	1	798
	Dorsal Hippocampus	CG > AT	1	844

Comparison	Tissue	Direction	CG	AT
Early life condition	Hypothalamus	ELA > Con	410	4
	Hypothalamus	Con > ELA	195	29
	Ventral Hippocampus	ELA > Con	16	0
	Ventral Hippocampus	Con > ELA	10	0
	Dorsal Hippocampus	ELA > Con	145	27
	Dorsal Hippocampus	Con *>* ELA	127	54

Adopting knowledge from the SNP effects in humans, the overlap and uniqueness of the identified DEGs were analysed for nomination of potential resiliency- or vulnerability-related genes. Genes linked to CNS-development such as *Mab21l2*, *Gart* and *Lipt2* were spotted as potentially vulnerability-related and were changed in opposite directions, with AT- vs. CG-allele carriers displaying a lower expression.

A second analysis focussing on gene clusters related to neurological disorders using a two-step core and comparison analysis of the commercial software Ingenuity (Qiagen) confirmed that the ELA-responsive DEGs in both mouse lines have an impact on neurological and psychiatric symptoms (Supplementary Fig. 7). In eight of the shown 30 deregulated clusters e.g., comprising ‘congenital neurological disorder’ or ‘learning’, the effects were opposite between AT- vs. CG-allele carriers.

In sum, the counts of DEGs and their accordant vs. discordant overlap suggest that the *Fkbp5* × ELA interaction on gene expression may have relevance for neurologic and psychiatric symptomatology.

### The AT-allele and ELA reduce CNS communication but increase metabolism

To identify how the DEGs might be linked to disorders via their role in cellular pathways, their over-representation in metabolism and signalling-related pathways listed in the Kyoto Encyclopaedia of Genes and Genomes (KEGG) was assessed. The analyses revealed significantly altered pathways in the hypothalamus and ventral hippocampus (Table 3). The direction of change between *Fkbp5*-genotypes differed dependent on function, with pathways related to neuronal communication rather showing a downregulation, and pathways related to metabolism rather showing an upregulation in AT- vs. CG-allele carriers. In the hypothalamus, the most significantly downregulated pathways included circadian entrainment, regulation of synaptic plasticity via long-term potentiation and depression as well as activity of dopaminergic and cholinergic synapses together with changes in calcium, cAMP, and oxytocin signalling. In the ventral hippocampus, reduced expression of genes related to synaptic communication in AT- vs. CG-allele carriers was repeated. Especially in the ELA subgroup, lower expression of genes related to cAMP signalling and dopaminergic synapses were found in AT-allele carriers compared to CG-allele carriers. Independent of strain, ELA was linked to lower expression of transcripts related to endocannabinoid and circadian entrainment relative to controls. For genes in pathways related to metabolism, such as ‘protein digestion and absorption’ in the hypothalamus or ‘ribosome’ activity and ‘oxidative phosphorylation’ in the ventral hippocampus of controls, higher expression in AT- vs. CG-allele carriers was observed.

**Table 3:** Enriched KEGG pathways in *Fkbp5*-humanised females.

Tissue	Group	Comparison	KEGG Pathway	*p*	Mean
Hypothalamus	Overall	AT vs. CG	Dopaminergic synapse	0.002	−0.012
			Circadian entrainment	0.003	−0.943
			ECM-receptor interaction	0.007	2.702
			Oxytocin signalling pathway	0.020	−0.115
			Long-term potentiation	0.022	−0.189
			Ras signalling pathway	0.025	−0.028
			Protein digestion & absorption	0.029	2.051
			Cholinergic synapse	0.030	−0.958
			Long-term depression	0.037	−0.893
			Calcium signalling pathway	0.041	−0.779
			cAMP signalling pathway	0.049	1.689
Ventral Hippocampus	overall	AT vs. CG	Ribosome	0.007	2.496
			Phosphatidylinositol signalling	0.012	−0.470
			Inositol phosphate metabolism	0.014	−0.418
			cAMP signalling pathway	0.024	−0.043
			Oxytocin signalling pathway	0.042	−0.804
			Aldosterone synthesis & secretion	0.043	−0.818
	Controls	AT vs. CG	Ribosome	0.014	2.252
			Oxidative phosphorylation	0.030	1.950
	ELA	AT vs. CG	Dopaminergic synapse cAMP	0.042	−0.794
			signalling pathway	0.043	−0.781
	overall	ELA vs. Con	Dopaminergic synapse	0.019	−0.218
			Circadian entrainment	0.021	−0.173
			Endocannabinoid signalling	0.032	−0.912

The DEGs detected in the dorsal hippocampus were not significantly overrepresented in individual KEGG pathways.

*p = p*-value of geometric mean, *Mean* = mean difference of fold changes, *ECM =* extracellular matrix, *Ras* = rat sarcoma, *cAMP* = adenosine 3’,5’-cyclic monophosphate, *AT* = *Fkbp5* rs1360780-A/T (high induction) allele, *CG* = *Fkbp5* rs1360780-C/G (resilience) allele.

The mRNA of neurons and astrocytes derived from hiPSCs of rs1360780 carriers was used to qualitatively validate the SNP-dependence of the observed differences in an independent expression system. In both cell types, similar SNP-based expression differences like in the *Fkbp5*-humanised mice were seen, which could indicate that less synaptic communication in AT- vs. CG-allele carriers is not an artefact from the process of generating the transgenic mice. However, the distribution within the pathways differed between hiPSC and mouse derived samples. More DEGs in the upstream vs. downstream members of the circadian entrainment pathway were seen in the *Fkbp5*-humanised mice, while in the hiPSCs rather downstream targets were changed (Supplementary Fig. 8). The expression patterns in astrocytes vs. neurons were more similar to the patterns seen in mice.

The KEGG pathway analyses imply that ELA and the AT-allele both lead to less entrainment of diurnal HPA axis and sleep-wake rhythmicity. This may interact with the decreased ability of AT- vs. CG-allele carriers to process incoming inputs via synaptic communication.

### Lower glucocorticoid sensitivity of the Hippocampus is modulated by *Fkbp5*

**Fig. 6:**
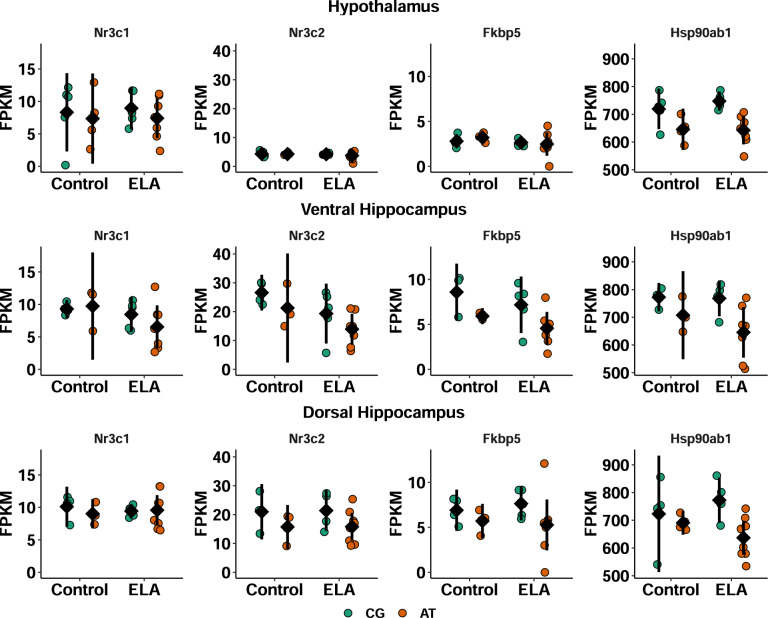
Brain region specific expression levels of glucocorticoid signalling regulators. Expression levels [FPKM] of the glucocorticoid receptor (*Nr3c1*, GR), mineralocorticoid receptor (*Nr3c2*), *Fkbp5* and heat shock protein 90 (*Hsp90ab1*) of individual female AT- or CG-allele carriers that experienced ELA or undisturbed maternal care (control) are visualised alongside with the mean ± 95% confidence intervals. Plots are shown separate for hypothalamus (**top**), ventral hippocampus (**middle**) and dorsal hippocampus (**bottom**). Descriptive statistics and an overview of the significant model terms in the ANOVA are provided in the Supplementary Tables 38 and 39. In all three regions, AT- vs. CG-allele carriers expressed less *Hsp90* (hypothalamus *p* = 0.0006, ventral hippocampus *p* = 0.009, dorsal hippocampus *p* = 0.009). *Fkbp5* was lower expressed in the hippocampi of AT- vs. CG-allele carriers (*p* = 0.01) and *Nr3c2* was lower expressed in the ventral hippocampus of ELA-exposed vs. control mice (*p* = 0.03) as well as lower expressed in the dorsal hippocampus of AT- vs. CG-allele carriers (*p* = 0.03).

To estimate how much impact the potentially altered glucocorticoid exposure due to differences in circadian entrainment and synaptic signalling might exert on the hypothalamus, ventral hippocampus, and dorsal hippocampus, the expression levels of genes related to glucocorticoid signalling were compared (Fig. 6). This analysis provides insights in the likelihood of the brain regions to respond to glucocorticoid stimulation. While expression levels of the glucocorticoid receptor (*Nr3c1*) and heat shock protein 90 (*Hsp90ab1*) were comparable between all three brain regions, the mineralocorticoid receptor (*Nr3c2*) and *Fkbp5* were less expressed in hypothalamus than hippocampus. Moreover, the AT- vs. GC-allele was associated with a lower *Fkbp5* expression in the hippocampus and less *Hsp90ab1* in all three brain regions.

Considering the gene functions, the hypothalamus appears to be more sensitive to glucocorticoid receptor mediated signalling than the hippocampus, with CG- vs. AT-allele hippocampi being more protected.

### DEGs in *Fkbp5*-humanised mice are related to differences in vivo

The decreased cerebral expression of genes related to synaptic communication in AT- vs. CG-allele carriers might be a compensatory mechanism to prevent excessive excitation. To test whether the expression levels of the identified DEGs and *Fkbp5* could be linked to the observed behavioural and physiological differences, tissue-wise correlation analyses were carried out. For each brain region, the top 10 correlations are provided in the supplements (Supplementary Fig. 9, Supplementary Table 40, the full list of correlations will be provided upon request). In all three brain regions, the majority of DEGs correlated with *Fkbp5*. In the hypothalamus, gap junction protein *β* 1 (*Gjb1*) showed a correlation with the time spent in the dark compartment of the test arena, while the membrane-associated tyrosine-specific kinase 1 (*Pkmyt1*) and the nicotinic acetylcholine receptor subunit 7 (*Chrna7*, regression shown in Supplementary Fig. 10) were linked to morning corticosterone levels. This could indicate an association between some hypothalamic DEGs and differences in HPA axis functioning and behaviour. The limitation of the transcriptomic analyses to females was useful in identifying physiological correlates of the transcriptome.

The correlation analyses suggest a linkage between expression levels of *Fkbp5* and DEGs in brain regions relevant for stress processing.

## Discussion

The present study has demonstrated a gene × environment interaction in novel *Fkbp5*-humanised mice, indicating that the model is suited to investigate the effects of ELA in the context of risk- and resiliency-related SNPs. Early life adversity elicited by maternal separation has differential impact on adult physiology and behaviour based on genetic predisposition imparted by *Fkbp5* alleles. This is demonstrated by changes in locomotor, social, and anxious behaviour. Additionally, diurnal corticosterone rhythmicity is moderately altered as seen at a functional level via HPA axis profiling and on molecular levels through altered gene expression in the circadian entrainment pathway. Differential gene expression in brain regions relevant to stress regulation shows an enrichment for pathways linked to neural communication and brain disorders. Many of the DEGs are correlated with *Fkbp5* levels. In the tests utilised here, the impact of *Fkbp5* SNPs and ELA was greater in females than males.

These stronger effects of *Fkbp5* × ELA in female compared to male mice match previously reported sexual dimorphism in responsiveness to ELA in animals [16] and was discussed in humans [17]. Notably, ELA and sex hormones both influence maturation kinetics and thus the development of cerebral regions implicated in glucocorticoid regulation [18]. The interactions of the SNP rs1360780, sex, and ELA observed in the mice presented here and in humans [11, 12] could thus be explained by the regulatory capacity of *Fkbp5* on glucocorticoid signalling. Based on sex-dependent correlations between *FKBP5* levels and depression and anxiety scores as well as with nadir cortisol levels, *FKBP5* was suggested as a female-specific biomarker for prolonged cortisol load and the associated risk of psychiatric disorders [19]. In line with this correlation, we observed associations between genotype and nadir corticosterone levels in *Fkbp5*-humanised mice, with AT-allele carrying females displaying higher morning glucocorticoid levels than CG-allele carrying females. The sexual dimorphism in the effect of ELA indicate that the novel *Fkbp5*-humanised mouse model offers the possibility to further investigate the networking of ELA, sex, and disease-related SNPs.

In addition, the data provide mechanistic insights into how *Fkbp5* SNPs may contribute to the shaping of overall physiology and the stress response system. As negative modulator of glucocorticoid receptor maturation, *Fkbp5* holds the potential to inhibit glucocorticoid signalling. At the same time, its expression depends on recent glucocorticoid exposure since *Fkbp5* itself harbours glucocorticoid response elements [8]. The higher induction of the AT-allele in CNS cell types of *Fkbp5*-humanised mice upon glucocorticoid stimulation could thus be expected to result in stronger or longer inhibition of subsequent glucocorticoid signalling [7]. In vivo, this stronger induction of inhibitory potential via *Fkbp5* in AT-allele carriers could lead to dampened negative feedback to the HPA axis and a prolonged interval of elevated glucocorticoid levels, as reflected by elevated morning corticosterone levels in AT- vs. CG-allele carrying females. The negative feedback loop is furthermore critical for the maintenance of oscillation patterns and function [20]. The reduction in the complexity of ultradian fluctuation and the resulting decreased variability of HPA axis reactivity in AT-allele carriers could decrease their flexibility to respond to novel environments. Behavioural evidence of this differential responsiveness could include the alterations in light-dark box testing, locomotor habituation, and abnormal social behaviour as seen in this study. In humans, differences in HPA axis responsiveness to environmental stimuli, e.g., in the Trier Social Stress Test, between human AT- and CG-allele carriers has been demonstrated [10]. The findings imply that *Fkbp5* genotype dependent regulation of ultradian HPA axis activity might be a core molecular mechanism that contributes to the variability seen in human stress responsiveness, which ultimately plays a role in distinction between healthy adaptation or pathological alteration in the aftermath of stress [21].

Another environmental stimulus that can affect glucocorticoid rhythms is the light-dark cycle [22]. One commonly investigated manifestation of this circadian rhythmicity is the pronounced increase of glucocorticoids prior to awakening [23]. Mechanistically, the ability to detect light in the retinal ganglia and to signal this via the suprachiasmatic nucleus to the periphery is a crucial trigger for the awakening response [24]. In AT- vs. CG-allele carriers, flatter diurnal glucocorticoid profiles were paralleled by lower expression of circadian entrainment related genes even though histological analyses of the eyes (data not shown) indicated no differences in the ability to detect light. This underscores the relevance of self-maintaining feedforward and feedback loops in regulating overall physiology throughout the day. While external light signals can synchronise individuals to a 24 hours cycle [25], the internal gene expression driven clock seems to define the shape of the circadian glucocorticoid profile and thus when and how strong individuals are likely to respond to challenges. In humans, modulation of the cortisol awakening response was reported to influence their performance during the upcoming day and was dependent on the anticipation of challenges [26]. The awakening response is used clinically to identify individuals with certain personality traits that are vulnerable to develop psychiatric disorders [27], and for the diagnosis of depression [28]. Besides impaired awakening responses, differences in kinetic and responsiveness of the HPA axis, e.g. to acute stress or dexamethasone exposure, between psychiatric patients and healthy controls have been demonstrated [29]. In the present study, no disfunction of HPA axis responsiveness was observed, which indicates that the combination of ELA and genetic predisposition via the AT-allele of *Fkbp5* alone might not be sufficient to cause full pathology. This is in agreement with the Research Domain Criteria framework proposing a continuum between’normal’ and’pathological’ which needs to be better understood in order to alleviate symptoms. Accordingly, the transition to pathology occurs over a lifetime and is a multidimensional process shaped by numerous genetic and environmental factors that introduce subtle changes which jointly alter networking of physiological systems [30]. As in humans, the *Fkbp5*-humanised mouse model demonstrates changes in basal HPA axis activity dependent on genotype and early life experience, with more prominent effects in females than males. These alterations in non-stimulated HPA axis functioning were suggested to have an impact on sleep-wake states, responsiveness to environmental stimuli and vice versa [31]. In the long run, insufficient adaptation could contribute to allostatic load and finally development of disorders [21]. However, the cumulative stress load in this study was low since the animals were not exposed to any severe or chronic stressors during later life.

**Fig. 7:**
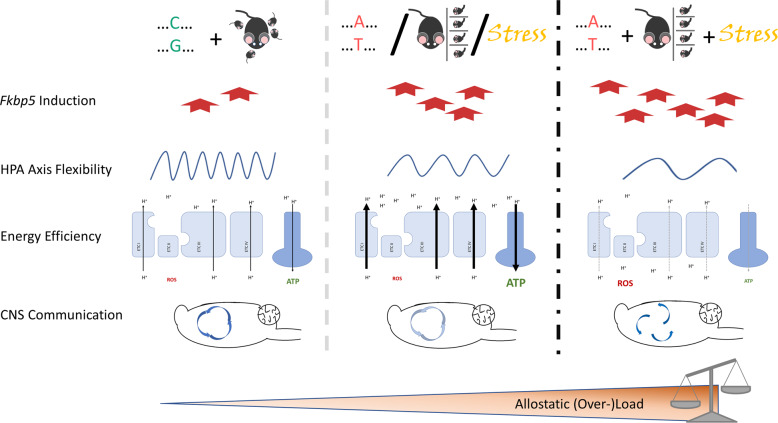
Proposed sequence of alterations in the stress response system on cellular and brain circuit level in health, allostasis, and allostatic overload. The normal induction of *Fkbp5* upon challenge in CG-allele carriers with undisturbed maternal care allows for dynamic ultradian and circadian rhythms of the HPA axis (**left**). In parallel, the electron transport chain (ETC) in the mitochondrial membrane produces energy in the form of adenosine-triphosphate (ATP) and few reactive oxygen species (ROS), while brain regions involved in stress regulation such as hypothalamus, hippocampus, pre-frontal cortex and amygdala engage in interconnected communication. Carriers of the AT-allele, or individuals exposed to early life adversity or mild chronic stress show signs of allostatic load (**centre**). The affected individuals display a higher induction of *Fkbp5* and an attenuated rhythmicity of the HPA axis. The associated increase in nadir glucocorticoid levels is linked to higher expression of genes related to oxidative phosphorylation, resulting in elevated mitochondrial respiration and ATP production, and to a lower expression of genes involved in synaptic communication. In the proposed triple-hit condition, a further increase in the levels of *Fkbp5* could interfere with the negative feedback to the HPA axis and delay the termination of the stress response (**right**). As consequence of prolonged stress, the ETC might suffer from wear and tear resulting in a decreased efficiency in ATP production combined with elevated ROS generation and oxidative stress. Moreover, the reduced communication between stress-regulating brain regions could manifest in uncoupling of the brain circuits and asynchronous neural signalling. The here described *Fkbp5-*humanised mice will support future work to validate this scenario.

Nevertheless, the *Fkbp5* × ELA model shows indications of changes in the psycho-immune-neuro-endocrine system that are commonly seen in response to chronic stress. Reduced expression of immediate early genes as markers of plasticity in the prefrontal cortex and hippocampus as well as elevated mitochondrial respiration in response to repeated mild stress during adulthood was previously reported [32]. In the present study, the increased expression of genes related to oxidative phosphorylation in the hippocampus of AT- vs. CG-allele carriers is an interesting parallel, as is the reduction of genes related to synaptic communication. Reduced neural communication and plasticity might become maladaptive since dendritic retraction has been described to render the hippocampus more vulnerable to neurotoxic or metabolic challenges [33, 34]. The longer the time window of decreased plasticity and increased vulnerability exists, the higher is the likelihood of a co-incidental high metabolic demand. Stressful situations only transiently elevate energetic demands while simultaneously decreasing the neuronal supply with glucose [35]. Unique stress events may thus not cause irreversible harm to the hippocampus, and AT-allele carriers might even benefit from their inherent higher expression of mitochondrial genes. Under prolonged exposure to glucocorticoids, increased oxidative phosphorylation in AT-allele carriers might produce excessive amounts of neurotoxic reactive oxygen species which may damage the hippocampus. Findings of this study imply more glucocorticoid signalling in the hippocampus of AT- relative to CG-allele carriers since the glucocorticoid signalling inhibitor *Fkbp5* had a lower expression level while nadir corticosterone levels were increased in female AT- vs. CG-allele carriers. Cumulatively, this mechanism could contribute to the loss of hippocampal volume in stress-related disorders such as depression and would explain why AT-allele carriers are more prone to develop disorders than CG-allele carriers [36]. The proposed sequence of alterations on cellular and circuitry level from healthy to allostatic load and allostatic overload conditions is outlined in Fig. 7. Assessment of behaviour and physiologic read outs in *Fkbp5*-humanised mice that experienced both, ELA and more severe or chronic stress paradigms, would resolve these questions.

Moreover, the combination of *Fkbp5* SNPs and ELA with simultaneous or sequential stress hits could enable prediction of and intervention at critical transition points during the development and progression of psychiatric symptoms.

## Conclusion

The cumulative load of genetic predisposition, unfavourable environmental influences during development, and repeated exposure to stressful events increases the prevalence of psychiatric disorders in affected individuals. The glucocorticoid-induced expression of *Fkbp5* is a hub for integrating lifetime and recent stressful experiences. Simultaneously, *Fkbp5* modulates responsiveness to acute stressors as negative modulator of glucocorticoid signalling. The naturally occurring *Fkbp5*-SNPs in laboratory rodents do not feature comparable functional effects as rs1360780 in humans, where the AT- vs. CG-allele is more strongly induced by glucocorticoids and linked to the aetiology of psychiatric disorders. To enable studying in more detail the mechanistic impact of the human SNP on stress physiology and the aetiology of psychiatric disorders, *Fkbp5*-humanised mouse lines carrying either the AT- or CG-allele of this SNP were generated. Characterisation of the *Fkbp5* × ELA mouse model showed mechanistic and face validity with aspects of psychiatric disorders. Female AT- vs. CG-allele carriers after ELA showed attenuated diurnal rhythmicity of glucocorticoids, lower activity, and less responsiveness to novel environments. On a molecular level, reduced expression of genes related to circadian entrainment and synaptic communication as well as increased expression of genes related to mitochondrial respiration between AT- vs. CG-allele carriers imply a genetic predisposition of their psycho-immune-neuro-endocrine system to allostatic changes reported in mild chronic stress settings. Since ELA lead to decreased circadian entrainment in the hippocampus, which in turn influences the circadian entrainment in the hypothalamus, the combination of ELA and *Fkbp5* SNPs could synergistically modify the HPA axis to respond less to stimuli. Given that dynamic variability in glucocorticoid levels and plasticity are required for adaptation to challenges, this predisposition increases the risk of an unsuccessful resolution of allostatic loads and thus elevates the risk of developing stress-related disorders. In combination with severe or chronic stress exposure, the observed *Fkbp5* × ELA interactions likely contribute to the aetiology of stress-related pathology. First indication of the transcriptomic findings in mice being translatable to man were obtained using hiPSCs differentiated into astrocytes and neurons but require further investigation due to the small sample size available. Taken together, we are confident that this novel animal model will contribute to more comprehensive analyses of *FKBP5*-induced alterations in the stress response network that causally lead to the development of pathology.

## Supplementary information

Supplementary Material

## Data Availability

Raw and aggregated data will be made available upon request. The hiPSC data sets will be made available upon request. The accession code for the murine NGS data set on NCBI's Sequence Read Archive is PRJNA743189.

## Code availability

The R code to process, analyse, and visualise the data will be made available upon request.

## References

[1] Selye H (1950). Stress and the general adaptation syndrome. BMJ.

[2] de Kloet ER, Joels M, Holsboer F (2005). Stress and the brain: from adaptation to disease. Nat Rev Neurosci.

[3] Matosin N, Halldorsdottir T, Binder EB (2018). Understanding the molecular mechanisms underpinning gene by environment interactions in psychiatric disorders: The FKBP5 model. Biol Psychiatry.

[4] Opendak M, Gould E, Sullivan R (2017). Early life adversity during the infant sensitive period for attachment: programming of behavioral neurobiology of threat processing and social behavior. Developmental Cogn Neurosci.

[5] MacMillan HL, Fleming JE, Streiner DL, Lin E, Boyle MH, Jamieson E (2001). Childhood abuse and lifetime psychopathology in a community sample. Am J Psychiatry.

[6] Nemeroff CB (2016). Paradise lost: The neurobiological and clinical consequences of child abuse and neglect. Neuron.

[7] Nold, V, Richter, N, Hengerer, B, Kolassa, I-T & Allers, KA FKBP5 polymorphisms induce differential glucocorticoid responsiveness in primary CNS cells – first insights from novel humanized mice. Eur J of Neurosci. (2020). 10.1111/ejn.14999. 10.1111/ejn.14999 PMC7894319 33030232

[8] Pirkl F, Buchner J (2001). Functional analysis of the hsp90-associated human peptidyl prolyl cis/trans isomerases FKBP51, FKBP52 and cyp40 1 1edited by r. Huber. J Mol Biol.

[9] Binder EB, Salyanika D, Lichtner P, Wochnik GM, Ising M, Pütz B (2004). Polymorphisms in FKBP5 are associated with increased recurrence of depressive episodes and rapid response to antidepressant treatment. Nat Genet.

[10] Ising M, Depping A-M, Siebertz A, Lucae S, Unschuld PG, Kloiber S (2008). Polymorphisms in the FKBP5 gene region modulate recovery from psychosocial stress in healthy controls. Eur J Neurosci.

[11] Dackis MN, Rogosch FA, Oshri A, Cicchetti D (2012). The role of limbic system irritability in linking history of childhood maltreatment and psychiatric outcomes in low-income, high-risk women: Moderation by FK506 binding protein 5 haplotype. Dev Psychopathol.

[12] Van Zomeren-Dohm AA, Pitula CE, Koss KJ, Thomas K, Gunnar MR (2015). FKBP5 moderation of depressive symptoms in peer victimized, post-institutionalized children. Psychoneuroendocrinology.

[13] Millstein RA, Holmes A (2007). Effects of repeated maternal separation on anxiety- and depression-related phenotypes in different mouse strains. Neurosci Biobehav Rev.

[14] Campos AC, Fogaca MV, Aguiar DC, Guimaraes FS (2013). Animal models of anxiety disorders and stress. Rev Brasileira de Psiquiatria.

[15] Floriou-Servou A, von Ziegler L, Stalder L, Sturman O, Privitera M, Rassi A (2018). Distinct proteomic, transcriptomic, and epigenetic stress responses in dorsal and ventral hippocampus. Biol Psychiatry.

[16] Goodwill HL, Manzano-Nieves G, Gallo M, Lee H-I, Oyerinde E, Serre T (2018). Early life stress leads to sex differences in development of depressive-like outcomes in a mouse model. Neuropsychopharmacology.

[17] Davis EP, Pfaff D (2014). Sexually dimorphic responses to early adversity: Implications for affective problems and autism spectrum disorder. Psychoneuroendocrinology.

[18] Moisan M-P (2021). Sexual dimorphism in glucocorticoid stress response. Int J Mol Sci.

[19] Lee RS, Mahon PB, Zandi PP, McCaul ME, Yang X, Bali U (2018). DNA methylation and sex-specific expression of FKBP5 as correlates of one month bedtime cortisol levels in healthy individuals. Psychoneuroendocrinology.

[20] Walker JJ, Spiga F, Waite E, Zhao Z, Kershaw Y, Terry JR (2012). The origin of glucocorticoid hormone oscillations. PLoS Biol.

[21] McEwen BS (2003). Mood disorders and allostatic load. Biol Psychiatry.

[22] Reppert SM, Weaver DR (2002). Coordination of circadian timing in mammals. Nature.

[23] Wüst S, Wolf J, Hellhammer DH, Federenko I, Schommer N, Kirschbaum C (2000). The cortisol awakening response - normal values and confounds. Noise Health.

[24] Fu Y, Zhong H, Wang M-HH, Luo D-G, Liao H-W, Maeda H (2005). Intrinsically photosensitive retinal ganglion cells detect light with a vitamin a-based photopigment, melanopsin. Proc Natl Acad Sci.

[25] Aschoff J, Gerecke U, Wever R (1967). Desynchronization of human circadian rhythms. Jpn J Physiol.

[26] Fries E, Dettenborn L, Kirschbaum C (2009). The cortisol awakening response (CAR): facts and future directions. Int J Psychophysiol.

[27] Vrshek-Schallhorn S, Doane LD, Mineka S, Zinbarg RE, Craske MG, Adam EK (2012). The cortisol awakening response predicts major depression: predictive stability over a 4-year follow-up and effect of depression history. Psychological Med.

[28] Huber TJ, Issa K, Schik G, Wolf OT (2006). The cortisol awakening response is blunted in psychotherapy inpatients suffering from depression. Psychoneuroendocrinology.

[29] Coppen A, Abou-Saleh M, Milln P, Metcalfe M, Harwood J, Bailey J (1983). Dexamethasone suppression test in depression and other psychiatric illness. Br J Psychiatry.

[30] Stapelberg N, Pratt R, Neumann DL, Shum DHK, Brandis S, Muthukkumarasamy V (2018). From feedback loop transitions to biomarkers in the psycho-immune-neuroendocrine network: Detecting the critical transition from health to major depression. Neurosci Biobehav Rev.

[31] Koch C, Leinweber B, Drengberg B, Blaum C, Oster H (2017). Interaction between circadian rhythms and stress. Neurobiol Stress.

[32] Nold V, Sweatman C, Karabatsiakis A, Böck C, Bretschneider T, Lawless N (2019). Activation of the kynurenine pathway and mitochondrial respiration to face allostatic load in a double-hit model of stress. Psychoneuroendocrinology.

[33] Conrad CD. Chronic stress-induced hippocampal vulnerability: the glucocorticoid vulnerability hypothesis. Rev in the Neurosci. 19 (2008). 10.1515/revneuro.2008.19.6.395. 10.1515/revneuro.2008.19.6.395 PMC2746750 19317179

[34] McEwen BS, Gould EA, Sakai RR (1992). The vulnerability of the hippocampus to protective and destructive effects of glucocorticoids in relation to stress. Br J Psychiatry.

[35] Harrell C, Gillespie C, Neigh G (2016). Energetic stress: The reciprocal relationship between energy availability and the stress response. Physiol Behav.

[36] Sheline YI, Sanghavi M, Mintun MA, Gado MH (1999). Depression duration but not age predicts hippocampal volume loss in medically healthy women with recurrent major depression. J Neurosci.

