# Supplementary material for: Impact of Fkbp5 × early life adversity × sex in humanised mice on multidimensional stress responses and circadian rhythmicity
Source: Mol Psychiatry. 2022 Apr 22;27(8):3544–55. doi: 10.1038/s41380-022-01549-z (PMC9708571; doi:10.1038/s41380-022-01549-z)
Supplement: Supplementary file 1 — Supplementary Material [file 41380_2022_1549_MOESM1_ESM.pdf]

Supplementary Material

Supplementary material for the article

Impact of *Fkbp5* × Early Life Adversity × Sex in Humanized Mice on Multidimensional Stress Responses and Circadian Rhythmicity

written by Verena Nold, Michelle Portenhauser, Dolores Del Prete, Andrea Blasius, Isabella Harris, Eliza Koros, Tatiana Peleh, Bastian Hengerer, Iris-Tatjana Kolassa, Michal Slezak, and Kelly Allers.

List of Figures

1 Baseline Plasma Corticosterone Levels in Wild Type Females Fluctuate more than in Males. . . . 13

2 Stimulated Plasma Corticosterone Levels in Wild Type Females Fluctuate more than in Males. . . . 14

3 ELA Increases Exploration Behavior but not Overall Activity in Wild Type Mice 15

4 ELA Shortens the Time to Completion of the T-Maze Test in ***Fkbp5***-Humanized and Wild Type Mice 16

5 Wild Type Mice Maintain Social Preference Despite ELA . . 17

6 Activity of ***Fkbp5***-Humanized Mice in Proximity to Unfamiliar Mice 18

7 Comparison of ELA-Induced Changes in Neurological and Developmental Disease Pathways between ***Fkbp5***-Genotypes 19

8 Differential Expression of Genes Related to Circadian Entrainment in ***Fkbp5***-Mice and in hiPSC-Derived Astrocytes and Neurons of Human ***FKBP5***-SNP Carriers 19

9 Top 10 Correlated DEGs with ***Fkbp5***, ***in vivo*** and ***ex vivo*** Readouts 23

10 Regression of Morning Corticosterone Levels and ***Chrna7*** . . 23

List of Tables

1 Descriptive Corticosterone Concentration [ng/ml] in *Fkbp5*-Humanized and Wild Type Mice 20

2 Model Summary Diurnal Corticosterone 22

3 ANOVA Diurnal Corticosterone 24

4 Descriptive Adrenal Weight [mg] 25

5 Model Summary Adrenal Weight 25

6 ANOVA Adrenal Weight 26

7 Model Summary Dexamethasone Suppression 27

8 ANOVA Dexamethasone Suppression Test 28

9 Model Summary Restraint Stress Corticosterone 29

10 ANOVA Restraint Stress Corticosterone 30

11 Descriptive Beambreaks Novel Environment *Fkbp5*-Humanized and Wild Type Mice 31

12 Model Summary Beambreaks Habituation OFT 33

13 ANOVA Beambreaks Habituation OFT 34

14 Descriptive Nocturnal Distance [m] 35

15 Model Summary Nocturnal Distance OFT 35

16 ANOVA Nocturnal Distance OFT 36

17 Descriptive Average Time [s/min] in the Dark Compartment 37

18 Model Summary Dark-Light-Test 37

19 ANOVA Time in the Dark Compartment during Dark-Light Test 38

20 Descriptive Alternations [%] T-Maze 39

21 Model Summary Alternations T-Maze 39

22 ANOVA T-Maze Alternations 40

23 Descriptive Latency to Completion of the T-Maze 41

24 Model Summary Latency Completion T-maze 41

25 ANOVA T-Maze Latency Completion 42

26 Descriptive Social Interaction [% time] 43

27 Model Summary Interaction Time SCT 44

28 ANOVA Percent Interaction Time SCT 45

29 Descriptive Social Distance [% time] 46

30 **Model Summary Distant Observation Time SCT** 47
31 **ANOVA Percent Distant Observation Time SCT** 48
32 **Descriptive Distance [m] Social Zone** 49
33 **Model Summary Distance Social Zone** 50
34 **ANOVA Distance [m] Social Zone** 51
35 **Descriptive Speed [mm/sec] Social Zone** 52
36 **Model Summary Speed Social Zone** 53
37 **ANOVA Speed [mm/sec] Social Zone** 54
38 **Descriptive Statistics of Glucocorticoid Signaling Regulating Genes Separated by Brain Region, *Fkbp5*-Genotype and Early Life Condition** 55
39 **Significant Results of the ANOVA on Tissue-Wise Expression of Glucocorticoid Signaling Regulators in Dependence of *Fkbp5*-Genotype × Early Life Condition** 57
40 **Top 10 Pearson Correlations between DEGs, Behavior, Physiology and *Fkbp5* Expression** 58

1 Methods

1.1 Generation of Transgenic Mice

Two novel transgenic mouse models were created by and are publicly available at Taconic Biosciences, carrying either the cytosine (C)/guanine (G) variant at position 3622 in the human *FKBP5* gene (C57BL/6NTac-Fkbp54571 (FKBP5) Tac) or the high-induction adenine (A)/thymine (T) version of rs1360780 (C57BL/6NTac-Fkbp5tm4570 (FKBP5) Tac). In short, the murine *Fkbp5* was exchanged with the human *FKBP5* coding region, keeping the 3' and 5' UTR of the mouse. Full details of the method are available at [1]. Homozygote mice were bred in-house to be used in the experiments.

1.2 Animal Husbandry

Standard laboratory conditions were adhered to (20-24°C, 45-55% humidity, 12-hour light/dark cycles (sunrise 6:00, sunset 18:00), *ad libitum* access for standard laboratory chow and water, enrichment (wooden block, red plastic shelter house and tube and paper stripes) provided). Mice had not been used in any other study prior to sacrifice. All animal experiments were performed under allowance of the regional council for animal welfare (Regierungspräsidium Tübingen, Baden-Württemberg, Germany, licence VVH 17-009) and in compliance with directive 2020/63/EU and ARRIVE guidelines. Sample size estimation was based on empirical knowledge regarding measurement accuracy or reproduction errors of *in vivo* and *ex vivo* methods (noise) and the expected effect sizes. Since we are the first to characterize these novel transgenic mice, no data was available for exact effect size estimations. Instead, published and in-house observed effect sizes of stress manipulations that were deemed to be biologically relevant were used as reference. As result of these considerations and based on our previous experience with animal experiments, n = 8 was agreed upon to be a suitable trade off between power for effect detection and the amount of animals required. Scheduled mating was used for breeding of the animals. On the day of birth, the litters were assigned to control or maternal separation in a way that group sizes were balanced as far as possible. Each group was assigned more than one litter to counteract for potential litter effects. Sex balance and equal litter sizes were not enforced since culling of littermates / offspring would introduce counfounding stressors and unnecessary suffering. As a result, and since litter sizes and sex ratio within litters are non deterministic, final group sizes varied. After testing for equivalence, the WT animals of the AT- and CG-allele strains were pooled which resulted in double the number of the transgenic groups.

1.3 Maternal Separation

Separation from mothers and litter mates at different times of the day for three hours starting from post-natal day two until 21 was carried out. During separation, heating pads were placed below the cages to keep the pups warm despite the lack of nesting material and low amount of saw dust. Mothers were kept at the other side

of the room in their home cages. Maternal behaviour during separation and after reunion was observed to decrease after the first few separation sessions. Blinding of the experimenter was not possible given these obvious manipulations. After weaning on post-natal day 21, adolescent mice were group-housed until an age of 6 weeks. Afterwards, if not required earlier due to aggressive behavior, animals were single housed.

1.4 Behavioral Test Battery

Locomotion, dark-light preference, sociability and spatial working memory was assessed after mice were grown up to young adults. An overview of the timelines of the behavioral test battery is provided in Fig. 1. Within measurement sessions, mouse strains were mixed but controls and ELA-exposed mice as well as males and females were separated to avoid olfactory or auditory cues being transferred between groups, introducing distress or confounding effects. The experiments were first performed in males (controls, then ELA-exposed) and subsequently in females. Assignment to arenas or order of measurements within the day was randomized and for the social chamber and T-maze test experimenters were blinded regarding test groups. Arenas were extensively cleaned between each measurement and between groups. One day pause was kept to eliminate potentially distracting olfactory cues from the room.

1.4.1 Open Field Test

A maximum of 30 arenas of 45 by 45 cm size were evenly illuminated with 267 lx, water gel and food pellet were placed around the borders of the center zone and one handful of saw dust from each mouse' home cage were distributed inside the arena. Mice were brought to the measurement room at least two hours before the session for recovery from transport and habituation to the room. Shortly before 17:00, the actimot (TSE Systems, Bad Homburg, Germany), which detects movement of animals via breaking of light beams in x, y, z direction, was switched on, mice were placed in the middle of the measurement chambers and their locomotion was recorded over night until 07:00 to obtain measures of their activity in the light and dark phase (lights off 18:00, lights on 06:00).

1.4.2 Dark Light Test

Measurement of preference for the dark or light compartment were also performed with the actimot system, while a 1/3 of the arena was kept dark (2.2 lx) using a black plastic house with a circular door to allow mice to freely travel between both compartments. At beginning of the measurement, mice were placed in the dark compartment facing the corner away from the door. Experiments were performed between 08:00 to 11:00 in the morning and lasted for 30 minutes.

1.4.3 Three Social Chamber Test

Sociability was tested in an arena divided into three compartments of equal size (60×40×22 cm, Ugo Basile) with sliding doors between the compartments. The left and right section contained a mesh cylinder (7 cm in diameter, 15 cm height). Mice were habituated to the measurement room 1 hour prior testing and experiments were performed in the morning from 7:00-12:00. The arena was evenly illuminated with 23 lx. After recording the 5 minutes habituation phase of the tested mouse to the arena, an unfamiliar stimulus mouse was placed in one of the cylinders. Choice of side was evenly distributed across groups. Stimulus mice were juvenile, of the same sex as the tested mouse, habituated to the cylinder and used twice per day with 1-hour break between measurements. Behavior of the tested mouse was videotaped for 10 minutes and analyzed using an automated tracking software (TopScan CleverSys Inc., USA). Main readouts were the time spent, the amount of entries, the activity during the visit measured by distance and speed, the latency to first enter and the latency to end the first visit. This was assessed for the chamber as a whole and a zone surrounding the cylinders. As secondary readouts, locomotion and immobility were measured.

1.4.4 Alternations T-Maze

Mice were moved to the testing room the day before their performance in the T-maze was assessed. At beginning of the measurement, the mouse was placed in the starting box for 5 seconds before the door to the arena was opened. In the first trial, the animal was either forced to enter the right or the left arm by closing the door to the respective other arm. Starting sides were evenly distributed across sexes, early life conditions and mouse lines. Every mouse was tested once and had to complete 14 trials consisting of entering one arm, closing the door to the non-chosen arm, returning of the animal to the starting zone and opening all passages to enable free choice of side for the next trial. The maximal allowed duration was set to 14 minutes and if an animal completed less than 7 trials it was excluded from further analysis. This was the case for 1 CG-allele carrying male control. Dimensions of the arena were 20 cm height, 8.5 cm corridor width, 30 cm lengths of each arm, 54 cm length of the starting zone. The test room was illuminated with 230 lx while above the T-maze light intensity was set to 50 lx. To enable spatial discrimination, navigation objects with differing shape and color were placed outside of the left and right arm of the arena.

1.5 HPA-Axis Performance

Blood was sampled from the *vena saphena* by immobilizing the mouse (Broome Rodent Restrainers, Harvard Apparatus, Cat.No.52-0460, MA 01746, USA), shaving and anointing the left leg and stinging into the vein with a lancet (Solofix, B. Braun, Cat.No. 6182003, Melsungen, Germany). Blood droplets were collected in K2-EDTA-containing capillaries (Microvette, Sarstedt, Cat.No.16.444, Nümbrecht, Germany) and stored on ice prior to centrifugation at 20000 g for 20 min at 4°C. The whole process from cage opening to collection of the last drop was carried out

within less than 1.5 minutes to avoid a procedure-associated rise of corticosterone [2]. Plasma aliquots were frozen immediately.
For tracking of basal diurnal rhythmicity, blood was collected in the morning (06:45 – 07:15), afternoon (12:45 – 13:15) and evening (18:45 – 19:15). To obtain a measure of plasma corticosterone levels after stress, on another day at about the same time when the morning blood sample was drawn, mice were kept inside the restrainer for 5 minutes before puncturing the vein. Negative feedback to the HPA-axis was investigated by comparing rise or fall of plasma corticosterone levels between morning and 6 hours after injection of saline (NaCl 0.9%, B. Braun, Cat.No. FREU950) or with 0.001 mg/kg dexamethasone (DexaHexal 4 mg/ml diluted in saline, Hexal, Holzkirchen, Germany) two days after saline injection. An overview of blood sampling time points is provided in Fig. 1. Concentrations of corticosterone were quantified using an enzyme-linked immunosorbent assay (DetectX Corticosterone Enzyme Immunoassay Kit, Cat.No. CEA540Ge, Abor Assays, TX 77494, USA) following the manufacturer's instructions. The data on morning corticosterone levels showed strong accordance between replicates and was therefore pooled for each mouse.

1.6 Gene Expression

1.6.1 Tissue Collection

Mice were sacrificed in the morning under isoflurane anesthesia by rapid decapitation. Organs were collected within 10 minutes after death and immediately stored in cooled RNAlater or for histology in 4% formaldehyde supplemented with 20% sucrose over night.

1.6.2 Generation of hiPSCs

Lines were derived from healthy patients genotyped for FKBP5 SNP rs1360780 and *FKBP5* InDel rs9470080CNV. In both cases, the AT genotype corresponds to the 'high induction', or 'risk' allele, while the CG genotype corresponds to 'low induction' or 'resilience' allele. Lines were derived from peripheral blood mononuclear cells collected from 2 females and 2 males homozygote for the AT- or CG-allele, with even distribution of both genotypes. Reprogramming was performed with episomal plasmids [3]. Comparison of the genome wide CNV in the parental material and the emanated hiPSC showed no chromosomal aberrations. Pluripotency markers were detected immunocytochemically.

1.6.3 hiPSC differentiation

All hiPSC lines were cultured in mTSER1 (Stem Cell Technologies, Cat.No. 058509) on Matrigel Matrix High Concentration (Corning, Cat.No. 354263). Neural induction was performed based on a published protocol [4] with a few modifications. hiPS cells were maintained in Matrigel coated vessels, with mTeSR1 media and split by

passing complete colonies using a non-enzymatic approach (EDTA, Versene Solution). hiPSCs were dissociated to single cells with Accutase (Stem Cell Technologies, Cat.No. 07920) and plated at 3 × 106 cells/well to allow the embryoid body (EB) formation in Neural Induction Media (NIM) + 10 μM Y-27632 (Stem Cell Technologies, Cat.No. 72308). They were allowed to attach for at least overnight, and then the medium was replaced by NIM (without Y-27632), consisting of a 1:1 mix of N2 supplement (Life Technologies, Cat.No. 17502048) in DMEM/F12 (Life Technologies, Cat.No. 31331028) and B27 supplement (Life Technologies Cat.No. 17504044) in Neurobasal (Life Technologies Cat.No. 21103049), supplemented with 10 muM SB431542 (Millipore, 616461) and 1 μM Dorsomorphin (Tocris Bioscience, Cat.No. 3093). In days in vitro (DIV) 1-4, NIM was replaced twice a day. On day 4, the EB suspension were made and moved with a 5 ml serological pipette into a 6-well Clear Flat Bottom Ultra Low Attachment Multiple Well Plates (Corning, Cat.No. 3741), and cultured in NIM replaced daily for 10 days. On 10 DIV, the EBs were plated on tissue culture plates coated with Matrigel. On DIV 14-16, the neuroepithelial sheet was detached from the plate using STEMdiff Neural Rosette Selection Reagent (Stem Cell Technologies, Cat.No. 05832). From the following day until DIV 27 cultures were grown in Neuronal Maintenance Media (N2B27 supplemented with 20 ng/μl hFGF) replaced daily or on alternate days. Between DIV 17 and 30, any non-neural differentiation present was removed by passaging with STEMdiff Neural Rosette Selection Reagent, and the neural cultures were then dissociated to single cells using Accutase. When cultures reached 80%–90% confluency, they were passaged again until a final passage between DIV 33-40, when they were plated for long-term culture, after which N2B27 medium was replaced every second day. At DIV 60 and 90, in each line, cells were detached with Accutase from several wells and filtered through 40 μm cell strainer (Corning, Cat.No. 352340) for FACS sorting evaluation.

1.6.4 FACS sorting

The media from 4 wells per line, containing astrocyte-neuron co-cultures were removed and the RLT buffer was added to wells for the RNA extraction. In parallel, at least 1 well per line (limited by the number of wells containing differentiated cells, variable between lines) was proceeded for separation of astrocytes and neurons from co-cultures using the positive selection approach with anti-CD44 (BD-bIoscience, Cat.No. 555478) antibodies [5]. Cells were gently detached from the well surface with Versene (3-5 minutes at 37 °C), to avoid the epitope damage. Mechanical dissociation with p1000 pipette (5 times gentle up- and down strokes) was applied for obtaining single cells suspension. Cells were counted and up to 3×105 cells were incubated in flow cytometry (FC) wash buffer consisting of 1% FBS, 1× penicilin-streptomycin, nuclease free water and RNasin Plus RNase inhibitor 0.2 U/μl. Next, cells were incubated in the FC wash buffer (2 h at 4 °C) containing FITC-coupled anti-CD44 antibody (1 to 80 BD Pharmingen Cat.No. 555478) or its isotype control (FITC Mouse IgG2b, κ Isotype Control, BD Pharmingen Cat.No. 555478). After incubation, cells were washed in wash buffer (RNAase-free PBS, pH 7.4 + 0.2 U/μl RNase inhibitor), spin down (300 g × 3') and resuspended in 500 μl of wash buffer. Separation was performed on FACSAria Machine (ZMBH, Heidelberg) at 4 °C using 100 μm nozzle (optimized for droplet stream). Based on side scatter pulse width and

height (SSC-W and SSC-H), 200000-300000 events of singlets were sorted directly to Low binding tube coated with FBS overnight (Corning, CLS3207-250EA), spun down (400 g × 10' at 4 °C) and the pellet was resuspended in 600μ of Qiazol Lysis Reagent (Qiagen, Cat.No. 79306) and kept at -80 °C before sequencing.

1.6.5 Next Generation Sequencing

RNA was isolated using RNeasy Plus kit (Qiagen Cat.No. 74192) following the manufacturer's recommendations. RNA purity was checked spectrophotometrically using the NanoPhotometer (IMPLEN, CA, USA) and QIAxpert(Qiagen). Concentration was measured using Qubit RNA Assay Kit in Qubit 2.0 Flurometer (Life Technologies, CA, USA), while integrity was assessed using the standard sensitivity RNA kit (Cat.No. DNF-471, Advanced Analytical) on a Fragment Analyzer (Thermo Fisher Scientific, Langenselbold, Germany) and RNA Nano 6000 Assay Kit of the Bioanalyzer 2100 system (Agilent Technologies, CA, USA). High quality RNA samples with RIN >7.5 were eligible for further processing.
A total amount of 1 μg RNA per sample was used as input material for the RNA sample preparations. Enrichment of mRNA from eukaryotic organisms was performed using oligo(dT) beads from NEBNext Poly(A) mRNA Magnetic Isolation Module (Cat.No. E7490L, NEB, USA). Subsequently, sequencing libraries were generated using NEBNext Ultra II Directional RNA Library Prep Kit for Illumina (Cat.No. E7770L, NEB, USA) following manufacturer's recommendations. Briefly, fragmentation was carried out using divalent cations under elevated temperature in NEBNext First Strand Synthesis Reaction Buffer (5×). First strand cDNA was synthesized using random hexamer primer and M-MuLV Reverse Transcriptase (RNaseH-). Second strand cDNA synthesis was subsequently performed using DNA Polymerase I and RNase H. In the reaction buffer, dNTPs with dTTP were replaced by dUTP. Remaining overhangs were converted into blunt ends via exonuclease/polymerase activities. After adenylation of 3' ends of DNA fragments, NEBNext Adaptor with hairpin loop structure were ligated to prepare for hybridization.
In order to select cDNA fragments of preferentially 250-300 bp in length, the library fragments were purified with AMPure XP beads (Cat.No. A63987 Beckman Coulter, Beverly, USA). Then 3 μl USER Enzyme (NEB, USA) was used with size-selected, adaptor-ligated cDNA at 37°C for 15 min followed by 5 min at 95°C before PCR. Then PCR was performed with Phusion High-Fidelity DNA polymerase, Universal PCR primers and Index (X) Primer. At last, products were purified (AMPure XP beads) and library quality was assessed using the Agilent High Sensitivity DNA Kit (Cat.No. 5067-4626) on the Agilent Bioanalyzer 2100 system (Agilent Technologies, CA, USA).
The clustering of the index-coded samples was performed on a cBot Cluster Generation System (Cat.No. SY401-2015, Illumina) using TruSeq PE Cluster Kit v3-cBot-HS (Cat.No. PE-401-3001, Illumia) according to the manufacturer's instructions. After cluster generation, the libraries were sequenced on a NovaSeq 6000 Illumina platform using NovaSeq 6000 S2 Reagent Kit v1.5 cat. 20028314 -(300 cycles) and 150 bp paired-end reads were generated (minimum 12 Gb and 40 M).
RNA-Seq reads were aligned to the mouse genome using Hisat2 software, version 2.1.0 with the corresponding Ensembl GRCm38.p6 reference genome (http://www.ensembl.org). Confirmation of genotyping was done by aligning the NGS

reads to the human reference genome GRCh38.p13. Sequenced read quality and duplications were checked with FastQC software, version 0.11.9 and alignment quality metrics were calculated using Samtools flagstat software, version 1.10. Gene and transcripts expression profiles were quantified using Cuffquant and Cuffnorm, version 2.2.1 and GTF file from the Ensembl (v. 100) database to obtain Fragments Per Kilobase Million mapped reads (FPKM). Library preparation, sequencing and initial data processing was carried out at Intelliseq (Poland).

1.7 Statistical Analyses

Data processing and analysis was carried out using R (version 4.0.2). For analysis of read outs with repeated measurements (open field, dark-light and social chamber test, HPA-axis performance), nested models using early life condition group, mouse strain and sex as between factors and compartment or time point as within subject component were defined and their quality was inspected visually and using one-point cross validation (R packages *nlme* and *afex*). Data without a temporal or spatial component were modeled linearly. Confidence intervals for the coefficient estimates were obtained using the non-centrality parameter method and the Greenhouse-Geiser method for approximation of the degrees of freedom was applied for nested models [6]. Analysis of variance and effect size estimation were performed using partial sum of squares type II (R packages *car* and *effectsize*). Besides the generalized eta squared ($g\eta^2$), the partial epsilon squared ($p\epsilon^2$) effect size were reported to reduce potential bias by small sample size [7, 8, 9]. In addition, the relative explanation of variance was assessed (R packages *MuMIn* and *r2glmm*). If significant model terms were suggestive, pairwise two-sided *post hoc* tests with Tukey contrasts were performed (package *emmeans*). Effect sizes in the descriptive analyses between subgroups were computed as Cohen's d estimates using pooled variance.
Regarding data obtained from next generation sequencing, the following additional analysis steps were performed: The obtained data was filtered for tissue-wise median and mean expression to be above 1 FPKM. In addition, selection criteria for fold changes and signal-to-noise ratios bigger than $\pm$ 30% and $\pm$ 1.5 were applied, respectively. Comparisons were made between control and ELA-exposed females independent of strain as well as separate for the subgroups of AT- and CG-allele carriers. Furthermore, putative differences between AT- and CG-allele carriers independent of early life experiences, and within the control and maternally separated subset were investigated. To all transcripts where the row-wise t-test was significant, a false-discovery rate filter of 10% was applied and only genes of which the related transcripts indicated fold changes in the same direction were considered as differentially expressed genes (DEGs) in subsequent analyses. Based on the association of the CG-allele with resiliency and the AT-allele with risk to develop disorders, DEGs which were unique to CG-allele carriers when comparing effects of early life conditions (coping) were labeled as potentially resiliency-associated genes, while an overlap of DEGs from the early life comparison with DEGs from the SNP-comparison in the control subgroup were labeled as potential vulnerability-related genes. In addition, transcripts were the 2-way ANOVA suggested an interaction of early life condition $\times$ *Fkbp5*-genotype at an α level of 5% were included in gene set enrichment analyses.

Using Ingenuity (IPA, Qiagen), the FPKM values of the listed DEGs were subjected to 'core analysis' of ELA vs. control for the AT- and CG-allele carrying subgroup applying a threshold of absolute fold changes bigger than 1.5. The results of these 'core analyses' were entered into a 'comparison analysis' to investigate the deregulation of genes due to ELA between strains. The 'comparison analysis' was limited to the term 'Diseases and Biological Functions', and further limited to sub-categories of neurological relevance. The z-score p-value was set to <0.0001. The final comparison list was then filtered to the top 10 results, ordered by z-score.
Pearson correlations were computed tissue-wise for the normalized expression levels of *Fkbp5* and the DEGs with HPA-axis and behavior-related read outs. The cutoff for meaningful correlations was *a priori* set to $> |0.6|$. Among those correlations, the FDR was fixed to 5%.
Generally applicable gene-set enrichment analyses (package *gage*) for metabolic pathways listed in the Kyoto Encyclopedia of Genes and Genomes (KEGG) were performed and visualized in case of significant over representation (package *pathview*). For comparison to the hiPSC-derived astrocytes and neurons, the genes listed in the circadian entrainment pathway (hsa004713) were extracted, filtered based on the above-mentioned effect size and significance criteria and visualized without prior checks on pathway enrichment due to the limitations in sample size.

References

[1] Nold, V., Richter, N., Hengerer, B., Kolassa, I.-T. & Allers, K. A. FKBP5 polymorphisms induce differential glucocorticoid responsiveness in primary CNS cells – first insights from novel humanized mice. *European Journal of Neuroscience* (2020). URL https://doi.org/10.1111/ejn.14999.

[2] Small, T. W. *et al.* Stress-responsiveness influences baseline glucocorticoid levels: Revisiting the under 3 min sampling rule. *General and Comparative Endocrinology* **247**, 152–165 (2017). URL https://doi.org/10.1016/j.ygcen.2017.01.028.

[3] Chou, B.-K. *et al.* A facile method to establish human induced pluripotent stem cells from adult blood cells under feeder-free and xeno-free culture conditions: A clinically compliant approach. *STEM CELLS Translational Medicine* **4**, 320–332 (2015). URL https://doi.org/10.5966/sctm.2014-0214.

[4] Shi, Y., Kirwan, P. & Livesey, F. J. Directed differentiation of human pluripotent stem cells to cerebral cortex neurons and neural networks. *Nature Protocols* **7**, 1836–1846 (2012). URL https://doi.org/10.1038/nprot.2012.116.

[5] Yuan, S. H. *et al.* Cell-surface marker signatures for the isolation of neural stem cells, glia and neurons derived from human pluripotent stem cells. *PLoS ONE* **6**, e17540 (2011). URL https://doi.org/10.1371/journal.pone.0017540.

[6] Steiger, J. H. Beyond the f test: Effect size confidence intervals and tests of close fit in the analysis of variance and contrast analysis. *Psychological Methods* **9**, 164–182 (2004). URL https://doi.org/10.1037/1082-989x.9.2.164.

[7] Kelley, T. L. An unbiased correlation ratio measure. *Proceedings of the National Academy of Sciences* **21**, 554–559 (1935). URL https://doi.org/10.1073/pnas.21.9.554.

[8] Olejnik, S. & Algina, J. Generalized eta and omega squared statistics: Measures of effect size for some common research designs. *Psychological Methods* **8**, 434–447 (2003). URL https://doi.org/10.1037/1082-989x.8.4.434.

[9] Allen, R. *Statistics and Experimental Design for Psychologists* (WORLD SCIENTIFIC (EUROPE), 2017). URL https://doi.org/10.1142/q0019.

2 Figures and Tables

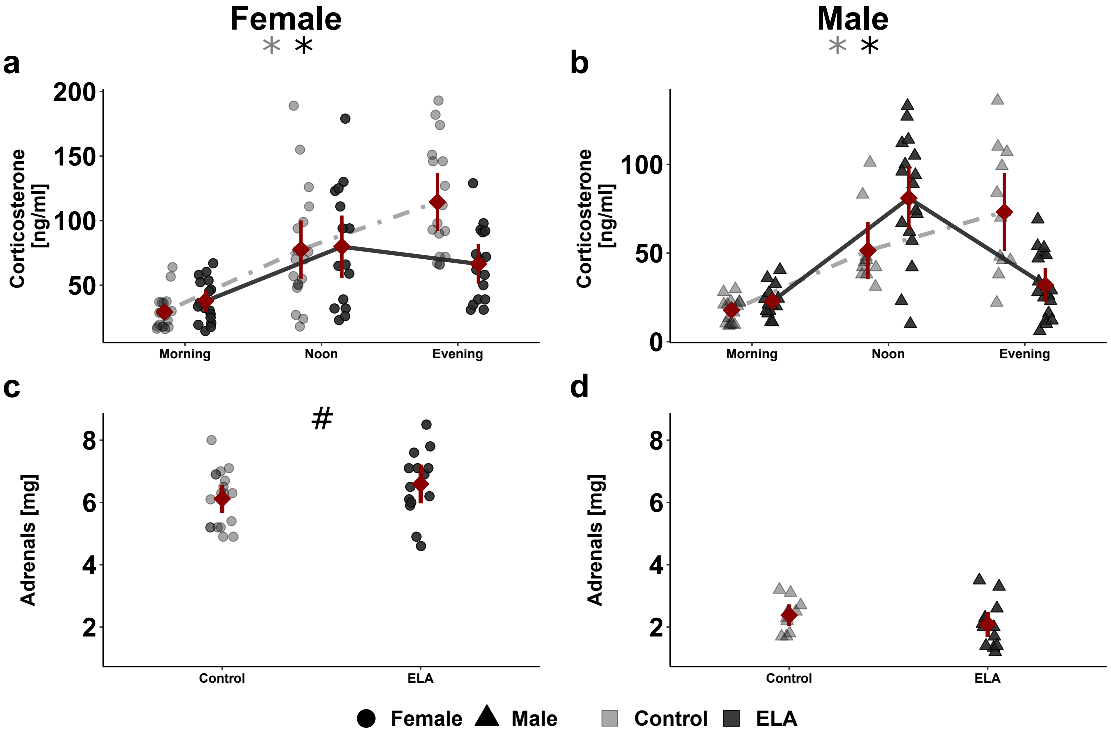

Supplementary Figure 1: **Baseline Plasma Corticosterone Levels in Wild Type Females Fluctuate more than in Males.**
Individual animal data (grey scale) is shown alongside with the mean ± 95% confidence interval (red) to indicate statistical differences. Selected findings in the ANOVA are indicated in addition, while all descriptive statistics, model summaries and ANOVA outcomes for diurnal corticosterone are provided in Tab. 1, Tab. 2, Tab. 3, while Tab. 4, Tab. 5 and Tab. 6 contain the data for adrenal weight. Diurnal rhythmicity of corticosterone plasma levels in female (**a**) and male (**b**) control mice peaks in the evening ($*$) while at noon in ELA-exposed mice ($*$). A different scale for males than females was used to make the pattern better visible but males displayed lower levels than females ($p < 1^{-5}$). CG-allele carrying females controls and ELA-exposed individuals resemble wild type females of the respective groups, while both subgroups of *Fkbp5*-humanized males resemble wild type males exposed to ELA. Adrenal weights in wild type females (**c**) are higher than in males (**d**, $p < 1^{-5}$) and higher than in CG-allele carrying female controls (#, SNP $\times$ ELA $\times$ sex $p = .04$) while adrenal weights in all males are similar.

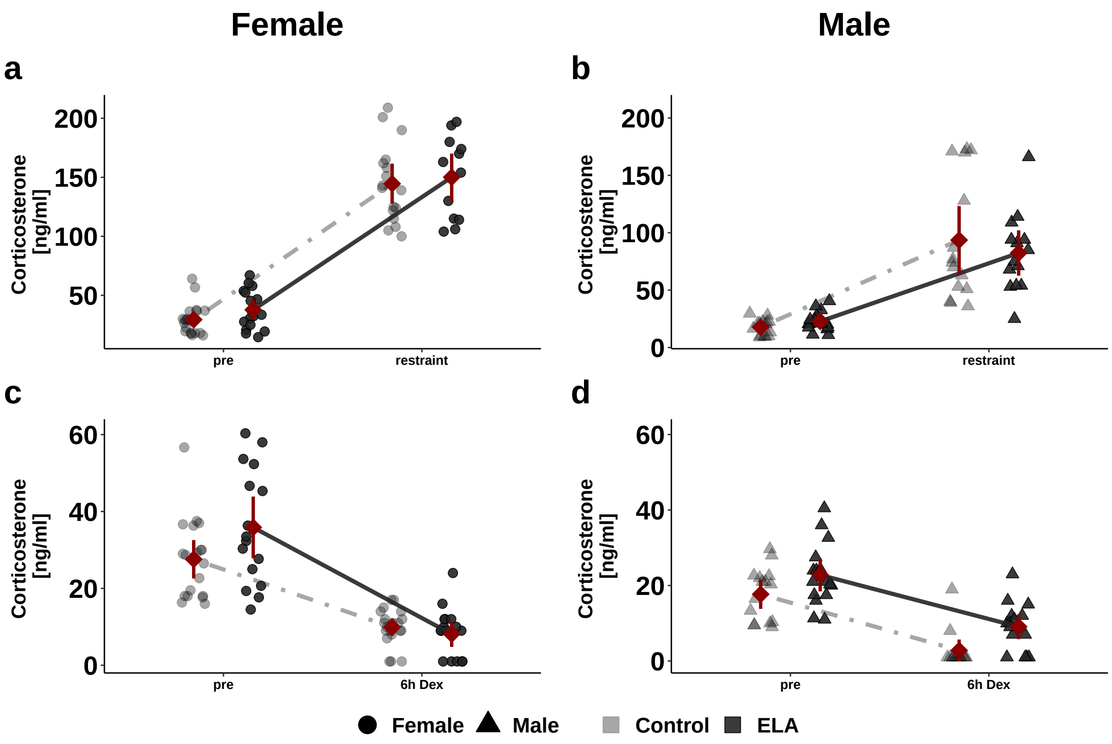

Supplementary Figure 2: **Stimulated Plasma Corticosterone Levels in Wild Type Females Fluctuate more than in Males.**
Individual animal data (grey scale) is shown alongside with the mean ± 95% confidence interval (red) to indicate statistical differences. Selected findings in the ANOVA are indicated in addition, while all descriptive statistics, model summaries and ANOVA outcomes for dexamethasone-suppressed and stress-induced corticosterone are provided in Tab. 1, Tab. 7, Tab. 8, Tab. 9 and Tab. 10. Acute responsiveness of the HPA-axis to 5 minutes of immobilisation stress leads to an increase in corticosterone ($p < 1^{-5}$) that is higher in females (**a**) than males (**b**, $p < 1^{-5}$). Dexamethasone induced suppression of endogenous corticosterone 6 hours after injection ($p < 1^{-5}$) is stronger in females (**c**) than males (**d**, $p < 1^{-5}$). No differences between wild type and *Fkbp5*-humanized mice were observed.

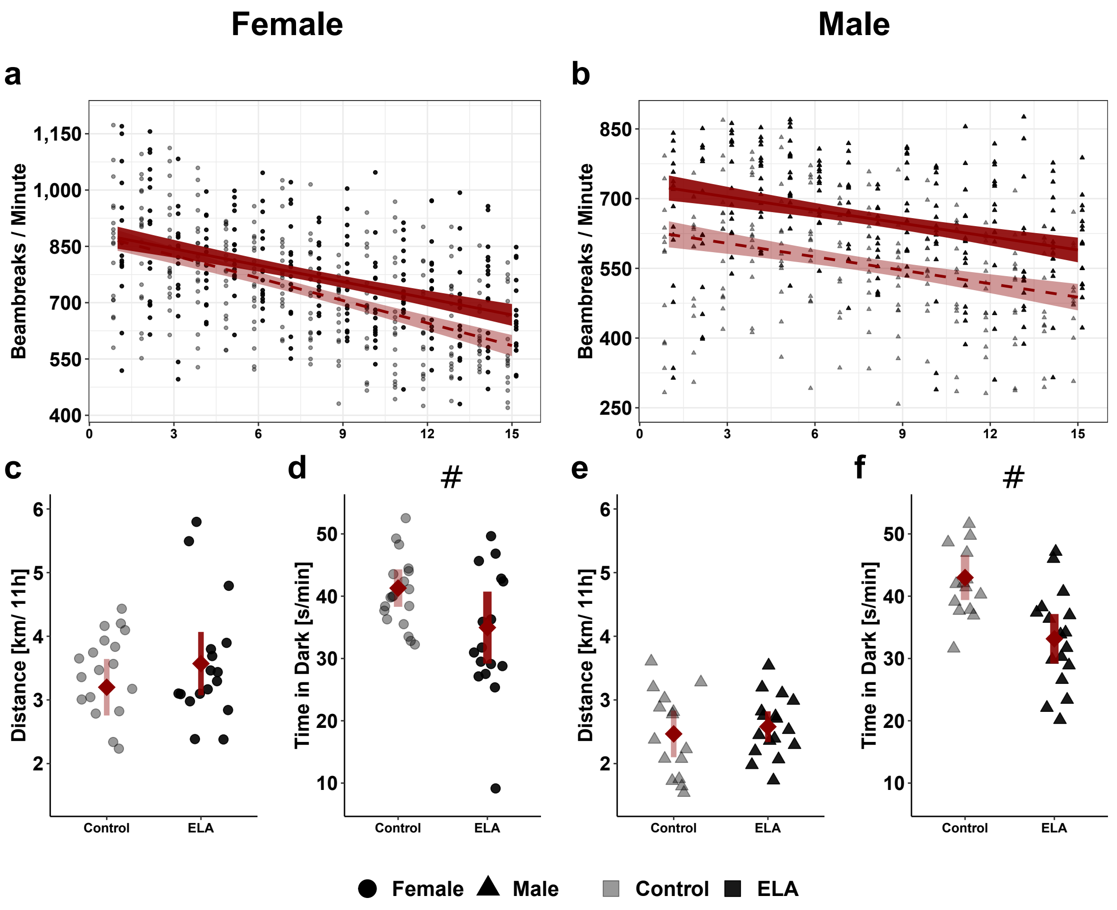

Supplementary Figure 3: **ELA Increases Exploration Behavior but not Overall Activity in Wild Type Mice**
Individual animal data is shown alongside with the mean ± 95% confidence interval (red) to indicate significant subgroup differences. Selected findings in the ANOVA are reported, while all descriptive statistics, model summaries and ANOVA results are provided in Tab. 11, Tab. 12 and Tab. 13 for the exploration activity, in Tab. 14, Tab. 15 and Tab. 16 for the nocturnal distance, and in Tab. 17, Tab. 18 and Tab. 19 for the Dark-Light-Test. Increased breaking of light beams during the first 15 minutes in a novel environment in females (**a**) vs. males (**b**, $p = 1^{-5}$) and with ELA compared to controls ($p < .05$). Females (**c**) run more distance during the night than males (**e**, $p < 1^{-5}$). Average time per minute females (**d**) and males (**f**) spent in the dark compartment of the arena decreases after ELA (#, $p < 1^{-5}$). This effect was visible in wild type and CG-allele carrying male and female mice.

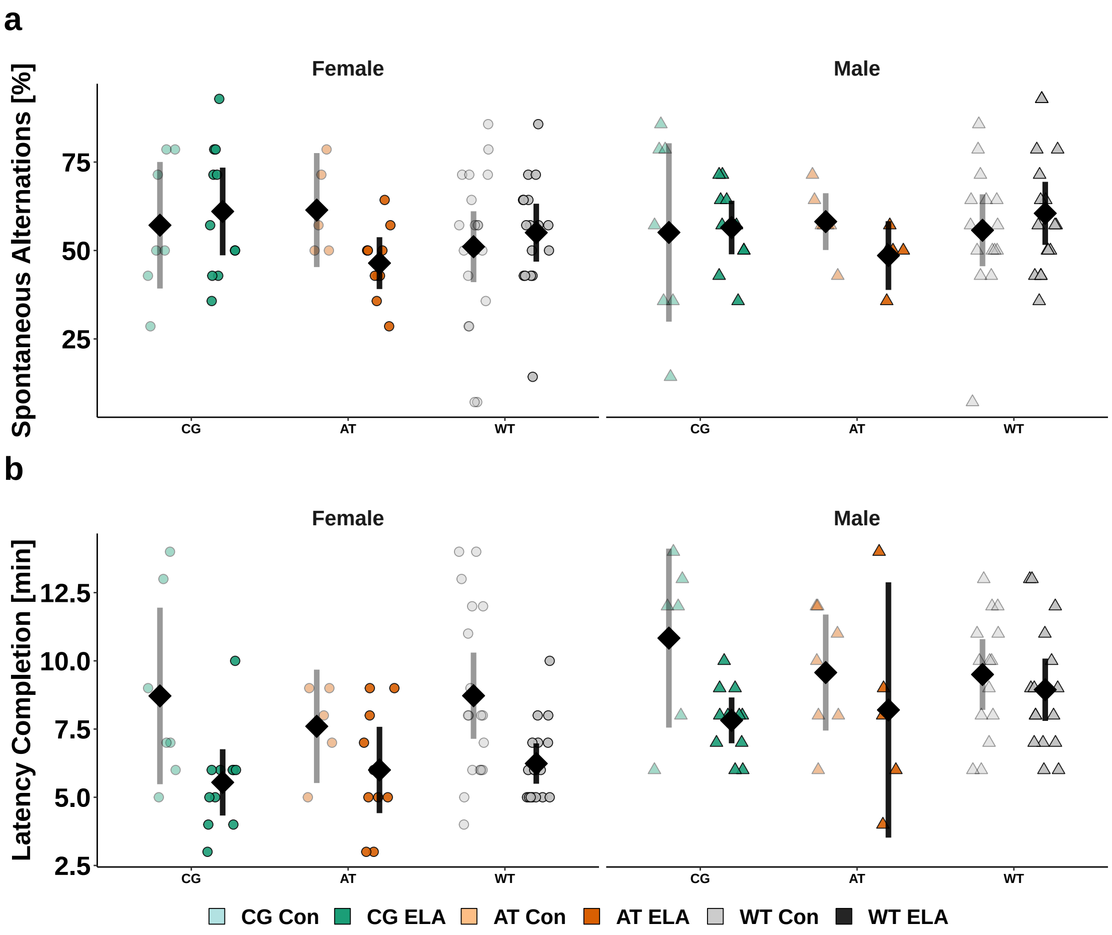

Supplementary Figure 4: **ELA Shortens the Time to Completion of the T-Maze Test in *Fkbp5*-Humanized and Wild Type Mice**
Individual animal data is shown alongside with the mean (black diamond) ± 95% confidence interval to indicate significant differences between subgroups. Descriptive statistics, model summaries and ANOVA results are provided in Tab. 20, Tab 21, and Tab. 22 for the spontaneous alternations and in Tab. 23, Tab. 24 and Tab. 25 for the latency of T-maze completion. The percentage of spontaneous switching between arms of the T-shaped arena is performed at chance level with no statistically significant effect of early life condition, *Fkbp5*-genotype or sex (**a**). The time [min] needed to complete the 15 trials differed between controls and ELA-exposed animals ($p < 1^{-5}$) and was further modulated by sex with females being faster than males ($p < 1^{-5}$, **b**).

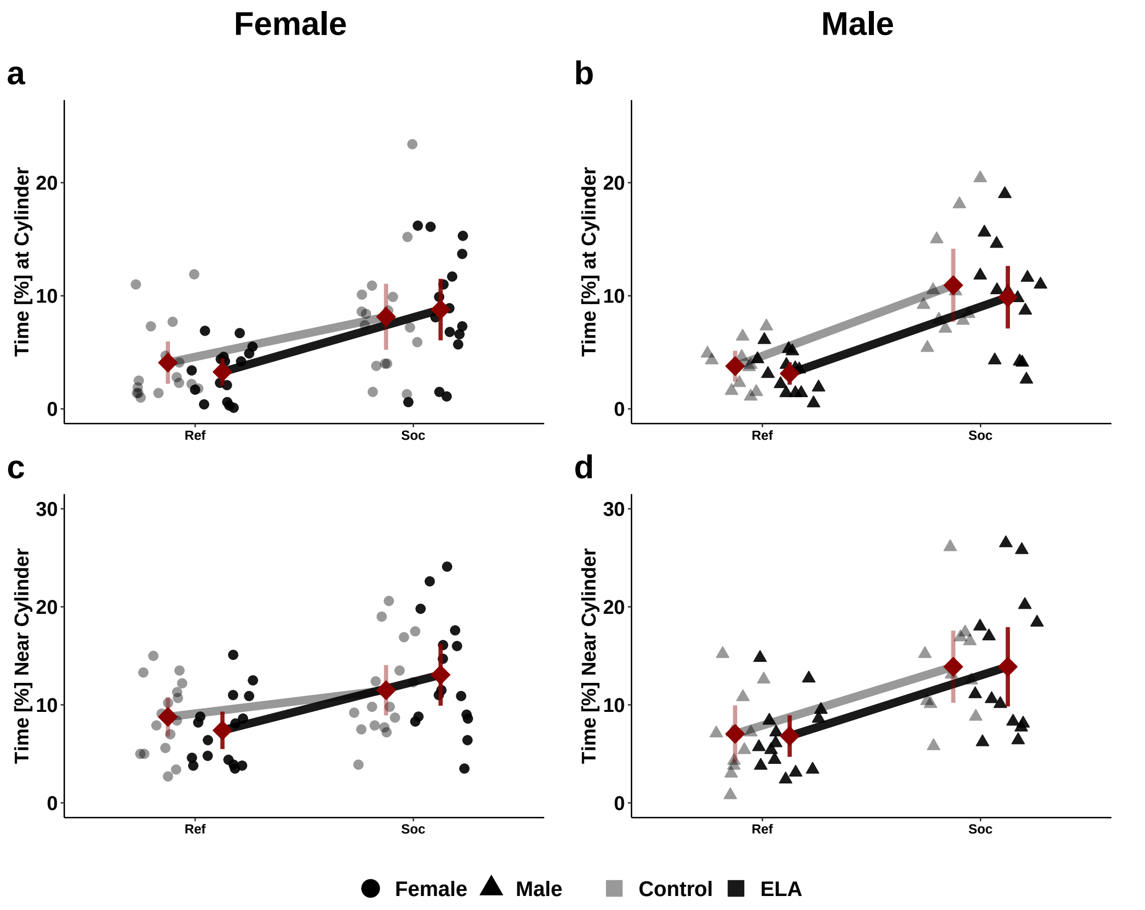

Supplementary Figure 5: **Wild Type Mice Maintain Social Preference Despite ELA**
Individual animal data is shown alongside with the mean ± 95% confidence interval (red) to indicate significant differences between subgroups. Descriptive statistics, model summaries and ANOVA results are provided for social interaction time (Tab. 26, Tab. 27, Tab. 28) and for the time in social distance (Tab. 29, Tab. 30, Tab. 31) are provided. Comparison of the percent of time females (**a**) and males (**b**) spent at the cylinder with (Soc) or without (Ref) an unfamiliar mouse. Social preference was seen in all subgroups except for female wild type controls. Relative time [%] spent in the donut-shaped area surrounding the cylinder shown for females (**c**) and males (**d**). Preference for the social side was seen in all subgroups except for female wild type controls.

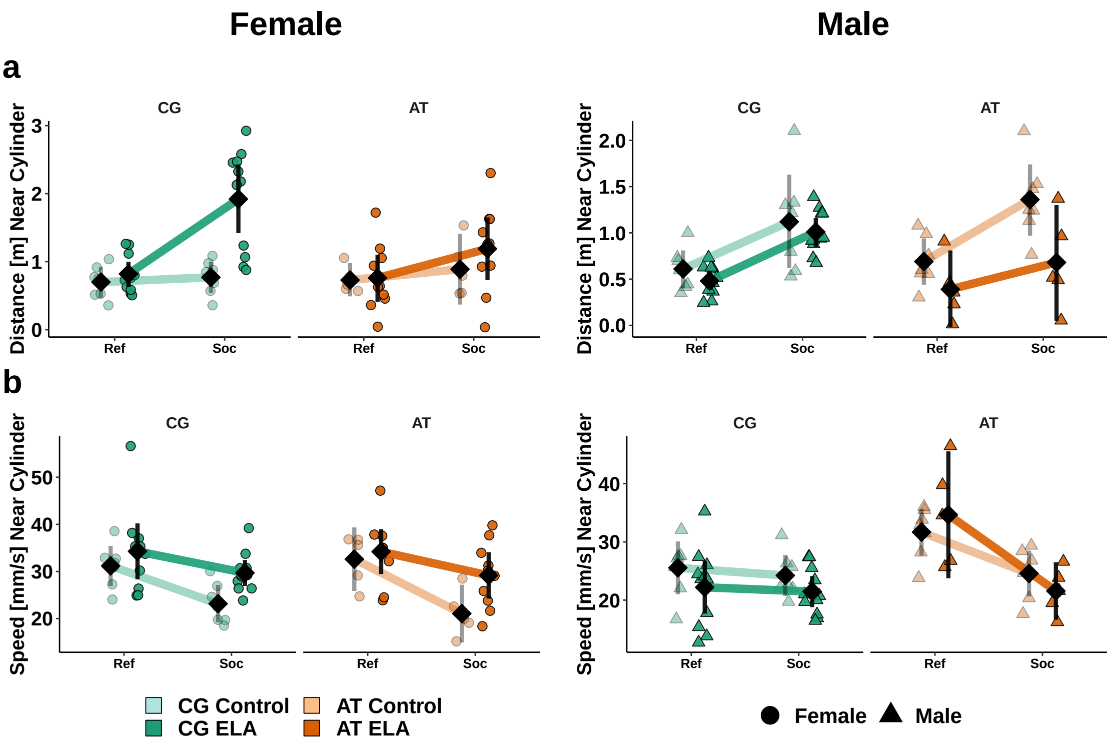

Supplementary Figure 6: **Activity of *Fkbp5*-Humanized Mice in Proximity to Unfamiliar Mice**

Individual animal data is shown alongside with the mean (black diamond) ± 95% confidence interval to indicate significant differences between subgroups. Descriptive statistics, model summaries and ANOVA results are provided in Tab. 32, Tab 33, and Tab. 34 for the moved distance and in Tab. 35, Tab. 36 and Tab. 37 for the speed. The distance [m] moved in the area surrounding the cylinder of the social chamber test with (Soc) or without (Ref) an unfamiliar stimulus mouse (**a**) was higher on the social side ($p < 1^{-5}$) in CG-allele carriers with ELA and male AT-allele carrying controls (ELA × Sex × Compartment p = .007, Strain × Compartment p = .08). Speed [mm/second] was lower in the area surrounding the cylinder of the social chamber test with (Soc) vs. without (Ref) an unfamiliar stimulus mouse (**b**, $py1^{-5}$) but not in the individual subgroups.

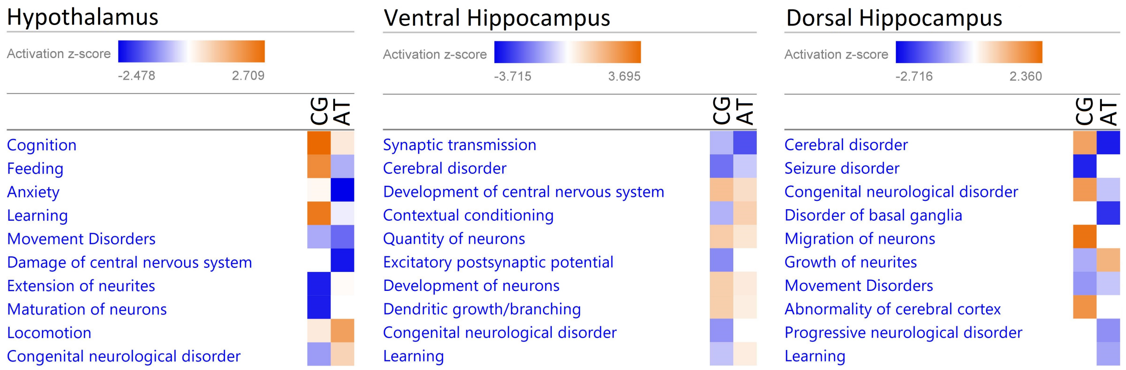

Supplementary Figure 7: **Comparison of ELA-Induced Changes in Neurological and Developmental Disease Pathways between *Fkbp5*-Genotypes** Clustered top 10 activation z-scores between CG-allele (left colum) and AT-allele (right colum) carriers in hypothalamus (**left**), ventral hippocampus (**middle**) and dorsal hippocampus (**right**).

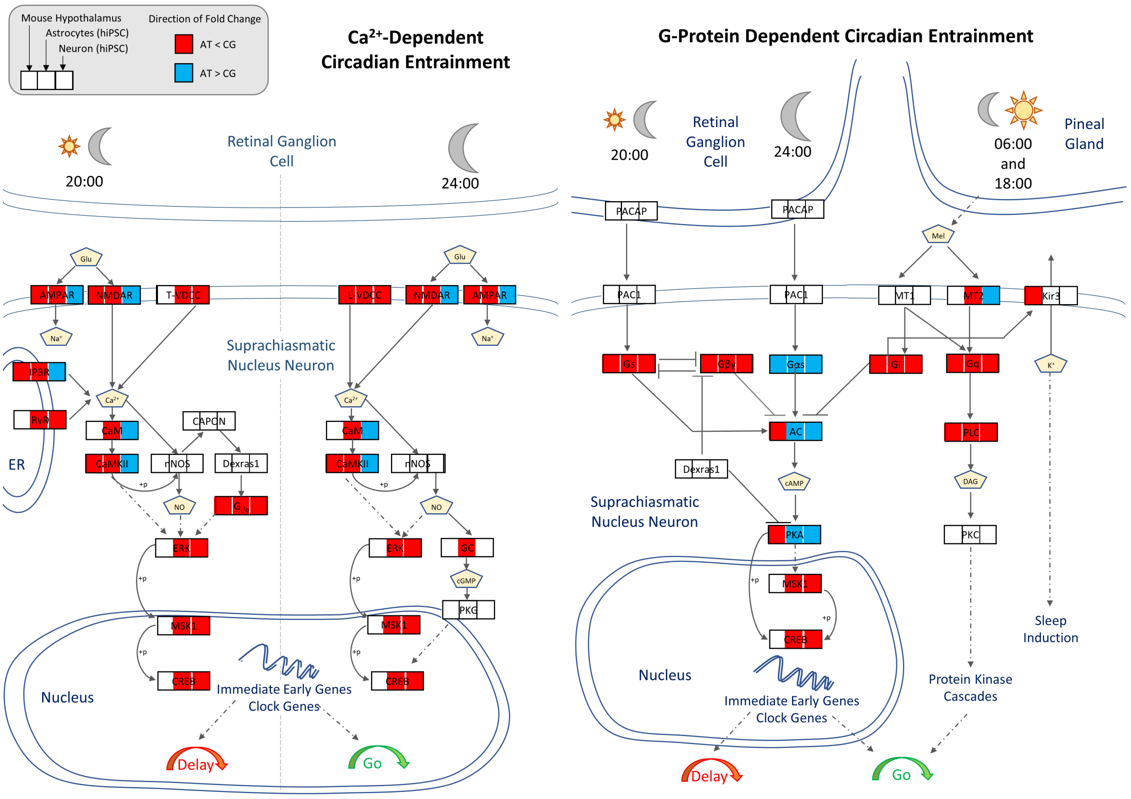

Supplementary Figure 8: **Differential Expression of Genes Related to Circadian Entrainment in *Fkbp5*-Mice and in hiPSC-Derived Astrocytes and Neurons of Human *FKBP5*-SNP Carriers**
Visualization of significant fold changes between AT- vs. CG-allele shown for mouse hypothalamus, hiPSC-derived astrocytes and neurons. The calcium-dependent and the G-protein dependent arm of the circadian entrainment KEGG pathway is shown on the left and right, respectively.

Supplementary Table 1: **Descriptive Corticosterone Concentration [ng/ml] in *Fkbp5*-Humanized and Wild Type Mice**

Strain	Sex	Group	Time	N	Mean	SD	95% CI low	95% CI up
AT	Female	Control	Morning	6	40.50	11.90	28.01	52.99
			Noon	6	99.50	55.30	41.46	157.54
			Evening	6	109.83	49.63	57.75	161.92
			restraint	5	135.20	28.49	99.82	170.58
			6h Dex	6	6.83	6.88	-0.39	14.06
		ELA	Morning	11	52.42	22.27	37.46	67.38
			Noon	10	78.40	33.56	54.39	102.41
			Evening	11	86.82	31.22	65.85	107.79
			restraint	11	180.00	52.19	144.94	215.06
			6h Dex	10	9.10	12.82	-0.07	18.27
AT	Male	Control	Morning	7	19.14	8.85	10.96	27.33
			Noon	7	50.43	24.73	27.56	73.30
			Evening	7	41.86	15.94	27.11	56.60
			restraint	7	103.71	62.87	45.57	161.86
			6h Dex	7	4.71	6.58	-1.37	10.80
		ELA	Morning	5	16.73	2.97	13.05	20.42
			Noon	5	48.40	18.04	26.01	70.79
			Evening	5	44.40	22.71	16.20	72.60
			restraint	4	73.75	21.27	39.91	107.59
			6h Dex	5	3.20	4.92	-2.91	9.31
CG	Female	Control	Morning	7	28.86	13.87	16.03	41.69
			Noon	7	35.43	13.48	22.96	47.89
			Evening	7	109.86	53.76	60.14	159.58
			restraint	7	147.14	77.04	75.89	218.40
			6h Dex	7	7.57	6.29	1.75	13.39
		ELA	Morning	11	37.18	18.60	24.69	49.68
			Noon	11	99.36	33.76	76.68	122.04
			Evening	11	73.82	32.58	51.93	95.71
			restraint	11	170.64	60.70	129.86	211.41
			6h Dex	11	5.55	9.20	-0.64	11.73
CG	Male	Control	Morning	7	12.29	2.89	9.61	14.96
			Noon	7	107.43	138.97	-21.10	235.96
			Evening	7	22.57	15.52	8.22	36.93
			restraint	7	97.29	28.77	70.67	123.90
			6h Dex	7	1.00	0.00	1.00	1.00
		ELA	Morning	12	17.64	7.17	13.08	22.20
			Noon	12	45.75	12.20	38.00	53.50
			Evening	10	31.40	19.13	17.72	45.08
			restraint	12	102.92	30.43	83.59	122.25
			6h Dex	12	2.83	4.61	-0.10	5.76
WT	Female	Control	Morning	20	29.39	12.97	23.32	35.46
			Noon	20	96.35	72.41	62.46	130.24
			Evening	19	137.05	81.53	97.76	176.35

			restraint	19	152.58	39.29	133.64	171.52
			6h Dex	20	9.90	4.73	7.68	12.12
		ELA	Morning	17	37.69	16.44	29.24	46.14
			Noon	16	79.81	45.35	55.65	103.98
			Evening	16	66.50	28.68	51.22	81.78
			restraint	16	168.44	49.57	142.02	194.85
			6h Dex	17	8.12	6.49	4.78	11.45
WT	Male	Control	Morning	15	17.69	7.01	13.81	21.57
			Noon	13	84.62	66.73	44.29	124.94
			Evening	13	82.77	47.74	53.92	111.62
			restraint	15	93.60	53.65	63.89	123.31
			6h Dex	14	2.79	5.03	-0.12	5.69
		ELA	Morning	16	22.79	8.19	18.43	27.15
			Noon	17	81.00	34.87	63.07	98.93
			Evening	17	31.88	18.55	22.34	41.42
			restraint	16	102.88	64.52	68.49	137.26
			6h Dex	17	11.59	12.00	5.42	17.76

Supplementary Table 2: **Model Summary Diurnal Corticosterone**

**Model Terms**	β	$\boldsymbol{SE}$	**Df**	**CI**$_{low}$	**CI**$_{up}$	$\boldsymbol{t}$	$\boldsymbol{p}$	$\boldsymbol{R^2_{part}}$
Intercept	12.29	15.57	235	-16.95	41.52	0.79	0.4308	0.4283
ELA	5.35	19.59	123	-31.61	42.32	0.27	0.7851	0.0502
AT	6.86	22.02	123	-34.69	48.4	0.31	0.756	0.0266
WT	5.4	18.85	123	-30.17	40.98	0.29	0.7749	0.0224
Female	16.57	22.02	123	-24.97	58.12	0.75	0.4531	0.0223
Noon	95.14	21.09	235	55.53	134.76	4.51	0	0.0187
Evening	10.29	21.09	235	-29.33	49.9	0.49	0.6263	0.0186
ELA:AT	-7.76	31.07	123	-66.39	50.87	-0.25	0.8031	0.0163
ELA:WT	-0.08	24.55	123	-46.41	46.24	0	0.9973	0.0159
ELA:Female	2.97	27.94	123	-49.74	55.68	0.11	0.9155	0.0144
AT:Female	4.79	31.78	123	-55.18	64.75	0.15	0.8805	0.0118
WT:Female	-4.87	26.13	123	-54.17	44.43	-0.19	0.8525	0.0115
ELA:Noon	-67.03	26.54	235	-116.88	-17.19	-2.53	0.0122	0.008
ELA:Evening	3.4	27.06	235	-47.42	54.23	0.13	0.9	0.0079
AT:Noon	-63.86	29.83	235	-119.88	-7.84	-2.14	0.0333	0.0066
WT:Noon	-28.45	25.88	235	-77.05	20.15	-1.1	0.2727	0.004
AT:Evening	12.43	29.83	235	-43.59	68.45	0.42	0.6773	0.0031
WT:Evening	54.62	25.88	235	6.02	103.23	2.11	0.0359	0.0016
Female:Noon	-88.57	29.83	235	-144.59	-32.55	-2.97	0.0033	0.0016
Female:Evening	70.71	29.83	235	14.69	126.74	2.37	0.0186	0.0008
ELA:AT:Female	11.36	42.42	123	-68.67	91.4	0.27	0.7892	0.0007
ELA:WT:Female	0.05	34.41	123	-64.88	64.98	0	0.9988	0.0006
ELA:AT:Noon	67.41	42.1	235	-11.65	146.47	1.6	0.1107	0.0005
ELA:WT:Noon	58.38	33.45	235	-4.43	121.2	1.75	0.0822	0.0003
ELA:AT:Evening	1.55	42.43	235	-78.13	81.23	0.04	0.9709	0.0002
ELA:WT:Evening	-59.39	33.86	235	-122.98	4.2	-1.75	0.0808	0.0002
ELA:Female:Noon	122.64	37.85	235	51.56	193.72	3.24	0.0014	0.0002
ELA:Female:Evening	-47.77	38.22	235	-119.54	24	-1.25	0.2126	0.0002
AT:Female:Noon	116.29	43.06	235	35.43	197.15	2.7	0.0074	0.0001
WT:Female:Noon	88.84	35.64	235	21.9	155.78	2.49	0.0134	0.0001
AT:Female:Evening	-24.1	43.06	235	-104.96	56.76	-0.56	0.5763	0
WT:Female:Evening	-27.91	35.71	235	-94.96	39.15	-0.78	0.4352	0
ELA:AT:Female:Noon	-155.97	57.6	235	-264.14	-47.8	-2.71	0.0073	0
ELA:WT:Female:Noon	-138.62	46.82	235	-226.54	-50.7	-2.96	0.0034	0
ELA:AT:Female:Evening	7.88	57.71	235	-100.5	116.26	0.14	0.8916	0
ELA:WT:Female:Evening	24.51	47.16	235	-64.06	113.07	0.52	0.6038	0

Model formula:
lme(~ Group*Strain*Sex*Time, random = ~ 1|ID/Time)
$R^2_{marg} = 42.57\%$, $R^2_{cond} = 95.17\%$
Abbreviations: β = β coefficient estimate, SE = standard error, Df = degrees of freedom, CI = 95% confidence interval, part = partial, group = early life condition group, marg = marginalized, cond = conditioned,

**Hypothalamus**	1500009L16Rik	Afmid	Chrna7	Gjb1	Irf3	Lonp2	Morf4l2	Pkmyt1	Slc35a5	Tigd5
Fkbp5	0.76	0.76	-0.33	0.38	0.77	-0.76	0.79	-0.31	0.76	0.76
Corticosterone Morning	-0.11	-0.43	0.76	-0.37	-0.19	0.55	-0.24	0.76	-0.35	-0.23
Dark Compartment	-0.06	0.05	-0.28	0.76	-0.12	-0.12	0.02	-0.23	0.03	-0.01

**Ventral Hippocampus**	Acads	Cyba	Dnaaf3	Eng	Fam166b	Islr	Lrrc7	Odf3b	Perp	Sertad3
Fkbp5	0.91	-0.88	0.86	-0.87	0.91	0.85	0.86	0.86	0.85	0.86

**Dorsal Hippocampus**	Becn1	Bmp6	Btd	Htr1a	Itprid2	Lgi4	Nanp	Ndrg4	St8sia5	Stxbp5
Fkbp5	-0.88	0.86	-0.88	0.87	0.92	0.87	0.86	0.88	0.89	0.87

Supplementary Figure 9: **Top 10 Correlated DEGs with *Fkbp5*, *in vivo* and *ex vivo* Readouts**

Significant positive and negative correlations per brain region are highlighted in red and blue, respectively.

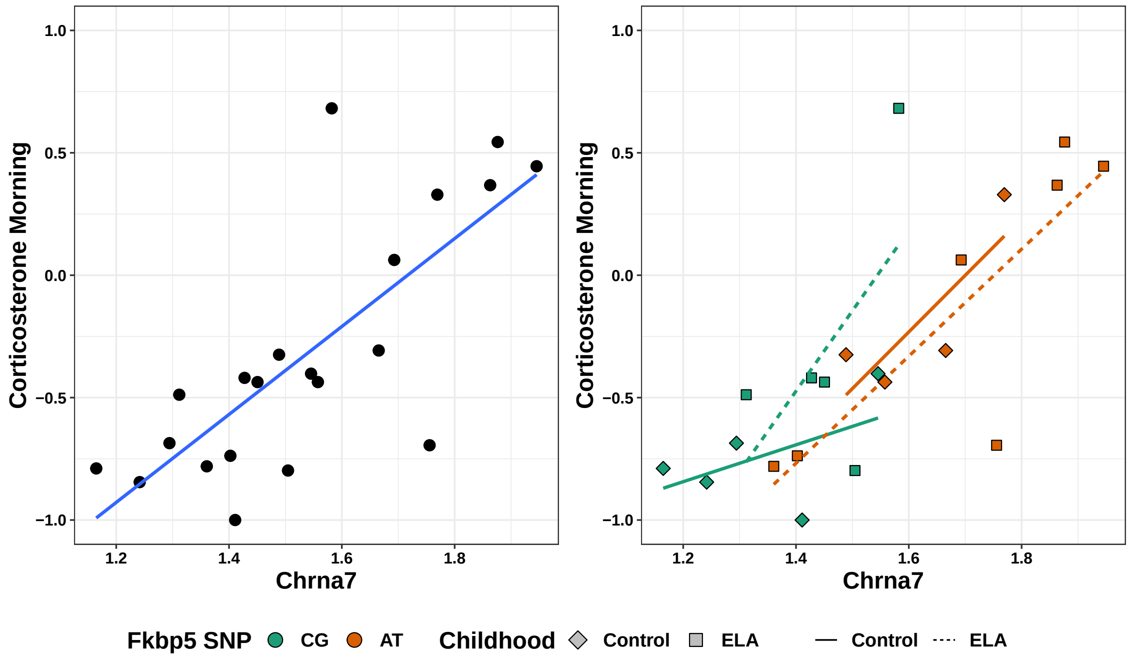

Supplementary Figure 10: **Regression of Morning Corticosterone Levels and *Chrna7***

Overall linear association (**left**) and regressions in the subgroups defined by *Fkbp5* SNP and early life experience (**right**) are shown for the scaled morning corticosterone and log-transformed expression levels.

Supplementary Table 3: **ANOVA Diurnal Corticosterone**

**Model Terms**	**Df**$_{num}$	***F***	***GES***	***p***	$\epsilon^2_{partial}$			**Cohen's F**$_{partial}$		
					**Estimate**	**CI**$_{low}$	**CI**$_{up}$	**Estimate**	**CI**$_{low}$	**CI**$_{up}$
**Group**	1	7.51	0.0254	**0.00715**	0.0545	0.0024	0.1544	0.2589	0.0696	0.4466
Strain	2	2.05	0.014	0.13358	0.0181	0	0.082	0.1913	0	0.3608
**Sex**	1	31.24	0.0977	**<0.00001**	0.2111	0.0924	0.3373	0.5281	0.3294	0.7248
Group:Strain	2	2.05	0.014	0.13372	0.0181	0	0.082	0.1913	0	0.3607
Group:Sex	1	0.07	0.0002	0.79438	-0.0083	0	0	0.0247	0	0.1962
Strain:Sex	2	1.38	0.0095	0.25462	0.0067	0	0.0514	0.1573	0	0.3233
Group:Strain:Sex	2	1.35	0.0093	0.26399	0.0061	0	0.0491	0.1551	0	0.3209
**Time**	2	59.04	0.2439	**<0.00001**	0.3393	0.2424	0.4252	0.726	0.5746	0.8698
**Group:Time**	2	9.98	0.0517	**0.00022**	0.0736	0.0183	0.1431	0.2986	0.1539	0.4259
Strain:Time	3	1.13	0.0122	0.34005	0.0023	0	0.0012	0.1421	0	0.2366
**Sex:Time**	2	7.8	0.0409	**0.00121**	0.0567	0.0094	0.1207	0.2638	0.1176	0.3898
Group:Strain:Time	3	1.93	0.0207	0.11891	0.0161	0	0.0457	0.1859	0	0.2887
**Group:Sex:Time**	2	3.58	0.0192	**0.03795**	0.0223	0	0.0691	0.1787	0	0.3008
Strain:Sex:Time	3	0.68	0.0073	0.58235	-0.0057	0	0	0.1099	0	0.1941
**Group:Strain:Sex:Time**	3	3.48	0.0366	**0.01389**	0.0417	0	0.09	0.2492	0.066	0.3596

Model formula:
lme(~ Group*Strain*Sex*Time, random = ~ 1|ID/Time)
$\text{Df}_{denBetween} = 112$, $MSE_{between} = 2072$, $\text{Df}_{denWithin} = 186$, $MSE_{within} = 1965$
Abbreviations: group = early life condition group, num = numerator, den = denominator, Df = degrees of freedom, *MSE* = mean standard error, *GES* = generalized η^2, CI = 95% confidence interval

Supplementary Table 4: **Descriptive Adrenal Weight [mg]**

**Strain**	**Sex**	**Group**	**N**	**Mean**	SD	**95% CI low**	**95% CI up**
CG	Female	Control	8	4.66	1.52	3.39	5.94
		ELA	13	6.18	1.04	5.55	6.80
	Male	Control	8	2.93	0.70	2.34	3.51
		ELA	11	2.17	0.65	1.74	2.61
AT	Female	Control	6	6.07	1.00	5.02	7.11
		ELA	10	6.11	1.26	5.21	7.01
	Male	Control	8	2.49	0.93	1.71	3.27
		ELA	5	2.62	0.56	1.92	3.32
WT	Female	Control	18	6.12	0.91	5.67	6.57
		ELA	14	6.59	1.08	5.97	7.21
	Male	Control	11	2.38	0.51	2.04	2.73
		ELA	14	2.09	0.69	1.69	2.49

Supplementary Table 5: **Model Summary Adrenal Weight**

**Model Terms**	β	***SE***	***t***	***p***	**CI**$_{low}$	**CI**$_{up}$
Intercept	2.92	0.33	8.77	¡0.00001	2.26	3.59
ELA	-0.75	0.44	-1.72	0.08874	-1.62	0.12
AT	-0.44	0.47	-0.93	0.35545	-1.37	0.5
WT	-0.54	0.44	-1.24	0.21767	-1.41	0.32
Female	1.74	0.47	3.68	0.00035	0.8	2.67
ELA:AT	0.88	0.69	1.28	0.20467	-0.49	2.26
ELA:WT	0.46	0.58	0.79	0.43033	-0.69	1.61
ELA:Female	2.27	0.61	3.72	0.00031	1.06	3.47
AT:Female	1.84	0.69	2.65	0.0091	0.47	3.22
WT:Female	2	0.59	3.36	0.00105	0.82	3.17
ELA:AT:Female	-2.36	0.95	-2.49	0.01435	-4.23	-0.48
ELA:WT:Female	-1.5	0.79	-1.89	0.06157	-3.07	0.07

Model formula:
lm(~ Group*Strain*Sex)
$R^2 = 81\%$, $R^2_{adj} = 80\%$, $RMSE = 0.9$, $\mathrm{Df}_{num} = 12$, $\mathrm{Df}_{den} = 114$, $\mathrm{F}_{mod} = 45.4$
Abbreviations: β = β coefficient estimate, *SE* = standard error, CI = 95% confidence interval, group = early life condition group, adj = adjusted, *RMSE* = root mean square error (sigma), Df = degrees of freedom, num = numerator, den = denominator, mod = model

Supplementary Table 6: **ANOVA Adrenal Weight**

**Model Terms**	**Sum of Squares**	**Df**	$\boldsymbol{F}$	$\boldsymbol{p}$	$\eta^2_{partial}$ **Estimate**	$\mathbf{CI}_{low}$	$\mathbf{CI}_{up}$	$\epsilon^2_{partial}$ **Estimate**	$\mathbf{CI}_{low}$	$\mathbf{CI}_{up}$
Group	1.4	1	1.57	0.2121	0.01	0	0.08	0	0	0.05
Strain	2.51	2	1.41	0.24783	0.04	0	0.13	0.03	0	0.1
**Sex**	408.75	1	459.62	<**0.00001**	0.8	0.74	0.85	0.8	0.74	0.85
Group:Strain	0.77	2	0.43	0.64915	0.01	0	0.05	-0.01	0	0
**Group:Sex**	8.35	1	9.39	**0.00273**	0.07	0.01	0.17	0.06	0	0.16
**Strain:Sex**	7.37	2	4.15	**0.01829**	0.07	0	0.16	0.05	0	0.14
**Group:Strain:Sex**	6.01	2	3.38	**0.03757**	0.06	0	0.15	0.04	0	0.12

Model formula: lm(~ Group*Strain*Sex)

Sum of $\text{Squares}_{residual} = 101$, $\text{Df}_{residual} = 114$

Abbreviations: group = early life condition group, Df = degrees of freedom, CI = 95% confidence interval

Supplementary Table 7: **Model Summary Dexamethasone Suppression**

**Model Terms**	β	$\boldsymbol{SE}$	**Df**	$\textbf{CI}_{low}$	$\textbf{CI}_{up}$	$\boldsymbol{t}$	$\boldsymbol{p}$	$\boldsymbol{R}^2_{part}$
Intercept	12.29	4.03	123	4.68	19.89	3.05	0.0028	0.6342
ELA	5.35	5.07	123	-4.22	14.92	1.06	0.2929	0.034
AT	6.86	5.7	123	-3.9	17.61	1.2	0.2309	0.0161
WT	5.4	4.88	123	-3.81	14.61	1.11	0.2701	0.0064
Female	16.57	5.7	123	5.82	27.33	2.91	0.0043	0.006
Dex	-11.29	5.66	120	-21.97	-0.6	-1.99	0.0484	0.0051
ELA:AT	-7.76	8.04	123	-22.94	7.42	-0.97	0.3361	0.0046
ELA:WT	-0.26	6.35	123	-12.25	11.74	-0.04	0.9675	0.0039
ELA:Female	2.97	7.23	123	-10.68	16.62	0.41	0.6817	0.0031
AT:Female	4.79	8.22	123	-10.74	20.31	0.58	0.5615	0.0027
WT:Female	-4.87	6.76	123	-17.63	7.9	-0.72	0.4727	0.0026
ELA:Dex	-3.52	7.12	120	-16.97	9.93	-0.49	0.622	0.0022
AT:Dex	-3.14	8	120	-18.26	11.97	-0.39	0.6952	0.0019
WT:Dex	-3.63	6.89	120	-16.64	9.39	-0.53	0.5998	0.0014
Female:Dex	-10	8	120	-25.12	5.12	-1.25	0.2139	0.0013
ELA:AT:Female	9.44	10.97	123	-11.28	30.16	0.86	0.3914	0.0013
ELA:WT:Female	0.23	8.9	123	-16.58	17.04	0.03	0.9795	0.0011
ELA:AT:Dex	4.42	11.29	120	-16.92	25.75	0.39	0.6965	0.001
ELA:WT:Dex	7.24	8.93	120	-9.64	24.11	0.81	0.4195	0.0007
ELA:Female:Dex	-6.83	10.15	120	-26.01	12.35	-0.67	0.5024	0.0006
AT:Female:Dex	-9.24	11.55	120	-31.06	12.58	-0.8	0.4254	0.0006
WT:Female:Dex	5.42	9.53	120	-12.57	23.41	0.57	0.5704	0.0001
ELA:AT:Female:Dex	-1.75	15.45	120	-30.93	27.44	-0.11	0.9102	0
ELA:WT:Female:Dex	-6.96	12.51	120	-30.6	16.67	-0.56	0.579	0

Model formula:
lme(~ Group*Strain*Sex*SampleType, random = ~ 1|ID/SampleType)
$R^2_{marg} = 63.25\%$, $R^2_{cond} = 98.86\%$
Abbreviations: β = β coefficient estimate, SE = standard error, Df = degrees of freedom, CI = 95% confidence interval, part = partial, group = early life condition group, marg = marginalized, cond = conditioned,

Supplementary Table 8: **ANOVA Dexamethasone Suppression Test**

**Model Terms**	**Df**$_{num}$	$\boldsymbol{F}$	***GES***	$\boldsymbol{p}$	$\epsilon^2_{partial}$			**Cohen's F**$_{partial}$		
					**Estimate**	**CI**$_{low}$	**CI**$_{up}$	**Estimate**	**CI**$_{low}$	**CI**$_{up}$
**Group**	1	8.48	0.0345	**0.00429**	0.0582	0.0043	0.1559	0.2658	0.083	0.4473
**Strain**	2	4.16	0.0339	**0.01793**	0.0492	0	0.1346	0.2633	0.0402	0.4325
**Sex**	1	58.99	0.1992	<**0.00001**	0.324	0.1949	0.4422	0.7011	0.5003	0.8996
Group:Strain	2	0.54	0.0045	0.58400	-0.0076	0	0	0.0949	0	0.2455
Group:Sex	1	0.09	0.0004	0.76401	-0.0076	0	0	0.0275	0	0.1964
Strain:Sex	2	2.75	0.0227	*0.06807*	0.0279	0	0.099	0.214	0	0.3799
Group:Strain:Sex	2	1.05	0.0088	0.35248	0.0008	0	0.0116	0.1324	0	0.2903
**SampleType**	1	276.49	0.5323	<**0.00001**	0.6948	0.6082	0.7578	1.5179	1.254	1.7787
Group:SampleType	1	2.38	0.0097	0.12583	0.0112	0	0.0755	0.1407	0	0.3202
Strain:SampleType	2	1.86	0.0151	0.15992	0.0139	0	0.0701	0.1761	0	0.339
**Sex:SampleType**	1	31.42	0.1145	<**0.00001**	0.2009	0.088	0.3227	0.5117	0.3205	0.701
Group:Strain:SampleType	2	0.36	0.003	0.69861	-0.0106	0	0	0.0774	0	0.2222
**Group:Sex:SampleType**	1	4.26	0.0172	**0.04108**	0.0263	0	0.1058	0.1885	0	0.3686
Strain:Sex:SampleType	2	1.15	0.0094	0.32064	0.0024	0	0.0294	0.1383	0	0.297
Group:Strain:Sex:SampleType	2	0.4	0.0033	0.66930	-0.0099	0	0	0.0819	0	0.2285

Model formula:
lme(~ Group*Strain*Sex*SampleType, random = ~ 1|ID/SampleType)
$\mathrm{Df}_{denBetween} = 120$, $MSE_{between} = 108$, $\mathrm{Df}_{denWithin} = 120$, $MSE_{within} = 106$
Abbreviations: group = early life condition group, num = numerator, den = denominator, Df = degrees of freedom, *MSE* = mean standard error, *GES* = generalized η^2, CI = 95% confidence interval

Supplementary Table 9: **Model Summary Restraint Stress Corticosterone**

**Model Terms**	β	$\boldsymbol{SE}$	**Df**	**CI**$_{low}$	**CI**$_{up}$	$\boldsymbol{t}$	$\boldsymbol{p}$	$\boldsymbol{R^2_{part}}$
Intercept	12.29	14.04	122	-14.21	38.78	0.88	0.3832	0.7238
ELA	5.35	17.66	122	-27.98	38.69	0.3	0.7623	0.0708
AT	6.86	19.85	122	-30.61	44.33	0.35	0.7304	0.0058
WT	5.4	17	122	-26.68	37.49	0.32	0.7511	0.0031
Female	16.57	19.85	122	-20.9	54.04	0.83	0.4055	0.0029
Restraint	85	19.48	118	48.22	121.78	4.36	0	0.002
ELA:AT	-7.76	28.01	122	-60.64	45.11	-0.28	0.7822	0.0013
ELA:WT	-0.25	22.14	122	-42.04	41.54	-0.01	0.991	0.0007
ELA:Female	2.97	25.19	122	-44.57	50.51	0.12	0.9063	0.0007
AT:Female	4.79	28.65	122	-49.29	58.87	0.17	0.8676	0.0006
WT:Female	-4.87	23.56	122	-49.33	39.6	-0.21	0.8366	0.0005
ELA:Restraint	0.28	24.51	118	-46	46.56	0.01	0.991	0.0004
AT:Restraint	-0.43	27.55	118	-52.44	51.58	-0.02	0.9876	0.0004
WT:Restraint	-9.09	23.59	118	-53.63	35.45	-0.39	0.7007	0.0003
Female:Restraint	33.29	27.55	118	-18.73	85.3	1.21	0.2293	0.0003
ELA:AT:Female	9.44	38.24	122	-62.74	81.62	0.25	0.8055	0.0003
ELA:WT:Female	0.22	31.03	122	-58.34	58.78	0.01	0.9943	0.0002
ELA:AT:Restraint	-27.88	39.75	118	-102.93	47.18	-0.7	0.4845	0.0001
ELA:WT:Restraint	3.89	30.72	118	-54.11	61.9	0.13	0.8993	0.0001
ELA:Female:Restraint	14.89	34.95	118	-51.1	80.88	0.43	0.6708	0.0001
AT:Female:Restraint	-23.17	40.33	118	-99.32	52.98	-0.57	0.5667	0
WT:Female:Restraint	13.98	32.75	118	-47.84	75.81	0.43	0.6701	0
ELA:AT:Female:Restraint	47.52	54.14	118	-54.7	149.74	0.88	0.3819	0
ELA:WT:Female:Restraint	-11.53	43.15	118	-93.01	69.95	-0.27	0.7897	0

Model formula:
lme(~ Group*Strain*Sex*SampleType, random = ~ 1|ID/SampleType)
$R^2_{marg} = 72.52\%$, $R^2_{cond} = 98.61\%$
Abbreviations: $\beta = \beta$ coefficient estimate, SE = standard error, Df = degrees of freedom, CI = 95% confidence interval, part = partial, group = early life condition group, marg = marginalized, cond = conditioned,

Supplementary Table 10: **ANOVA Restraint Stress Corticosterone**

**Model Terms**	**Df**$_{num}$	$\boldsymbol{F}$	$\boldsymbol{GES}$	$\boldsymbol{p}$	$\epsilon^2_{partial}$			**Cohen's F**$_{partial}$		
					**Estimate**	**CI**$_{low}$	**CI**$_{up}$	**Estimate**	**CI**$_{low}$	**CI**$_{up}$
**Group**	1	4.27	0.0184	**0.04101**	0.0267	0	0.1075	0.1902	0	0.3719
Strain	2	0.15	0.0013	0.86509	-0.0145	0	0	0.0496	0	0.1753
**Sex**	1	70.59	0.2369	**<0.00001**	0.369	0.2377	0.484	0.7735	0.5666	0.9778
Group:Strain	2	0.02	0.0002	0.98166	-0.0166	0	0	0.0177	0	0
Group:Sex	1	2.04	0.0089	0.15560	0.0087	0	0.0699	0.1316	0	0.3125
Strain:Sex	2	0.32	0.0028	0.72473	-0.0114	0	0	0.074	0	0.2182
Group:Strain:Sex	2	1.07	0.0093	0.34799	0.0011	0	0.0156	0.1344	0	0.2937
**SampleType**	1	516.69	0.6781	**<0.00001**	0.8125	0.7558	0.8522	2.0925	1.7691	2.413
Group:SampleType	1	0.5	0.002	0.48276	-0.0043	0	0	0.0648	0	0.2447
Strain:SampleType	2	0.37	0.003	0.69132	-0.0106	0	0	0.0792	0	0.2257
**Sex:SampleType**	1	24.08	0.0894	**<0.00001**	0.1624	0.0591	0.2831	0.4517	0.2614	0.6402
Group:Strain:SampleType	2	0.01	0.0001	0.99149	-0.0168	0	0	0.012	0	0
Group:Sex:SampleType	1	0.89	0.0036	0.34817	-0.0009	0	0	0.0867	0	0.2672
Strain:Sex:SampleType	2	0.08	0.0007	0.91888	-0.0155	0	0	0.0379	0	0.1443
Group:Strain:Sex:SampleType	2	0.76	0.0061	0.47092	-0.0041	0	0	0.1133	0	0.2692

Model formula:
lme(~ Group*Strain*Sex*SampleType, random = ~ 1|ID/SampleType)
$Df_{denBetween} = 118$, $MSE_{between} = 1454$, $Df_{denWithin} = 118$, $MSE_{within} = 1348$
Abbreviations: group = early life condition group, num = numerator, den = denominator, Df = degrees of freedom, MSE = mean standard error, GES = generalized η^2, CI = 95% confidence interval

Supplementary Table 11: **Descriptive Beambreaks Novel Environment *Fkbp5*-Humanized and Wild Type Mice**

Strain	Sex	Group	Minute	N	Mean	SD	95% CI low	95% CI up
CG	Female	Control	1	7	930.43	165.37	777.49	1083.37
			2	7	883.71	113.22	779.00	988.43
			5	7	740.57	129.63	620.68	860.46
			10	7	680.57	159.12	533.41	827.74
			15	7	640.00	135.22	514.94	765.06
		ELA	1	11	933.73	165.27	822.70	1044.76
			2	11	917.55	133.53	827.84	1007.25
			5	11	826.27	126.28	741.44	911.11
			10	11	755.27	146.47	656.88	853.67
			15	11	713.73	151.06	612.25	815.21
	Male	Control	1	7	584.86	186.19	412.66	757.05
			2	7	636.29	217.71	434.94	837.63
			5	7	598.71	68.18	535.66	661.77
			10	7	573.71	52.56	525.11	622.32
			15	7	442.14	160.16	294.01	590.27
		ELA	1	11	640.45	206.55	501.69	779.22
			2	11	714.09	193.50	584.10	844.08
			5	11	635.36	182.28	512.91	757.82
			10	11	590.00	155.13	485.78	694.22
			15	11	583.27	182.96	460.36	706.18
AT	Female	Control	1	4	865.50	175.12	586.85	1144.15
			2	4	825.25	41.77	758.78	891.72
			5	4	808.50	117.71	621.20	995.80
			10	4	686.25	198.54	370.33	1002.17
			15	4	707.50	210.20	373.02	1041.98
		ELA	1	10	817.10	263.28	628.76	1005.44
			2	10	870.10	195.41	730.31	1009.89
			5	10	708.70	123.42	620.41	796.99
			10	10	650.50	166.73	531.23	769.77
			15	10	649.60	188.51	514.75	784.45
	Male	Control	1	7	631.14	188.82	456.52	805.77
			2	7	691.86	87.43	611.00	772.72
			5	7	705.86	112.21	602.08	809.63
			10	7	547.00	135.95	421.26	672.74
			15	7	472.86	101.99	378.53	567.19
		ELA	1	5	457.40	157.74	261.54	653.26
			2	5	602.60	165.87	396.65	808.55
			5	5	631.00	112.92	490.79	771.21
			10	5	605.20	155.49	412.14	798.26
			15	5	532.60	84.01	428.28	636.92
	Female	Control	1	20	840.05	215.85	739.03	941.07
			2	20	835.15	184.61	748.75	921.55
			5	20	780.50	113.82	727.23	833.77

		10	20	667.20	175.43	585.10	749.30
		15	20	564.35	137.49	500.00	628.70
	ELA	1	17	856.18	243.75	730.85	981.50
		2	17	888.71	173.93	799.28	978.13
		5	17	866.12	160.29	783.70	948.53
		10	17	706.12	127.78	640.42	771.81
		15	17	660.12	105.66	605.79	714.44
Male	Control	1	15	613.13	228.94	486.35	739.92
		2	15	574.73	130.75	502.33	647.14
		5	15	602.00	127.92	531.16	672.84
		10	15	563.13	95.31	510.35	615.91
		15	15	436.40	149.18	353.79	519.01
	ELA	1	17	664.59	171.33	576.50	752.68
		2	17	705.24	182.75	611.27	799.20
		5	17	744.65	91.93	697.38	791.91
		10	17	619.71	142.12	546.63	692.78
		15	17	596.47	92.69	548.82	644.13

Supplementary Table 12: **Model Summary Beambreaks Habituation OFT**

**Model Terms**	β	$\boldsymbol{SE}$	**Df**	$\mathbf{CI}_{low}$	$\mathbf{CI}_{up}$	$\boldsymbol{t}$	$\boldsymbol{p}$	$\boldsymbol{R}^2_{part}$
Intercept	887.94	39.72	1822	810.04	965.84	22.35	0	0.4008
ELA	46	50.81	119	-54.61	146.61	0.91	0.3671	0.0234
AT	-41.87	65.87	119	-172.29	88.56	-0.64	0.5263	0.021
WT	-5.27	46.15	119	-96.66	86.11	-0.11	0.9093	0.0032
Male	-231.67	56.17	119	-342.9	-120.44	-4.12	0.0001	0.0023
Min	-19.04	2.68	1822	-24.29	-13.78	-7.1	0	0.0011
ELA:AT	-73.48	80.29	119	-232.47	85.52	-0.92	0.362	0.0011
ELA:WT	-31.76	61.51	119	-153.56	90.04	-0.52	0.6066	0.0011
ELA:Male	-2.86	71.86	119	-145.14	139.43	-0.04	0.9684	0.001
AT:Male	117.84	86.57	119	-53.58	289.25	1.36	0.176	0.001
WT:Male	3.67	66.66	119	-128.33	135.67	0.06	0.9562	0.0008
ELA:Min	4.49	3.43	1822	-2.24	11.21	1.31	0.1908	0.0007
AT:Min	4.44	4.44	1822	-4.28	13.15	1	0.3183	0.0005
WT:Min	-2.08	3.11	1822	-8.19	4.03	-0.67	0.5039	0.0005
Male:Min	9.85	3.79	1822	2.41	17.28	2.6	0.0095	0.0003
ELA:AT:Male	-86.08	113.21	119	-310.24	138.08	-0.76	0.4485	0.0002
ELA:WT:Male	77.84	88.04	119	-96.49	252.18	0.88	0.3784	0.0001
ELA:AT:Min	-2.33	5.42	1822	-12.96	8.3	-0.43	0.667	0.0001
ELA:WT:Min	1.11	4.15	1822	-7.03	9.25	0.27	0.7895	0.0001
ELA:Male:Min	-2.55	4.85	1822	-12.06	6.96	-0.53	0.5988	0
AT:Male:Min	-8.99	5.84	1822	-20.45	2.46	-1.54	0.1239	0
WT:Male:Min	-1.52	4.5	1822	-10.34	7.31	-0.34	0.7363	0
ELA:AT:Male:Min	11.74	7.64	1822	-3.25	26.72	1.54	0.1246	0
ELA:WT:Male:Min	-1.11	5.94	1822	-12.76	10.55	-0.19	0.8524	0

Model formula:
lme(~ Group*Strain*Sex*Minute, random = ~ 1|ID/Minute)
$R^2_{marg} = 32.54\%$, $R^2_{cond} = 93.57\%$
Abbreviations: $\beta = \beta$ coefficient estimate, SE = standard error, Df = degrees of freedom, CI = 95% confidence interval, part = partial, group = early life condition group, Min = minute, marg = marginalized, cond = conditioned,

Supplementary Table 13: **ANOVA Beambreaks Habituation OFT**

**Model Terms**	**Df**$_{num}$	$\boldsymbol{F}$	$\boldsymbol{GES}$	$\boldsymbol{p}$	$\epsilon^2_{partial}$ **Estimate**	**CI**$_{low}$	**CI**$_{up}$	**Cohen's F**$_{partial}$ **Estimate**	**CI**$_{low}$	**CI**$_{up}$
**Group**	1	14.25	0.0427	**0.00025**	0.0994	0.0212	0.2104	0.3461	0.1604	0.5303
Strain	2	1.18	0.0074	0.30961	0.003	0	0.0336	0.1411	0	0.3007
**Sex**	1	82.81	0.206	**<.00001**	0.4054	0.2753	0.5159	0.8342	0.6243	1.0414
Group:Strain	2	2.78	0.0171	*0.06599*	0.0286	0	0.1007	0.2162	0	0.3829
Group:Sex	1	0.25	0.0008	0.62031	-0.0063	0	0	0.0455	0	0.2225
Strain:Sex	2	0.66	0.0041	0.51785	-0.0056	0	0	0.1055	0	0.2592
Group:Strain:Sex	2	0.54	0.0034	0.58244	-0.0076	0	0	0.0955	0	0.2468
**Minute**	9	36.25	0.1604	**<.00001**	0.227	0.189	0.2559	0.5519	0.4927	0.5966
**Group:Minute**	9	1.88	0.0098	**0.04818**	0.0072	0	0.0078	0.1255	0.0029	0.1754
Strain:Minute	19	1.16	0.012	0.28781	0.0026	0	0	0.1394	0	0.1272
**Sex:Minute**	9	4.22	0.0218	**0.00001**	0.0261	0.0068	0.0349	0.1883	0.1134	0.2175
Group:Strain:Minute	19	0.59	0.0061	0.91834	-0.0069	0	0	0.0992	0	0.0352
Group:Sex:Minute	9	0.71	0.0037	0.70443	-0.0024	0	0	0.0775	0	0.0743
Strain:Sex:Minute	19	0.87	0.0091	0.62547	-0.0022	0	0	0.1207	0	0.0947
Group:Strain:Sex:Minute	19	0.72	0.0075	0.80498	-0.0047	0	0	0.1097	0	0.0704

Model formula:
lme(~ Group*Strain*Sex*Minute, random = ~ 1|ID/Minute)
$Df_{denBetween} = 119$, $MSE_{between} = 117384$, $Df_{denWithin} = 1130$, $MSE_{within} = 20806$
Abbreviations: group = early life condition group, num = numerator, den = denominator, Df = degrees of freedom, *MSE* = mean standard error, *GES* = generalized η^2, CI = 95% confidence interval

Supplementary Table 14: **Descriptive Nocturnal Distance [m]**

Strain	Sex	Group	N	Mean	SD	95% CI low	95% CI up
CG	Female	Control	7	2586.16	1117.10	1553.01	3619.30
		ELA	11	4218.35	899.67	3613.95	4822.76
	Male	Control	7	2259.04	353.83	1931.80	2586.28
		ELA	11	2700.24	793.25	2167.33	3233.15
AT	Female	Control	4	3264.80	610.13	2293.94	4235.66
		ELA	10	3044.38	864.49	2425.96	3662.80
	Male	Control	7	2626.46	617.70	2055.18	3197.73
		ELA	5	2455.84	376.49	1988.37	2923.31
WT	Female	Control	20	3199.28	948.47	2755.38	3643.17
		ELA	17	3570.82	967.43	3073.41	4068.23
	Male	Control	15	2466.61	662.09	2099.95	2833.26
		ELA	17	2579.88	470.53	2337.96	2821.81

Supplementary Table 15: **Model Summary Nocturnal Distance OFT**

**Model Terms**	β	***SE***	***t***	***p***	**CI**$_{low}$	**CI**$_{up}$
Intercept	2586.16	300.2	8.61	<0.00001	1991.73	3180.59
ELA	1632.2	384.02	4.25	0.00004	871.8	2392.59
AT	678.64	497.83	1.36	0.17539	-307.11	1664.39
WT	613.12	348.8	1.76	0.08136	-77.55	1303.78
Male	-327.11	424.55	-0.77	0.44253	-1167.76	513.54
ELA:AT	-1852.62	606.85	-3.05	0.0028	-3054.24	-650.99
ELA:WT	-1260.65	464.89	-2.71	0.00768	-2181.18	-340.13
ELA:Male	-1191	543.09	-2.19	0.03025	-2266.37	-115.64
AT:Male	-311.23	654.27	-0.48	0.63517	-1606.76	984.3
WT:Male	-405.55	503.83	-0.8	0.42246	-1403.18	592.07
ELA:AT:Male	1240.81	855.59	1.45	0.14962	-453.34	2934.95
ELA:WT:Male	932.74	665.4	1.4	0.16359	-384.82	2250.3

Model formula:
lm(~ Group*Strain*Sex)
$R^2 = 34\%$, $R^2_{adj} = 28\%$, $RMSE = 794$, $\text{Df}_{num} = 12$, $\text{Df}_{den} = 119$, $\text{F}_{mod} = 5.6$
Abbreviations: β = β coefficient estimate, *SE* = standard error, CI = 95% confidence interval, group = early life condition group, adj = adjusted, *RMSE* = root mean square error (sigma), Df = degrees of freedom, num = numerator, den = denominator, mod = model

Supplementary Table 16: **ANOVA Nocturnal Distance OFT**

**Model Terms**	**Sum of Squares**	**Df**	$\boldsymbol{F}$	$\boldsymbol{p}$	$\eta^2_{partial}$ **Estimate**	$\eta^2_{partial}$ $\mathbf{CI}_{low}$	$\eta^2_{partial}$ $\mathbf{CI}_{up}$	$\epsilon^2_{partial}$ **Estimate**	$\epsilon^2_{partial}$ $\mathbf{CI}_{low}$	$\epsilon^2_{partial}$ $\mathbf{CI}_{up}$
**Group**	4618308.48	1	7.32	**0.00782**	0.06	0	0.15	0.05	0	0.14
Strain	1068955.1	2	0.85	0.43117	0.01	0	0.05	-0.01	0	0
**Sex**	24161371.46	1	38.3	<**0.00001**	0.23	0.11	0.36	0.23	0.11	0.35
**Group:Strain**	5942021.42	2	4.71	**0.01075**	0.08	0.01	0.18	0.07	0	0.16
Group:Sex	1630636.87	1	2.58	0.11054	0.02	0	0.1	0.01	0	0.08
Strain:Sex	646520.39	2	0.51	0.60036	0.01	0	0.06	-0.01	0	0
Group:Strain:Sex	1691604.95	2	1.34	0.26558	0.02	0	0.09	0.01	0	0.05

Model formula: lm(~ Group*Strain*Sex)
Sum of Squares$_{residual}$ = 8^7, Df$_{residual}$ = 119
Abbreviations: group = early life condition group, Df = degrees of freedom, CI = 95% confidence interval

Supplementary Table 17: **Descriptive Average Time [s/min] in the Dark Compartment**

Strain	Sex	Group	N	Mean	SD	95% CI low	95% CI up
CG	Female	Control	7	40.29	5.75	34.97	45.60
		ELA	11	31.15	10.12	24.36	37.95
	Male	Control	7	41.73	4.80	37.29	46.17
		ELA	11	32.68	9.66	26.19	39.17
AT	Female	Control	5	40.70	4.85	34.68	46.72
		ELA	10	38.93	9.71	31.98	45.88
	Male	Control	7	42.24	6.66	36.09	48.40
		ELA	5	41.42	2.75	38.00	44.84
WT	Female	Control	20	41.29	6.42	38.29	44.29
		ELA	17	34.96	11.24	29.19	40.74
	Male	Control	15	42.99	6.50	39.38	46.59
		ELA	17	33.16	7.73	29.19	37.14

Supplementary Table 18: **Model Summary Dark-Light-Test**

Model Terms	β	SE	t	p	CI_{low}	CI_{up}
Intercept	40.29	3.06	13.15	¡0.00001	34.22	46.35
ELA	-9.13	3.92	-2.33	0.02148	-16.89	-1.37
AT	0.41	4.75	0.09	0.93059	-8.98	9.81
WT	1	3.56	0.28	0.77834	-6.04	8.05
Male	1.44	4.33	0.33	0.73971	-7.14	10.02
ELA:AT	7.36	5.92	1.24	0.2163	-4.36	19.09
ELA:WT	2.81	4.74	0.59	0.55537	-6.59	12.2
ELA:Male	0.08	5.54	0.02	0.98787	-10.89	11.06
AT:Male	0.1	6.43	0.02	0.98761	-12.62	12.82
WT:Male	0.25	5.14	0.05	0.96071	-9.93	10.43
ELA:AT:Male	0.86	8.54	0.1	0.91972	-16.05	17.77
ELA:WT:Male	-3.58	6.79	-0.53	0.59893	-17.03	9.86

Model formula:
lm(~ Group*Strain*Sex)
R^2 = 23%, R^2_{adj} = 16%, $RMSE$ = 8.1, Df_{num} = 12, Df_{den} = 120, F_{mod} = 3.2
Abbreviations: β = β coefficient estimate, SE = standard error, CI = 95% confidence interval, group = early life condition group, adj = adjusted, $RMSE$ = root mean square error (sigma), Df = degrees of freedom, num = numerator, den = denominator, mod = model

Supplementary Table 19: **ANOVA Time in the Dark Compartment during Dark-Light Test**

**Model Terms**	**Sum of Squares**	**Df**	***F***	***p***	$\eta^2_{partial}$			$\epsilon^2_{partial}$		
					**Estimate**	$\mathbf{CI}_{low}$	$\mathbf{CI}_{up}$	**Estimate**	$\mathbf{CI}_{low}$	$\mathbf{CI}_{up}$
**Group**	1548.2	1	23.56	**<0.00001**	0.17	0.07	0.29	0.17	0.06	0.29
Strain	332.12	2	2.53	*0.08412*	0.04	0	0.12	0.03	0	0.1
Sex	20.11	1	0.31	0.58117	0	0	0.04	-0.01	0	0
Group:Strain	251.9	2	1.92	0.15154	0.03	0	0.1	0.01	0	0.07
Group:Sex	22.11	1	0.34	0.56292	0	0	0.05	-0.01	0	0
Strain:Sex	29.62	2	0.23	0.79854	0	0	0.04	-0.01	0	0
Group:Strain:Sex	31.48	2	0.24	0.78737	0	0	0.04	-0.01	0	0

Model formula: lm(~ Group*Strain*Sex)
Sum of Squares$_{residual}$ = 7885, Df$_{residual}$ = 120
Abbreviations: group = early life condition group, Df = degrees of freedom, CI = 95% confidence interval

Supplementary Table 20: **Descriptive Alternations [%] T-Maze**

Read Out	Strain	Sex	Group	N	Mean	SD	95% CI low	95% CI up
Alternations	CG	Female	Control	7	57.14	19.34	39.25	75.03
			ELA	11	61.04	18.45	48.64	73.43
		Male	Control	7	55.10	27.27	29.89	80.32
			ELA	11	56.49	11.27	48.92	64.07
	AT	Female	Control	5	61.43	12.98	45.32	77.54
			ELA	10	46.43	10.24	39.10	53.75
		Male	Control	7	58.16	8.68	50.14	66.19
			ELA	5	48.57	7.82	38.86	58.29
	WT	Female	Control	20	51.07	21.40	41.06	61.09
			ELA	17	55.04	15.92	46.85	63.23
		Male	Control	15	55.71	18.35	45.55	65.88
			ELA	17	60.50	17.33	51.59	69.42

Supplementary Table 21: **Model Summary Alternations T-Maze**

Model Terms	β	SE	t	p	CI_{low}	CI_{up}
Intercept	57.14	6.54	8.73	<0.00001	44.19	70.1
ELA	3.9	8.37	0.47	0.64249	-12.68	20.47
AT	4.29	10.14	0.42	0.67326	-15.79	24.36
WT	-6.07	7.6	-0.8	0.42618	-21.13	8.98
Male	-2.04	9.26	-0.22	0.82585	-20.37	16.28
ELA:AT	-18.9	12.65	-1.49	0.13787	-43.94	6.15
ELA:WT	0.07	10.13	0.01	0.99415	-19.99	20.14
ELA:Male	-2.5	11.84	-0.21	0.83281	-25.95	20.94
AT:Male	-1.22	13.73	-0.09	0.92907	-28.4	25.96
WT:Male	6.68	10.98	0.61	0.54399	-15.06	28.43
ELA:AT:Male	7.91	18.25	0.43	0.6653	-28.21	44.04
ELA:WT:Male	3.32	14.51	0.23	0.81914	-25.4	32.04

Model formula:
lm(~ Group*Strain*Sex)
R^2 = 6%, R^2_{adj} = -2%, $RMSE$ = 17.3, Df_{num} = 12, Df_{den} = 120, F_{mod} = 0.75
Abbreviations: β = β coefficient estimate, SE = standard error, CI = 95% confidence interval, group = early life condition group, adj = adjusted, $RMSE$ = root mean square error (sigma), Df = degrees of freedom, num = numerator, den = denominator, mod = model

Supplementary Table 22: **ANOVA T-Maze Alternations**

**Model Terms**	**Sum of Squares**	**Df**	$\boldsymbol{F}$	$\boldsymbol{p}$	$\eta^2_{partial}$			$\epsilon^2_{partial}$		
					**Estimate**	**CI**$_{low}$	**CI**$_{up}$	**Estimate**	**CI**$_{low}$	**CI**$_{up}$
Group	11.65	1	0.04	0.84405	0	0	0.03	-0.01	0	0
Strain	368.76	2	0.62	0.54233	0.01	0	0.06	-0.01	0	0
Sex	82.7	1	0.28	0.60041	0.01	0	0.06	0	0	0
Group:Strain	1345.09	2	2.24	0.11054	0.04	0	0.11	0.02	0	0.08
Group:Sex	5.39	1	0.02	0.89354	0	0	0	-0.01	0	0
Strain:Sex	473.81	2	0.79	0.4561	0.01	0	0.07	0	0	0
Group:Strain:Sex	56.39	2	0.09	0.91031	0	0	0.02	-0.02	0	0

Model formula: lm(~ Group*Strain*Sex)

Sum of Squares$_{residual}$ = 35976, Df$_{residual}$ = 120

Abbreviations: group = early life condition group, Df = degrees of freedom, CI = 95% confidence interval

Supplementary Table 23: **Descriptive Latency to Completion of the T-Maze**

Read Out	Strain	Sex	Group	N	Mean	SD	95% CI low	95% CI up
Latency	CG	Female	Control	7	8.71	3.50	5.48	11.95
			ELA	11	5.55	1.81	4.33	6.76
		Male	Control	6	10.83	3.13	7.55	14.11
			ELA	11	7.82	1.25	6.98	8.66
	AT	Female	Control	5	7.60	1.67	5.52	9.68
			ELA	10	6	2.21	4.42	7.58
		Male	Control	7	9.57	2.30	7.45	11.70
			ELA	5	8.20	3.77	3.52	12.88
	WT	Female	Control	18	8.72	3.18	7.14	10.30
			ELA	17	6.24	1.44	5.50	6.97
		Male	Control	14	9.50	2.24	8.20	10.80
			ELA	17	8.94	2.22	7.80	10.08

Supplementary Table 24: **Model Summary Latency Completion T-maze**

Model Terms	β	*SE*	*t*	*p*	CI_{low}	CI_{up}
Intercept	8.71	0.9	9.64	<0.00001	6.92	10.5
ELA	-3.17	1.16	-2.74	0.00711	-5.46	-0.88
AT	-1.11	1.4	-0.8	0.42782	-3.89	1.66
WT	0.01	1.07	0.01	0.99407	-2.1	2.12
Male	2.12	1.33	1.59	0.11396	-0.52	4.75
ELA:AT	1.57	1.75	0.9	0.3711	-1.89	5.03
ELA:WT	0.68	1.41	0.48	0.62983	-2.11	3.48
ELA:Male	0.15	1.68	0.09	0.92711	-3.17	3.47
AT:Male	-0.15	1.93	-0.08	0.93922	-3.97	3.68
WT:Male	-1.34	1.58	-0.85	0.3977	-4.47	1.79
ELA:AT:Male	0.07	2.55	0.03	0.97659	-4.97	5.12
ELA:WT:Male	1.77	2.05	0.86	0.38889	-2.29	5.84

Model formula:
lm(~ Group*Strain*Sex)
R^2 = 30%, R^2_{adj} = 23%, *RMSE* = 2.4, Df_{num} = 12, Df_{den} = 116, F_{mod} = 4.5
Abbreviations: β = β coefficient estimate, *SE* = standard error, CI = 95% confidence interval, group = early life condition group, adj = adjusted, *RMSE* = root mean square error (sigma), Df = degrees of freedom, num = numerator, den = denominator, mod = model

Supplementary Table 25: **ANOVA T-Maze Latency Completion**

**Model Terms**	**Sum of Squares**	**Df**	***F***	***p***	$\eta^2_{partial}$ **Estimate**	$\eta^2_{partial}$ **CI**$_{low}$	$\eta^2_{partial}$ **CI**$_{up}$	$\epsilon^2_{partial}$ **Estimate**	$\epsilon^2_{partial}$ **CI**$_{low}$	$\epsilon^2_{partial}$ **CI**$_{up}$
**Group**	119.22	1	20.85	**0.00001**	0.16	0.06	0.28	0.15	0.05	0.28
Strain	4.7	2	0.41	0.66414	0.01	0	0.06	-0.01	0	0
**Sex**	120.84	1	21.13	**0.00001**	0.15	0.05	0.27	0.15	0.05	0.27
Group:Strain	14.36	2	1.26	0.28873	0.02	0	0.09	0	0	0.04
Group:Sex	9.52	1	1.66	0.19955	0.01	0	0.09	0.01	0	0.06
Strain:Sex	0.74	2	0.06	0.93765	0	0	0.02	-0.02	0	0
Group:Strain:Sex	5.81	2	0.51	0.6033	0.01	0	0.06	-0.01	0	0

Model formula: lm(~ Group*Strain*Sex)

Sum of Squares$_{residual}$ = 663, Df$_{residual}$ = 116

Abbreviations: group = early life condition group, Df = degrees of freedom, CI = 95% confidence interval

Supplementary Table 26: **Descriptive Social Interaction** [% time]

**Strain**	**Sex**	**Group**	**Compartment**	**N**	**Mean**	**SD**	**95% CI low**	**95% CI up**
CG	Female	Control	Reference	7	3.343	1.767	1.709	4.977
			Social	7	11.071	1.442	9.738	12.405
		ELA	Reference	11	3.936	6.307	-0.301	8.173
			Social	11	5.864	4.883	2.583	9.144
	Male	Control	Reference	7	1.957	1.811	0.282	3.632
			Social	7	10.986	6.998	4.513	17.458
		ELA	Reference	11	3.000	1.872	1.742	4.258
			Social	11	10.100	6.727	5.581	14.619
AT	Female	Control	Reference	5	1.960	1.260	0.395	3.525
			Social	5	6.120	1.840	3.835	8.405
		ELA	Reference	10	5.070	7.379	-0.209	10.349
			Social	10	7.680	4.878	4.190	11.170
	Male	Control	Reference	7	2.643	1.512	1.244	4.041
			Social	7	12.771	3.582	9.459	16.084
		ELA	Reference	5	1.380	1.341	-0.284	3.044
			Social	5	6.000	3.694	1.413	10.587
WT	Female	Control	Reference	16	4.088	3.509	2.218	5.957
			Social	16	8.144	5.467	5.231	11.057
		ELA	Reference	16	3.269	2.256	2.066	4.471
			Social	16	8.781	5.098	6.065	11.498
	Male	Control	Reference	11	3.782	2.020	2.425	5.139
			Social	11	10.927	4.814	7.693	14.161
		ELA	Reference	14	3.129	1.715	2.138	4.119
			Social	14	9.871	4.787	7.107	12.635

Supplementary Table 27: **Model Summary Interaction Time SCT**

**Model Terms**	β	$\boldsymbol{SE}$	**Df**	**CI**$_{low}$	**CI**$_{up}$	$\boldsymbol{t}$	$\boldsymbol{p}$	$\boldsymbol{R^2_{part}}$
Intercept	3.34	1.64	108	0.1	6.59	2.04	0.0436	0.3595
ELA	0.59	2.09	108	-3.56	4.74	0.28	0.7774	0.0451
AT	-1.38	2.54	108	-6.41	3.64	-0.55	0.5867	0.0163
WT	0.74	1.96	108	-3.15	4.64	0.38	0.7052	0.016
Male	-1.39	2.32	108	-5.98	3.2	-0.6	0.5508	0.0074
Social	7.73	2.32	108	3.14	12.32	3.34	0.0012	0.0062
ELA:AT	2.52	3.16	108	-3.76	8.79	0.8	0.4283	0.0049
ELA:WT	-1.41	2.59	108	-6.56	3.73	-0.54	0.5873	0.0047
ELA:Male	0.45	2.96	108	-5.42	6.32	0.15	0.8797	0.0042
AT:Male	2.07	3.43	108	-4.74	8.88	0.6	0.5482	0.0039
WT:Male	1.08	2.87	108	-4.61	6.77	0.38	0.7075	0.0038
ELA:Social	-5.8	2.96	108	-11.67	0.07	-1.96	0.0527	0.0036
AT:Social	-3.57	3.59	108	-10.68	3.54	-0.99	0.322	0.0027
WT:Social	-3.67	2.78	108	-9.17	1.83	-1.32	0.1887	0.0015
Male:Social	1.3	3.27	108	-5.19	7.79	0.4	0.6921	0.0015
ELA:AT:Male	-4.82	4.56	108	-13.87	4.23	-1.06	0.2931	0.0013
ELA:WT:Male	-0.28	3.76	108	-7.74	7.18	-0.08	0.94	0.0013
ELA:AT:Social	4.25	4.48	108	-4.62	13.12	0.95	0.3443	0.0008
ELA:WT:Social	7.26	3.67	108	-0.02	14.53	1.98	0.0505	0.0007
ELA:Male:Social	3.87	4.19	108	-4.43	12.18	0.92	0.3573	0.0006
AT:Male:Social	4.67	4.86	108	-4.96	14.3	0.96	0.3386	0.0006
WT:Male:Social	1.79	4.06	108	-6.26	9.84	0.44	0.6603	0.0003
ELA:AT:Male:Social	-7.83	6.46	108	-20.63	4.96	-1.21	0.2277	0.0001
ELA:WT:Male:Social	-5.73	5.32	108	-16.28	4.82	-1.08	0.2839	0

Model formula:
lme(~ Group*Strain*Sex*Compartment, random = ~ 1|ID/Compartment)
$R^2_{marg} = 35.66\%$, $R^2_{cond} > 99.99\%$
Abbreviations: β = β coefficient estimate, SE = standard error, Df = degrees of freedom, CI = 95% confidence interval, part = partial, group = early life condition group, marg = marginalized, cond = conditioned,

Supplementary Table 28: **ANOVA Percent Interaction Time SCT**

**Model Terms**	**Df**$_{num}$	$\boldsymbol{F}$	$\boldsymbol{GES}$	$\boldsymbol{p}$	$\epsilon^2_{partial}$			**Cohen's F**$_{partial}$		
					**Estimate**	**CI**$_{low}$	**CI**$_{up}$	**Estimate**	**CI**$_{low}$	**CI**$_{up}$
Group	1	1.45	0.0064	0.23122	0.0041	0	0.0597	0.1159	0	0.3048
Strain	2	0.49	0.0044	0.61265	-0.0093	0	0	0.0955	0	0.253
Sex	1	1.5	0.0066	0.22367	0.0045	0	0.0613	0.1178	0	0.3067
Group:Strain	2	0.12	0.001	0.89072	-0.0163	0	0	0.0463	0	0.1702
Group:Sex	1	0.86	0.0038	0.35717	-0.0013	0	0	0.089	0	0.2776
Strain:Sex	2	0.08	0.0007	0.92318	-0.017	0	0	0.0385	0	0.1485
**Group:Strain:Sex**	2	3.84	0.033	**0.02445**	0.0491	0	0.1394	0.2667	0.0107	0.4447
**Compartment**	1	99.86	0.3245	**<.00001**	0.4756	0.3436	0.5812	0.9616	0.7321	1.188
Group:Compartment	1	1.8	0.0086	0.18215	0.0073	0	0.07	0.1292	0	0.3183
Strain:Compartment	2	0.12	0.0011	0.88897	-0.0163	0	0	0.0467	0	0.1712
**Sex:Compartment**	1	6.55	0.0306	**0.01185**	0.0485	0.0006	0.1476	0.2463	0.0524	0.4372
Group:Strain:Compartment	2	1.71	0.0162	0.18609	0.0127	0	0.0705	0.1778	0	0.3488
Group:Sex:Compartment	1	0.07	0.0003	0.7956	-0.0086	0	0	0.025	0	0.1995
Strain:Sex:Compartment	2	0.26	0.0025	0.76916	-0.0136	0	0	0.0698	0	0.2165
Group:Strain:Sex:Compartment	2	0.84	0.0081	0.43266	-0.0028	0	0	0.125	0	0.2892

Model formula:
lme(~ Group*Strain*Sex*Compartment, random = ~ 1|ID/Compartment)
$Df_{denBetween} = 108$, $MSE_{between} = 18$, $Df_{denWithin} = 108$, $MSE_{within} = 19.5$
Abbreviations: group = early life condition group, num = numerator, den = denominator, Df = degrees of freedom, *MSE* = mean standard error, *GES* = generalized η^2, CI = 95% confidence interval

Supplementary Table 29: **Descriptive Social Distance [% time]**

**Strain**	**Sex**	**Group**	**Compartment**	**N**	**Mean**	**SD**	**95% CI low**	**95% CI up**
CG	Female	Control	Reference	7	7.51	2.50	5.20	9.83
			Social	7	10.93	2.26	8.84	13.02
		ELA	Reference	11	8.56	4.22	5.73	11.40
			Social	11	21.39	7.70	16.22	26.57
	Male	Control	Reference	7	8.33	3.92	4.71	11.95
			Social	7	15.87	7.39	9.03	22.71
		ELA	Reference	11	7.70	3.00	5.69	9.71
			Social	11	15.73	2.81	13.84	17.62
AT	Female	Control	Reference	5	7.60	1.75	5.42	9.78
			Social	5	15.36	10.48	2.34	28.38
		ELA	Reference	10	7.59	4.52	4.36	10.82
			Social	10	13.07	6.70	8.28	17.86
	Male	Control	Reference	7	7.20	2.49	4.89	9.51
			Social	7	18.43	4.53	14.24	22.62
		ELA	Reference	5	3.90	2.74	0.50	7.30
			Social	5	11.72	9.51	-0.08	23.52
WT	Female	Control	Reference	16	8.77	3.78	6.76	10.78
			Social	16	11.49	4.82	8.93	14.06
		ELA	Reference	16	7.40	3.59	5.49	9.31
			Social	16	13.06	5.91	9.91	16.21
	Male	Control	Reference	11	7.04	4.33	4.13	9.95
			Social	11	13.89	5.47	10.21	17.57
		ELA	Reference	14	6.82	3.67	4.70	8.94
			Social	14	13.89	7.01	9.84	17.93

Supplementary Table 30: **Model Summary Distant Observation Time SCT**

**Model Terms**	β	$\boldsymbol{SE}$	**Df**	$\mathbf{CI}_{low}$	$\mathbf{CI}_{up}$	$\boldsymbol{t}$	$\boldsymbol{p}$	$\boldsymbol{R}^2_{part}$
Intercept	7.51	1.92	108	3.7	11.33	3.9	0.0002	0.3928
ELA	1.05	2.46	108	-3.83	5.93	0.43	0.6708	0.03
AT	0.09	2.98	108	-5.82	6	0.03	0.9771	0.0205
WT	1.25	2.31	108	-3.32	5.83	0.54	0.5878	0.0137
Male	0.81	2.72	108	-4.58	6.21	0.3	0.7654	0.0095
Social	3.41	2.72	108	-1.97	8.8	1.26	0.2115	0.0066
ELA:AT	-1.06	3.72	108	-8.43	6.31	-0.28	0.7764	0.0049
ELA:WT	-2.42	3.05	108	-8.46	3.63	-0.79	0.4296	0.0045
ELA:Male	-1.68	3.48	108	-8.58	5.22	-0.48	0.6308	0.0045
AT:Male	-1.21	4.04	108	-9.22	6.79	-0.3	0.7641	0.0042
WT:Male	-2.55	3.37	108	-9.23	4.14	-0.75	0.452	0.0027
ELA:Social	9.41	3.47	108	2.53	16.3	2.71	0.0078	0.0024
AT:Social	4.35	4.21	108	-4	12.69	1.03	0.304	0.0017
WT:Social	-0.69	3.26	108	-7.14	5.77	-0.21	0.8328	0.0013
Male:Social	4.13	3.84	108	-3.49	11.74	1.07	0.2849	0.001
ELA:AT:Male	-1.61	5.37	108	-12.25	9.02	-0.3	0.7644	0.0008
ELA:WT:Male	2.83	4.42	108	-5.94	11.6	0.64	0.5235	0.0004
ELA:AT:Social	-11.69	5.25	108	-22.1	-1.29	-2.23	0.028	0.0004
ELA:WT:Social	-6.48	4.3	108	-15.01	2.05	-1.51	0.135	0.0004
ELA:Male:Social	-8.93	4.91	108	-18.67	0.81	-1.82	0.072	0.0003
AT:Male:Social	-0.66	5.7	108	-11.95	10.63	-0.12	0.908	0.0002
WT:Male:Social	0	4.76	108	-9.44	9.44	0	0.9998	0.0001
ELA:AT:Male:Social	7.8	7.57	108	-7.21	22.81	1.03	0.3053	0
ELA:WT:Male:Social	6.21	6.24	108	-6.17	18.58	0.99	0.3224	0

Model formula:
lme(~ Group*Strain*Sex*Compartment, random = ~ 1|ID/Compartment)
R^2_{marg} = 38.98%, R^2_{cond} = 98.93%
Abbreviations: β = β coefficient estimate, SE = standard error, Df = degrees of freedom, CI = 95% confidence interval, part = partial, group = early life condition group, marg = marginalized, cond = conditioned

Supplementary Table 31: **ANOVA Percent Distant Observation Time SCT**

**Model Terms**	**Df**$_{num}$	***F***	***GES***	***p***	$\epsilon^2_{partial}$			**Cohen's F**$_{partial}$		
					**Estimate**	**CI**$_{low}$	**CI**$_{up}$	**Estimate**	**CI**$_{low}$	**CI**$_{up}$
Group	1	0.06	0.0003	0.81169	-0.0087	0	0	0.023	0	0.1952
**Strain**	2	3.54	0.0318	**0.03248**	0.0441	0	0.1315	0.256	0	0.4332
Sex	1	0.23	0.0011	0.63522	-0.0071	0	0	0.0458	0	0.2312
**Group:Strain**	2	4.6	0.041	**0.01212**	0.0614	0	0.1579	0.2918	0.0642	0.4713
**Group:Sex**	1	4.22	0.0192	**0.04234**	0.0287	0	0.1156	0.1977	0	0.3877
Strain:Sex	2	0.23	0.0021	0.79381	-0.0142	0	0	0.0655	0	0.2094
Group:Strain:Sex	2	1.88	0.0172	0.1571	0.0158	0	0.0784	0.1867	0	0.3586
**Compartment**	1	110.04	0.3366	**<.00001**	0.5001	0.3708	0.6019	1.0094	0.7762	1.2394
Group:Compartment	1	1.64	0.0075	0.20299	0.0058	0	0.0656	0.1233	0	0.3123
Strain:Compartment	2	1.82	0.0165	0.16659	0.0147	0	0.0756	0.1837	0	0.3553
Sex:Compartment	1	1.25	0.0057	0.26652	0.0023	0	0.0517	0.1075	0	0.2963
Group:Strain:Compartment	2	2.18	0.0197	0.11796	0.021	0	0.0897	0.2009	0	0.3741
Group:Sex:Compartment	1	2.48	0.0113	0.11810	0.0134	0	0.0854	0.1516	0	0.3409
Strain:Sex:Compartment	2	0.84	0.0077	0.43498	-0.0029	0	0	0.1246	0	0.2887
Group:Strain:Sex:Compartment	2	0.68	0.0062	0.50942	-0.0059	0	0	0.1121	0	0.2738

Model formula:
lme(~ Group*Strain*Sex*Compartment, random = ~ 1|ID/Compartment)
$\text{Df}_{denBetween} = 108$, $MSE_{between} = 26$, $\text{Df}_{denWithin} = 108$, $MSE_{within} = 26$
Abbreviations: group = early life condition group, num = numerator, den = denominator, Df = degrees of freedom, *MSE* = mean standard error, *GES* = generalized η^2, CI = 95% confidence interval

Supplementary Table 32: **Descriptive Distance [m] Social Zone**

Strain	Sex	Group	Compartment	N	Mean	SD	95% CI low	95% CI up
CG	Female	Control	Reference	7	0.70	0.24	0.47	0.92
			Social	7	0.77	0.25	0.54	1.00
		ELA	Reference	11	0.82	0.27	0.63	1.00
			Social	11	1.92	0.75	1.42	2.43
	Male	Control	Reference	7	0.61	0.23	0.40	0.81
			Social	7	1.12	0.55	0.62	1.63
		ELA	Reference	11	0.48	0.16	0.38	0.59
			Social	11	1.01	0.23	0.85	1.16
AT	Female	Control	Reference	5	0.73	0.20	0.49	0.98
			Social	5	0.89	0.42	0.37	1.41
		ELA	Reference	10	0.76	0.48	0.41	1.10
			Social	10	1.19	0.64	0.73	1.65
	Male	Control	Reference	7	0.69	0.27	0.44	0.94
			Social	7	1.36	0.41	0.97	1.74
		ELA	Reference	5	0.39	0.33	-0.02	0.81
			Social	5	0.68	0.50	0.05	1.30
WT	Female	Control	Reference	16	0.68	0.28	0.53	0.83
			Social	16	0.73	0.32	0.56	0.90
		ELA	Reference	16	0.67	0.31	0.50	0.84
			Social	16	1.10	0.59	0.78	1.41
	Male	Control	Reference	11	0.52	0.30	0.32	0.72
			Social	11	0.87	0.39	0.60	1.13
		ELA	Reference	14	0.54	0.23	0.40	0.67
			Social	14	0.97	0.67	0.58	1.36

Supplementary Table 33: **Model Summary Distance Social Zone**

**Model Terms**	β	$\boldsymbol{SE}$	**Df**	**CI**$_{low}$	**CI**$_{up}$	$\boldsymbol{t}$	$\boldsymbol{p}$	$\boldsymbol{R}^2_{part}$
(Intercept)	0.7	0.16	108	0.38	1.01	4.4	0	0.3772
ELA	0.12	0.2	108	-0.28	0.52	0.58	0.5599	0.0523
AT	0.04	0.25	108	-0.45	0.52	0.15	0.8842	0.0265
WT	-0.02	0.19	108	-0.4	0.36	-0.1	0.917	0.0143
Male	-0.09	0.22	108	-0.54	0.35	-0.41	0.6831	0.0129
Social	0.08	0.22	108	-0.35	0.51	0.35	0.7276	0.0087
ELA:AT	-0.1	0.31	108	-0.7	0.51	-0.31	0.7549	0.0082
ELA:WT	-0.13	0.25	108	-0.62	0.37	-0.5	0.6162	0.003
ELA:Male	-0.24	0.29	108	-0.81	0.33	-0.84	0.4038	0.0022
AT:Male	0.05	0.33	108	-0.61	0.71	0.16	0.8769	0.0015
WT:Male	-0.06	0.28	108	-0.61	0.49	-0.22	0.8247	0.0015
ELA:Social	1.03	0.28	108	0.48	1.58	3.72	0.0003	0.0011
AT:Social	0.09	0.34	108	-0.58	0.75	0.25	0.7996	0.0007
WT:Social	-0.03	0.26	108	-0.54	0.49	-0.1	0.9212	0.0006
Male:Social	0.44	0.31	108	-0.17	1.05	1.44	0.1522	0.0005
ELA:AT:Male	-0.08	0.44	108	-0.96	0.79	-0.19	0.8532	0.0004
ELA:WT:Male	0.26	0.36	108	-0.46	0.98	0.72	0.4758	0.0003
ELA:AT:Social	-0.76	0.42	108	-1.59	0.07	-1.82	0.0722	0.0002
ELA:WT:Social	-0.66	0.34	108	-1.34	0.03	-1.91	0.0589	0.0001
ELA:Male:Social	-1.03	0.39	108	-1.8	-0.25	-2.62	0.0102	0.0001
AT:Male:Social	0.06	0.45	108	-0.84	0.96	0.13	0.8963	0.0001
WT:Male:Social	-0.15	0.38	108	-0.9	0.61	-0.39	0.7009	0.0001
ELA:AT:Male:Social	0.38	0.6	108	-0.82	1.58	0.62	0.534	0
ELA:WT:Male:Social	0.74	0.5	108	-0.25	1.73	1.48	0.1406	0

Model formula:
lme(~ Group*Strain*Sex*Compartment, random = ~ 1|ID/Compartment)
$R^2_{marg} = 37.4\%$, $R^2_{cond} > 96.1\%$
Abbreviations: $\beta = \beta$ coefficient estimate, SE = standard error, Df = degrees of freedom, CI = 95% confidence interval, part = partial, group = early life condition group, marg = marginalized, cond = conditioned

Supplementary Table 34: **ANOVA Distance [m] Social Zone**

**Model Terms**	**Df**$_{num}$	$\boldsymbol{F}$	***GES***	$\boldsymbol{p}$	$\epsilon^2_{partial}$ **Estimate**	$\epsilon^2_{partial}$ **CI**$_{low}$	$\epsilon^2_{partial}$ **CI**$_{up}$	**Cohen's F**$_{partial}$ **Estimate**	**Cohen's F**$_{partial}$ **CI**$_{low}$	**Cohen's F**$_{partial}$ **CI**$_{up}$
Group	1	3.6	0.0174	*0.06037*	0.0233	0	0.1057	0.1826	0	0.3724
Strain	2	4.1	0.0388	**0.01916**	0.0534	0	0.146	0.2757	0.0378	0.4542
Sex	1	8.48	0.0401	**0.00436**	0.0642	0.0047	0.1704	0.2802	0.0872	0.4718
Group:Strain	2	3.35	0.0319	**0.03872**	0.041	0	0.1264	0.2491	0	0.4259
Group:Sex	1	13.84	0.0638	**0.00032**	0.1054	0.0215	0.2235	0.358	0.1627	0.5517
Strain:Sex	2	1.52	0.0148	0.22259	0.0094	0	0.0614	0.168	0	0.3379
Group:Strain:Sex	2	3.35	0.0319	**0.03889**	0.0409	0	0.1263	0.2489	0	0.4257
Compartment	1	65.53	0.2214	$< 1^{-5}$	0.3719	0.2344	0.4913	0.779	0.5623	0.9928
Group:Compartment	1	6.43	0.0271	**0.01264**	0.0475	0.0004	0.1461	0.244	0.0498	0.4349
Strain:Compartment	2	2.56	0.0217	*0.08217*	0.0275	0	0.1027	0.2176	0	0.3921
Sex:Compartment	1	0.18	0.0008	0.67484	-0.0076	0	0	0.0405	0	0.2244
Group:Strain:Compartment	2	1.83	0.0156	0.16553	0.0148	0	0.0759	0.184	0	0.3556
Group:Sex:Compartment	1	7.47	0.0314	**0.00732**	0.0561	0.0024	0.1588	0.2631	0.0701	0.4543
Strain:Sex:Compartment	2	0.77	0.0067	0.46398	-0.0041	0	0	0.1197	0	0.2829
Group:Strain:Sex:Compartment	2	1.11	0.0096	0.33197	0.0021	0	0.0275	0.1436	0	0.3107

Model formula:
lme(~ Group*Strain*Sex*Compartment, random = ~ 1|ID/Compartment)
Df$_{denBetween}$ = 108, $MSE_{between}$ = 0.19, Df$_{denWithin}$ = 108, MSE_{within} = 0.16
Abbreviations: group = early life condition group, num = numerator, den = denominator, Df = degrees of freedom, *MSE* = mean standard error, *GES* = generalized η^2, CI = 95% confidence interval

Supplementary Table 35: **Descriptive Speed [mm/sec] Social Zone**

Strain	Sex	Group	Compartment	N	Mean	SD	95% CI low	95% CI up
CG	Female	Control	Reference	7	31.18	4.59	26.93	35.43
			Social	7	23.15	4.28	19.19	27.11
		ELA	Reference	11	34.27	8.82	28.34	40.20
			Social	11	29.69	4.11	26.93	32.45
	Male	Control	Reference	7	25.55	4.89	21.02	30.07
			Social	7	24.23	3.81	20.71	27.76
		ELA	Reference	11	22.20	6.71	17.69	26.71
			Social	11	21.47	3.97	18.80	24.14
AT	Female	Control	Reference	5	32.61	5.43	25.87	39.35
			Social	5	21.06	4.94	14.93	27.19
		ELA	Reference	10	34.20	6.65	29.44	38.96
			Social	10	29.16	6.88	24.24	34.08
	Male	Control	Reference	7	31.68	4.35	27.65	35.70
			Social	7	24.57	4.26	20.63	28.51
		ELA	Reference	5	34.65	8.77	23.75	45.55
			Social	5	21.57	3.98	16.63	26.51
WT	Female	Control	Reference	16	27.58	9.46	22.54	32.62
			Social	16	21.64	6.28	18.29	24.98
		ELA	Reference	16	30.79	7.09	27.01	34.57
			Social	16	27.61	5.74	24.55	30.67
	Male	Control	Reference	11	26.14	5.56	22.41	29.88
			Social	11	21.00	5.07	17.59	24.41
		ELA	Reference	14	28.00	7.02	23.95	32.05
			Social	14	22.01	6.68	18.15	25.86

Supplementary Table 36: **Model Summary Speed Social Zone**

**Model Terms**	β	$\boldsymbol{SE}$	**Df**	$\mathbf{CI}_{low}$	$\mathbf{CI}_{up}$	$\boldsymbol{t}$	$\boldsymbol{p}$	$\boldsymbol{R}^2_{part}$
(Intercept)	31.18	2.39	108	26.44	35.91	13.05	0	0.3302
ELA	3.09	3.06	108	-2.96	9.15	1.01	0.3135	0.0239
AT	1.43	3.7	108	-5.91	8.76	0.39	0.7004	0.0119
WT	-3.6	2.86	108	-9.27	2.08	-1.26	0.2119	0.0095
Male	-5.63	3.38	108	-12.33	1.06	-1.67	0.0983	0.0085
Social	-8.03	2.99	108	-13.96	-2.1	-2.69	0.0084	0.0068
ELA:AT	-1.5	4.62	108	-10.65	7.65	-0.32	0.7459	0.0059
ELA:WT	0.11	3.79	108	-7.39	7.62	0.03	0.9763	0.0045
ELA:Male	-6.44	4.32	108	-15	2.13	-1.49	0.1392	0.0044
AT:Male	4.71	5.01	108	-5.23	14.64	0.94	0.3498	0.0043
WT:Male	4.19	4.19	108	-4.11	12.5	1	0.3188	0.0043
ELA:Social	3.45	3.82	108	-4.13	11.03	0.9	0.3695	0.0038
AT:Social	-3.52	4.63	108	-12.7	5.66	-0.76	0.4494	0.0037
WT:Social	2.09	3.58	108	-5.02	9.19	0.58	0.5618	0.0028
Male:Social	6.72	4.23	108	-1.66	15.1	1.59	0.115	0.002
ELA:AT:Male	7.82	6.66	108	-5.38	21.02	1.17	0.2431	0.0011
ELA:WT:Male	5.09	5.49	108	-5.79	15.97	0.93	0.356	0.001
ELA:AT:Social	3.06	5.78	108	-8.39	14.51	0.53	0.5974	0.001
ELA:WT:Social	-0.68	4.74	108	-10.07	8.71	-0.14	0.8866	0.0006
ELA:Male:Social	-2.87	5.41	108	-13.58	7.85	-0.53	0.5973	0.0005
AT:Male:Social	-2.28	6.27	108	-14.71	10.15	-0.36	0.7171	0.0004
WT:Male:Social	-5.91	5.24	108	-16.3	4.47	-1.13	0.2616	0.0001
ELA:AT:Male:Social	-9.61	8.33	108	-26.13	6.91	-1.15	0.2513	0
ELA:WT:Male:Social	-0.76	6.87	108	-14.38	12.86	-0.11	0.9122	0

Model formula:
lme(~ Group*Strain*Sex*Compartment, random = ~ 1|ID/Compartment)
$R^2_{marg} = 32.2\%$, $R^2_{cond} > 94.3\%$
Abbreviations: $\beta = \beta$ coefficient estimate, SE = standard error, Df = degrees of freedom, CI = 95% confidence interval, part = partial, group = early life condition group, marg = marginalized, cond = conditioned,

Supplementary Table 37: **ANOVA Speed [mm/sec] Social Zone**

**Model Terms**	**Df**$_{num}$	$\boldsymbol{F}$	***GES***	$\boldsymbol{p}$	$\epsilon^2_{partial}$			**Cohen's F**$_{partial}$		
					**Estimate**	**CI**$_{low}$	**CI**$_{up}$	**Estimate**	**CI**$_{low}$	**CI**$_{up}$
Group	1	6.81	0.0369	**0.01037**	0.0506	0.0011	0.1508	0.251	0.0576	0.442
Strain	2	3.25	0.0353	**0.04262**	0.0393	0	0.1236	0.2453	0	0.4218
Sex	1	17.03	0.0875	7^{-5}	0.1282	0.0339	0.2505	0.3971	0.2004	0.5921
Group:Strain	2	0.45	0.0051	0.63785	-0.0101	0	0	0.0914	0	0.2477
Group:Sex	1	7.19	0.0389	**0.0085**	0.0537	0.0018	0.1554	0.258	0.0649	0.4491
Strain:Sex	2	2.67	0.0292	*0.07397*	0.0294	0	0.1062	0.2223	0	0.3971
Group:Strain:Sex	2	0.6	0.0068	0.54796	-0.0072	0	0	0.1058	0	0.2661
Compartment	1	53.48	0.1624	$< 1^{-5}$	0.325	0.1889	0.4489	0.7037	0.4917	0.9129
Group:Compartment	1	0.81	0.0029	0.36912	-0.0017	0	0	0.0868	0	0.2754
Strain:Compartment	2	3.22	0.0228	**0.04378**	0.0388	0	0.1228	0.2442	0	0.4207
Sex:Compartment	1	0.12	0.0004	0.72637	-0.0081	0	0	0.0338	0	0.2102
Group:Strain:Compartment	2	0.08	0.0006	0.92162	-0.017	0	0	0.0389	0	0.1478
Group:Sex:Compartment	1	3.24	0.0116	*0.07449*	0.0202	0	0.0997	0.1733	0	0.3629
Strain:Sex:Compartment	2	2.36	0.0168	0.09912	0.0242	0	0.0961	0.2091	0	0.3829
Group:Strain:Sex:Compartment	2	0.82	0.0059	0.44314	-0.0033	0	0	0.1232	0	0.2871

Model formula:
lme(~ Group*Strain*Sex*Compartment, random = ~ 1|ID/Compartment)
$Df_{denBetween} = 108$, $MSE_{between} = 48.6$, $Df_{denWithin} = 108$, $MSE_{within} = 31.3$
Abbreviations: group = early life condition group, num = numerator, den = denominator, Df = degrees of freedom, *MSE* = mean standard error, *GES* = generalized η^2, CI = 95% confidence interval

Supplementary Table 38: **Descriptive Statistics of Glucocorticoid Signaling Regulating Genes Separated by Brain Region, *Fkbp5*-Genotype and Early Life Condition**

Tissue	Strain	Group	Gene	N	Mean	SD	95% CI low	95% CI up
Hypothalamus	CG	Control	*Nr3c2*	5	4.32	0.98	3.10	5.54
	CG	ELA		5	4.20	0.25	3.89	4.51
	AT	Control		4	4.26	0.41	3.62	4.91
	AT	ELA		7	3.74	1.54	2.32	5.16
Ventral Hippocampus	CG	Control		4	26.58	3.88	20.41	32.76
	CG	ELA		5	19.32	8.35	8.95	29.69
	AT	Control		3	21.26	7.60	2.37	40.15
	AT	ELA		7	13.93	5.79	8.57	19.28
Dorsal Hippocampus	CG	Control		4	26.58	3.88	20.41	32.76
	CG	ELA		5	19.32	8.35	8.95	29.69
	AT	Control		3	21.26	7.60	2.37	40.15
	AT	ELA		7	13.93	5.79	8.57	19.28
Hypothalamus	CG	Control	*Nr3c1*	5	8.32	4.85	2.30	14.35
	CG	ELA		5	8.95	2.62	5.70	12.21
	AT	Control		4	7.36	4.36	0.42	14.30
	AT	ELA		7	7.41	3.30	4.36	10.46
Ventral Hippocampus	CG	Control		4	9.30	0.87	7.93	10.68
	CG	ELA		5	8.46	2.14	5.81	11.11
	AT	Control		3	9.74	3.32	1.49	17.99
	AT	ELA		7	6.54	3.59	3.22	9.86
Dorsal Hippocampus	CG	Control		4	9.30	0.87	7.93	10.68
	CG	ELA		5	8.46	2.14	5.81	11.11
	AT	Control		3	9.74	3.32	1.49	17.99
	AT	ELA		7	6.54	3.59	3.22	9.86
Hypothalamus	CG	Control	*Hsp90ab1*	5	719.29	58.80	646.29	792.29
	CG	ELA		5	747.51	27.66	713.16	781.85
	AT	Control		4	645.67	46.85	571.11	720.22
	AT	ELA		7	642.06	54.79	591.39	692.73
Ventral Hippocampus	CG	Control		4	772.59	32.21	721.34	823.84
	CG	ELA		5	768.25	51.96	703.73	832.77
	AT	Control		3	707.60	63.99	548.63	866.58
	AT	ELA		7	645.62	98.51	554.52	736.73
Dorsal Hippocampus	CG	Control		4	772.59	32.21	721.34	823.84
	CG	ELA		5	768.25	51.96	703.73	832.77
	AT	Control		3	707.60	63.99	548.63	866.58
	AT	ELA		7	645.62	98.51	554.52	736.73

Hypothalamus	CG	Control	*Fkbp5*	5	2.80	0.61	2.04	3.56
	CG	ELA		5	2.65	0.43	2.12	3.18
	AT	Control		4	3.20	0.47	2.45	3.95
	AT	ELA		7	2.47	1.40	1.18	3.77
Ventral Hippocampus	CG	Control		4	8.59	1.98	5.44	11.75
	CG	ELA		5	7.18	2.53	4.04	10.32
	AT	Control		3	5.92	0.36	5.03	6.81
	AT	ELA		7	4.57	1.97	2.75	6.39
Dorsal Hippocampus	CG	Control		4	8.59	1.98	5.44	11.75
	CG	ELA		5	7.18	2.53	4.04	10.32
	AT	Control		3	5.92	0.36	5.03	6.81
	AT	ELA		7	4.57	1.97	2.75	6.39
Hypothalamus	CG	Control	*Fkbp4*	5	118.83	14.68	100.60	137.06
	CG	ELA		5	122.42	5.26	115.89	128.95
	AT	Control		4	118.67	17.79	90.36	146.97
	AT	ELA		7	122.26	11.40	111.72	132.80
Ventral Hippocampus	CG	Control		4	79.13	8.88	65.00	93.26
	CG	ELA		5	91.14	16.06	71.19	111.09
	AT	Control		3	83.57	16.58	42.40	124.75
	AT	ELA		7	79.59	11.16	69.27	89.92
Dorsal Hippocampus	CG	Control		4	79.13	8.88	65.00	93.26
	CG	ELA		5	91.14	16.06	71.19	111.09
	AT	Control		3	83.57	16.58	42.40	124.75
	AT	ELA		7	79.59	11.16	69.27	89.92

Supplementary Table 39: **Significant Results of the ANOVA on Tissue-Wise Expression of Glucocorticoid Signaling Regulators in Dependence of *Fkbp5*-Genotype × Early Life Condition**

Tissue	Gene	Term	Df	F	p	R^2	R^2_{adj}
Hypothalamus	*Hsp90ab1*	Strain	1	17.7	.0006	52	43
Ventral Hippocampus	*Hsp90ab1*	Strain	1	8.9	.009	43	32
	Nr3c2	Early Life	1	5.4	.03	40	28
	Fkbp5	Strain	1	7.9	.01	43	32
Dorsal Hippocampus	*Hsp90ab1*	Strain	1	8.9	0.009	43	32
	Nr3c2	Strain	1	5.4	0.03	40	28
	Fkbp5	Strain	1	7.9	0.01	43	32

Supplementary Table 40: **Top 10 Pearson Correlations between DEGs, Behavior, Physiology and *Fkbp5* Expression**

**Hypothalamus (n_{obs} = 21, n_{comp} = 284)**					
**A**	**B**	**Estimate**	p	p_{FDR}	**Comparison**
Fkbp5	*Morf4l2*	0.79	1.72^{-4}	1.64^{-3}	SNP
	Irf3	0.77	4.05^{-5}	1.81^{-3}	
	Afmid	0.76	6.52^{-5}	1.81^{-3}	
	1500009L16Rik	0.76	6.69^{-5}	1.81^{-3}	
	Slc35a5	0.76	6.65^{-5}	1.81^{-3}	
	Tigd5	0.76	7.33^{-5}	1.81^{-3}	
	Lonp2	-0.76	7.61^{-5}	1.81^{-3}	
Morning Corticosterone	*Pkmyt1*	0.76	6.83^{-5}	1.81^{-3}	
	Chrna7	0.76	5.66^{-5}	1.81^{-3}	
Time in Dark	*Gjb1*	0.76	5.48^{-5}	1.81^{-3}	ELA

**Ventral Hippocampus (n_{obs} = 19, n_{comp} = 777)**					
**A**	**B**	**Estimate**	p	p_{FDR}	**Comparison**
Fkbp5	*Cyba*	-0.88	5.58^{-7}	1.45^{-4}	SNP
	Perp	0.85	3.78^{-6}	2.23^{-4}	
	Odf3b	0.86	2.47^{-6}	2.23^{-4}	
	Zfp346	0.85	4.30^{-6}	2.23^{-4}	
	Islr	0.85	3.14^{-6}	2.23^{-4}	
	Rftn1	0.85	4.23^{-6}	2.23^{-4}	ELA
	Gprc5c	0.85	4.04^{-6}	2.23^{-4}	
	Sertad3	0.86	2.43^{-6}	2.23^{-4}	
	Dnaaf3	0.86	2.06^{-6}	2.23^{-4}	
	Lrrc7	0.86	3.06^{-6}	2.23^{-4}	

**Dorsal Hippocampus (n_{obs} = 21, n_{comp} = 852)**					
**A**	**B**	**Estimate**	p	p_{FDR}	**Comparison**
Fkbp5	*Itprid2*	0.92	3.64^{-9}	3.11^{-6}	SNP
	Lgi4	0.87	2.32^{-7}	3.31^{-5}	
	St8sia5	0.89	9.13^{-8}	3.31^{-5}	
	Ndrg4	0.88	1.60^{-7}	3.31^{-5}	
	Btd	-0.88	1.95^{-7}	3.31^{-5}	
	Becn1	-0.88	1.43^{-7}	3.31^{-5}	
	Htr1a	0.87	3.37^{-7}	3.77^{-5}	
	Stxbp5	0.87	3.87^{-7}	3.77^{-5}	
	Bicd2	0.86	5.92^{-7}	3.77^{-5}	
	Nanp	0.86	4.30^{-7}	3.77^{-5}	

Abbreviations: n_{obs} = number of observations (mice), n_{comp} = number of correlations > |0.6|, FDR = false discovery rate (5%)
